# The Training of Medium- to Long-Distance Sprint Performance in Football Code Athletes: A Systematic Review and Meta-analysis

**DOI:** 10.1007/s40279-021-01552-4

**Published:** 2021-09-09

**Authors:** Ben Nicholson, Alex Dinsdale, Ben Jones, Kevin Till

**Affiliations:** 1 Carnegie Applied Rugby Research (CARR) Centre, Carnegie School of Sport, Leeds Beckett University, Headingley Campus, Leeds, LS6 3QS UK; 2 Leeds Rhinos Rugby League Club, Leeds, UK; 3 England Performance Unit, The Rugby Football League, Leeds, UK; 4 School of Science and Technology, University of New England, Armidale, NSW Australia; 5 Division of Exercise Science and Sports Medicine, Department of Human Biology, Faculty of Health Sciences, The University of Cape Town and the Sports Science Institute of South Africa, Cape Town, South Africa

## Abstract

**Background:**

Within the football codes, medium-distance (i.e., > 20 m and ≤ 40 m) and long-distance (i.e., > 40 m) sprint performance and maximum velocity sprinting are important capacities for success. Despite this, no research has identified the most effective training methods for enhancing medium- to long-distance sprint outcomes.

**Objectives:**

This systematic review with meta-analysis aimed to (1) analyse the ability of different methods to enhance medium- to long-distance sprint performance outcomes (0–30 m, 0 to > 30 m, and the maximum sprinting velocity phase [*V*_max_]) within football code athletes and (2) identify how moderator variables (i.e., football code, sex, age, playing standard, phase of season) affected the training response.

**Methods:**

We conducted a systematic search of electronic databases and performed a random-effects meta-analysis (within-group changes and pairwise between-group differences) to establish standardised mean differences (SMDs) with 95% confidence intervals and 95% prediction intervals. This identified the magnitude and direction of the individual training effects of intervention subgroups (sport only; primary, secondary, tertiary, and combined training methods) on medium- to long-distance sprint performance while considering moderator variables.

**Results:**

In total, 60 studies met the inclusion criteria (26 with a sport-only control group), totalling 111 intervention groups and 1500 athletes. The within-group changes design reported significant performance improvements (small–moderate) between pre- and post-training for the combined, secondary (0–30 and 0 to > 30 m), and tertiary training methods (0–30 m). A significant moderate improvement was found in the *V*_max_ phase performance only for tertiary training methods, with no significant effect found for sport only or primary training methods. The pairwise between-group differences design (experimental vs. control) reported favourable performance improvements (large SMD) for the combined (0 to > 30 m), primary (*V*_max_ phase), secondary (0–30 m), and tertiary methods (all outcomes) when compared with the sport-only control groups. Subgroup analysis showed that the significant differences between the meta-analysis designs consistently demonstrated a larger effect in the pairwise between-group differences than the within-group change. No individual training mode was found to be the most effective. Subgroup analysis identified that football code, age, and phase of season moderated the overall magnitude of training effects.

**Conclusions:**

This review provides the first systematic review and meta-analysis of all sprint performance development methods exclusively in football code athletes. Secondary, tertiary, and combined training methods appeared to improve medium-long sprint performance of football code athletes. Tertiary training methods should be implemented to enhance *V*_max_ phase performance. Nether sport-only nor primary training methods appeared to enhance medium to long sprint performance. Performance changes may be attributed to either adaptations specific to the acceleration or *V*_max_ phases, or both, but not exclusively *V*_max_. Regardless of the population characteristics, sprint performance can be enhanced by increasing either the magnitude or the orientation of force an athlete can generate in the sprinting action, or both.

**Trial Registration:**

OSF registration https://osf.io/kshqn/.

**Supplementary Information:**

The online version contains supplementary material available at 10.1007/s40279-021-01552-4.

## Key Points

**Table:** 

Research evaluating the medium- to long-distance sprint performance in the football codes is biased towards male soccer athletes involved in tertiary training methods (e.g., strength, power, and plyometrics training).
Medium- to long-distance sprint performance of football code athletes can be enhanced through secondary (i.e., resisted or assisted sprinting), combined (i.e., primary or secondary and tertiary methods) (0–30 and 0–>30 m), and tertiary training methods (0–30 m). Tertiary training methods were the only mode to significantly enhance the maximum velocity phase performance. However, sport-only training or primary training methods did not enhance performance. Despite the use of performance outcomes >20 m as a proxy measure of maximum velocity performance, performance changes may be attributed to either or both adaptations specific to the acceleration or maximum velocity phases, not exclusively maximum velocity.
Independent of the population characteristics, findings suggest that practitioners should develop either the magnitude or the orientation of forces, or both, that an athlete can generate and express in the sprinting action to improve medium- to long-distance sprint performance.

## Introduction

Football athletes are defined as those who are competing within a football code. These typically include soccer, American football, Canadian football, Australian football, rugby union, rugby league, rugby sevens, Gaelic football, and futsal. Football code athletes should be proficient at sprinting both short (i.e., 5–20 m) and medium–long (> 20 m) distances [1–5]. Although less frequent, players also perform medium- (i.e., > 20 and ≤ 40 m) to long-distance sprints (e.g., > 40 m), enabling athletes to express maximum sprinting velocity (*V*_max_) capabilities, particularly from moving starts [4, 6–14]. Very large associations have been demonstrated between *V*_max_ and sprint performance (0–36.6 m, *r* = 0.94; 18.3–36.6 m, *r* = 0.97) in football code athletes, whereas the relative rate of acceleration remained the same irrespective of sprinting performance, indicating that a higher *V*_max_ enables a superior acceleration performance [8]. Given that most athletes accelerate in a similar manner relative to *V*_max_, it may be that *V*_max_ serves as the upper threshold or limiting factor in the acceleration phase performance. Therefore, improving an athlete’s sprinting *V*_max_ may indirectly improve acceleration [8]. Hence, the development of *V*_max_ and medium–long sprint performance is a vital component of athletic performance within the football codes [15–18].

Sprint performance over distances greater than 20 m (i.e., 0–30 and 0–40 m split time or velocity) has been shown to be a differentiating factor between playing standards [19–21] and age categories [19, 21, 22] and is associated with success in key attacking and defensive performance indicators in football code athletes (e.g., rugby sevens [16], rugby league [17, 18], soccer [23]). This body of evidence emphasises the importance of sprint performance for football performance and player development. Unlike sprinters or non-athletic populations, sprint performance development programmes in football code athletes are typically performed concurrently with multiple other potentially contrasting physical capacities (e.g., endurance) alongside the code’s specific technical–tactical skills. Therefore, developing sprint performance is a challenge for all practitioners involved in the football codes [15, 19, 24]. The review by Nicholson et al. [25] reported that short-sprint performance outcomes (0–5, 0–10, and 0–20 m) were enhanced concurrently with code-specific training in football code athletes, but no research has identified the most effective training methods for enhancing medium- to long-distance sprint outcomes in football code athletes (e.g., 0–30, 0–40, 0–50 m). This highlights the need for specifically targeted sprint-based research to understand the most effective, evidence-based methods for developing sprint performance over medium to long sprint distances (e.g., 30–50 m).

Sprinting is a multidimensional skill with distinct phases (e.g., acceleration and *V*_max_). The sequential phases present shifting kinetic and kinematic outcomes as running velocity increases [26]. The kinetic changes include a reduction in the relative contribution of horizontal and increasing contribution of vertical ground reaction forces [26]. Kinematic outcomes include progressively greater stride length and frequency, reduced contact times, and the trunk lean becoming closer to vertical [26]. As a population, football code athletes exhibit different physical and technical approaches to sprinting [27, 28] when compared with well-trained sprinters. Notably, *V*_max_ is achieved at shorter distances (e.g., 15–40 vs. 40–60 m, respectively) with a lower *V*_max_ (~ 7–10 vs. > 12 m·s^−1^) compared with well-trained elite male sprinters [8, 9, 27, 29–31]. Furthermore, a higher *V*_max_ percentage is attained at shorter distances (e.g., 90% at 13.7 m in American football [8]; 96% at 21 m in rugby [9]). This highlights the need for specifically targeted sprint-based research within this population.

Previous reviews of the literature and meta-analyses [32, 33] assessing mixed population cohorts (i.e., sprinters, team sport, and non-athletic populations) and several training studies evaluating the effectiveness of sprint training interventions [34–36] reported that sprint performance is a trainable capacity. However, the responses to sprint development were reported to be highly variable [32, 34, 37, 38]. Training effects appear to be mode specific, with distance-specific performance changes (e.g., 0–30 and 0 to > 30 m) associated with phase-specific adaptations (i.e., acceleration vs. *V*_max_ [32, 33]). Training modes are typically classified based on task specificity into the following subgroups: primary (e.g., sprint technique, sprinting), secondary (e.g., resisted or assisted sprinting), or tertiary (e.g., non-specific methods, including resistance training and plyometrics) [39]. Limitations in the literature mean that the best method of enhancing medium to long sprint performance, both individually and across football codes, is currently unclear. These limitations include (1) a lack of reviews exclusively including football code athletes, instead including sprinters and non-athletes [32, 33, 40–49]; (2) a lack of studies examining all training modalities across football code athletes [32, 33, 40–49]; and (3) previous systematic reviews and meta-analyses [32, 33, 41] have misclassified training modes by failing to account for the normal training practices undertaken by training intervention groups (e.g., training categorised as a resisted sled intervention also including two strength sessions per week). These limitations heavily influence the interpretation and knowledge associated with sprint training interventions for applying evidence-based practices within football code athletes. Hence, the effective development of medium to long sprint performance is a collective problem across codes. A cross-football codes systematic review would provide a more comprehensive overview of the available literature than one focusing on an individual sport, while also comparing best methods of developing medium to long sprint performance. However, the magnitude and direction of the training response may be affected by ‘moderator’ variables, presenting changes based on population characteristics such as the sport [50], age [42], and sex [51] of the athlete and on training phase (e.g., pre-season [33]). Therefore, it is important to also identify the moderator variables and evaluate the extent that they may affect the resultant training effect [52].

This systematic review and meta-analysis aimed to (1) analyse the impact of different methods to enhance medium- to long-distance sprint performance outcomes (0–30 m, 0 to > 30 m, and the *V*_max_ phase) within football code athletes and (2) identify how moderator variables (i.e., football code, sex, age, playing standard, phase of season) affect the training response.

## Methods

### Design and Search Strategy

A systematic review and meta-analysis was conducted in accordance with the Preferred Reporting Items for Systematic Reviews and Meta-Analyses (PRISMA) statement [53] and followed the PROSPERO guidelines. Given the nature of the project, the review protocol was prospectively registered on the database for Open Science Framework (OSF: https://osf.io/kshqn/). A systematic search of electronic databases (PubMed, The Cochrane Library, MEDLINE, SPORTDiscus, and CINAHL, via EBSCOhost) was conducted to identify original research articles published from the earliest available records up to and including 4 December 2019. Boolean search phrases were used to include search terms relevant to football code athletes (population), the training intervention (dependent variable), and the sprint performance outcomes (independent variable). Relevant keywords for each search term were determined through pilot searching (screening of titles/abstracts/keywords/full texts of previously known articles). Keywords were combined within terms using the ‘OR’ operator, and the final search phrase was constructed by combining the three search terms using the ‘AND’ operator (Table 1).

**Table 1:** Database literature search strategy

Search term	Keywords
1. Sports population	“soccer” OR “football” OR “rugby” OR “futsal” (NOT/- “sprinters” OR “swimming” OR “cycling” OR “Paralympic”)
2. Training intervention	“sprinting” OR “sprint” OR “training” OR “speed” OR “resisted” OR “assisted” OR “resistance” OR “power” OR “strength” OR “plyometric” OR “weightlifting” OR “strongman” OR “technique” OR “weight” OR “sled” OR “intervention” OR “sprint mechanics”
3. Outcome measures	“sprint performance” OR “acceleration” OR “velocity”
Search phrase:	1 AND 2 AND 3

### Study Selection

Duplicate records were identified and removed, and the remaining records were screened against the predefined inclusion and exclusion criteria (Table 2). Studies were screened independently by two researchers (BN, AD). The screening of the journal articles was completed over two phases. Studies were initially excluded based on the content of the titles and abstracts, followed by a full-text review. If the reviewers’ decisions differed, reviewers met to come to an agreed decision on the paper. Disparities in study selection were resolved by a third reviewer (KT).

**Table 2:** Inclusion/exclusion criteria (title/abstract screening and full screening)

Criteria	Inclusion	Exclusion
1	Studies with human subjects and a pre- and post-outcome measure(s) identifying sprint performance > 20 m	Studies with non-human subjects and/or no pre- and post-outcome measure(s) identifying sprint performance ≤ 20 m or performance outcomes measured using stopwatches
2	Training intervention study with the training programme clearly outlined, designed to produce chronic adaptations (not acute). Interventions including specific sprint training (resisted, assisted, unresisted sprinting, sprint mechanics, and technique training), non-specific sprint training (strength, power, plyometric training, and non-traditional methods), and combined sprint training (combined specific, combined non-specific, and combined mixed methods)	Inappropriate study design: not an intervention study or an acute/post-activation study
3	Original research article	Reviews, surveys, opinion pieces, books, periodicals, editorials
4	Population: football code athletes. Football athletes defined as those who are competing within a football code. Football codes for inclusion: soccer, American football, Canadian football, Australian football, rugby union, rugby league, rugby sevens, Gaelic football, futsal	Non-football code sports (e.g., solo, racquet/bat, or combat sports), match officials, or non-athletic populations
5	Healthy, able-bodied, non-injured athletes	Special populations (e.g., clinical, patients), athletes with a physical or mental disability, and athletes considered to be injured or returning from injury

### Data Extraction

One author (BN) extracted the following data using a specifically designed standardised Microsoft Excel spreadsheet: general study information (i.e., author, year), subject characteristics (i.e., sample size, sex, age, body mass, height, sport, training status, performance level), training intervention characteristics (i.e., training methods, control group information, number of sessions per week, duration of training intervention, total amount of training sessions, training intensity, training volume, testing distances, testing equipment, training surface, other training, reported training-related injuries), and primary outcome measures (i.e., pre- and post-training intervention means and standard deviations [SDs]). All studies that included the time or velocity achieved from the initial start position (0 m) to between > 20 and ≤ 30 m and between 0 and > 30 m were categorised into the 0–30 m and 0 to > 30 m subgroups, respectively. The *V*_max_-phase subgroup included directly measured *V*_max_ achieved or time to completion for distances > 20 m with a maximum intensity run-in distance of ≥ 20 m before recording time (e.g., 20–30 or 30–40 m). These outcomes aimed to identify distance-specific changes, whilst representing the longer sprint distances (0 to 30–50 m) performed by football code athletes and those commonly measured by researchers/practitioners. Descriptive information relating to the training activities performed in the studies was used to categorise each intervention into the training mode subgroups outlined in Table 3. If the pre- and post-outcome measure data were not available from the tables or the results section, the data were requested from the author(s). If the authors did not have access to these data, we extracted data on outcome measures from figures using WebPlotDigitizer version 4.1 software (2018). Means and SDs/standard error of the mean were measured manually at the pixel level to the scale provided in the study’s figures.

**Table 3:** Subgroup categorisation

Specific sprint training: training methods in which the athlete is simulating/performing the sprint movement pattern (see primary and secondary methods)		Tertiary methods (non-specific sprint training): training methods not involving the athlete sprinting, that have a transfer into sprint performance as a result of the subsequent training adaptations (e.g., strength, power, plyometric training). These may be performed individually (e.g., strength training) or in combination with other tertiary methods (e.g., strength, power, and plyometric training)
Combined specific methods: training methods that included both primary and secondary methods (e.g., sprinting + resisted sled sprinting)		
Primary methods: training methods simulating the sprint movement pattern (sprint-technique drills, stride length and frequency exercises, and sprints of varying distances and intensities)	Secondary methods: training methods simulating the sprint action but applying overload by reducing or increasing the speed of the movement by applying additional resistance (e.g., sledges, resistance bands, weighted garments or incline sprints [gravity resisted]) or assistance (e.g., pulley systems, partner assisted or decline sprints [gravity assisted])	
Combined training: training methods that included both specific sprint training (primary and or secondary methods) and tertiary methods in combination (e.g., strength, power, resisted, and unresisted sprint training)		
Sport only training: training methods not including any specific or non-specific sprint training. This is described as a format of offensive, defensive, and match simulation technical and tactical drills, which may include some form of endurance training and or competitive games		

Subgroup categories are based on previous definitions from Plisk [39] and Rumpf et al. [32]

### Study Quality Assessment

The quality of the included studies was assessed using the same scale as in McMaster et al. [54]. This scale is designed to evaluate research conducted in athletic-based training environments from a combination of items from the Cochrane, Delphi, and PEDRO scales. The methodological scale assesses the study in the following ten domains: inclusion criteria stated, subject assignment, intervention description, control groups, dependent variables definition, assessment methods, study duration, statistics, results section, and conclusions. Each domain was assigned a score of either 0 indicating clearly no, 1 indicating maybe, or 2 indicating clearly yes. The scores were then summed to assess the total study quality out of a maximum of 20.

### Data Analysis and Meta-analyses

Data extracted from the systematic search were included in the meta-analyses. Improvements in sprint performance are typically identified by a reduction in time taken to cover a given distance or an increase in *V*_max_ achieved for a given time point and or distance [55, 56]. Therefore, pre- and post-time changes were reversed before conducting the analysis. This enabled both time and velocity changes to represent the same direction, thus identifying a reduction in time or an increase in velocity for a given distance as a positive change.

A random-effects meta-analysis was performed using Comprehensive Meta-Analysis Version 3.0 software (Biostat, Englewood, NJ, USA) to assess the magnitude of change in the outcomes across the relevant primary studies and to explore the effect of moderator variables on the variation among study outcomes [57]. This included implementing two meta-analysis approaches: (1) pre-and post-training within-group changes and (2) pairwise between-group effect difference designs. This approach provides an extensive review of all the available training intervention literature for developing sprint performance in football code athletes, including multiple research designs with and without sport-only control groups. In the between-group pairwise analysis, for the studies with multiple intervention groups and single control groups [35, 36, 58–68], the control samples were split into two or more groups of smaller sample sizes to enable two or more (reasonably independent) experimental comparisons [69]. This aligns with our extensive design to evaluate all available literature without combining or removing distinct subgroups (e.g., primary and tertiary methods [67]). Overall summary estimates were calculated for each of the training type subgroups: primary, secondary, combined specific, tertiary, combined methods, and sport-only training (Table 3). We conducted a meta-analysis to identify the between-comparator group (e.g., primary vs. sport only, tertiary vs. sport only) adjusted mean performance effects when a sport-only comparator group was available. Combining a within-group pre-post change design and pairwise between-group differences enabled an evaluation of both high-quality controlled trial studies to evaluate training causality and to explore the breadth of the available literature using a range of research designs.

Outcome measures were converted into standardised mean differences (SMDs) with 95% confidence intervals (CI) (used as the summary statistic) and 95% prediction intervals (PI). The SMD represents the size of the effect of the intervention relative to the variability observed in that intervention. An inverse-variance random-effects model was used for the meta-analysis because it allocated a proportionate weight to trials based on the size of their individual standard errors and facilitated analysis while controlling for heterogeneity across studies [70]. The inputted data included sample sizes, outcome measures with their respective SDs, and a correlation coefficient for within-subject measurements. These correlation coefficients (0–30 m, *r* = 0.92; 0 to > 30 m, *r* = 0.92; and *V*_max_ phase, *r* = 0.95) were estimated from prior field testing. The SMD values were interpreted as follows: < 0.20 as trivial, 0.20–0.39 as small, 0.40–0.80 as moderate, and > 0.80 as large [71]. A positive SMD indicated that the training intervention was associated with an improvement in sprint performance, whereas a negative SMD indicated a decrease in the respective performance outcome. Accompanying *p* values tested the null hypothesis that there was no statistically significant change in sprint performance regardless of the training method. Statistical significance was considered for *p* < 0.05. Heterogeneity between trials was assessed using the *I*^2^ statistic, with moderate (> 50%) to high (> 75%) values used to indicate potential heterogeneity sources [72]. The *I*^2^ statistic was supported by reporting the Tau-squared statistic and the Chi-squared statistic. Sensitivity analyses were conducted for each subgroup by repeating the analyses with each study omitted in turn; this examined whether any conclusions were dependent on a single study.

Subgroup analyses were performed to (1) compare the within-group change in pre- and post-sprint performance and pairwise between-group effects from comparative trials and (2) evaluate the potential moderator variables. The moderator variables were determined a priori: sex (male vs. female), football code, playing standard (elite vs. sub-elite [from Swann et al. [73], the highest reported standard of performance]), age category (senior [mean age ≥ 18 years] vs. youth [mean age < 18 years]), and training phase (pre-season vs. in-season vs. off-season).

### Evaluation of Small Study Effects

Small study effects were explored through visual interpretation of funnel plots of SMD versus standard errors and by quantifying Egger’s linear regression intercept [74] to evaluate potential bias. A statistically significant Egger’s statistic (*p* value < 0.05) indicated the presence of a small study effect.

## Results

### Overview

**Fig. 1:**
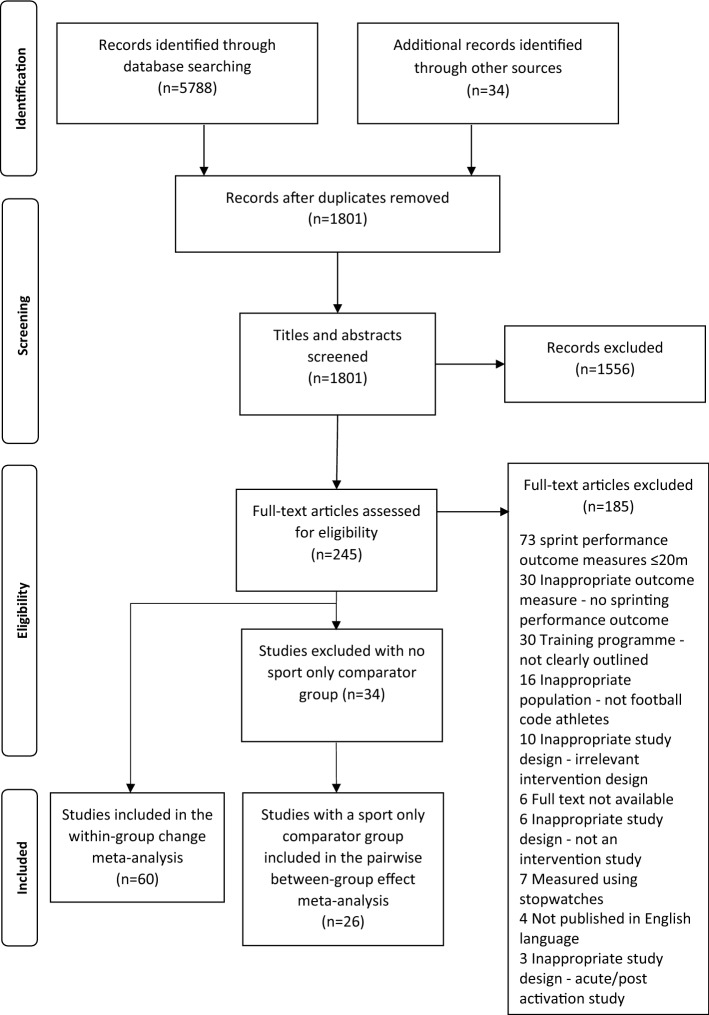
Flow diagram of the process of study selection

After duplicates were removed, 1801 studies remained. The study selection inclusion criteria identified 60 studies for inclusion in the within-group change meta-analysis and 26/60 studies for inclusion in the pairwise between-group analysis (Fig. 1). The 60 studies [34–36, 58–68, 75–120] included multiple different research designs (with and without experimental control groups), providing 111 intervention groups and 27 sport-only groups. Training groups were sub-grouped into six training classifications (sport only, *n* = 27; combined methods, *n* = 35; primary methods, *n* = 8; secondary methods, *n* = 9; tertiary methods, *n* = 59; and combined specific *n* = 0) to differentiate between findings for distinct sprint performance outcomes (Table 3). The 26 identified studies compared a training intervention with a sport-only (i.e., control) comparator group [35, 36, 58–68, 75, 82, 88, 90, 92, 95–97, 104, 106, 113, 114, 119]. This provided 41 eligible training groups for pairwise between-group comparisons (sport-only training vs. combined methods, *n* = 9; primary methods, *n* = 3; secondary methods, *n* = 2; and tertiary methods, *n* = 27).

Table S1 (non-specific/tertiary, *n* = 59), Table S2 (combined, *n* = 35), and Table S3 (specific, *n* = 17) (all in the electronic supplementary material [ESM]) present the individual training group study descriptives, training interventions, and sprint outcomes for the included studies. The 60 studies [34–36, 58–68, 75–120] represented a total sample of 1500 football code athletes with a mean sample size of 11.1 ± 3.9 participants per training group. In total, 56 studies were conducted in male athletes, three studies were in female athletes [86, 106, 116], and one was in a mixed population [65]. The mean age of the participants included in the studies ranged from 11 to 26.8 years. The athlete populations ranged from sub-elite to elite [73]. Collectively, the training intervention durations ranged from 3 to 22 weeks (7.4 ± 3.1 weeks), with the intervention training frequency ranging between one and four sessions per week (2.1 ± 0.6) over 6–32 individual sessions.

Studies were conducted in soccer (*n* = 43), rugby league (*n* = 4), rugby union (*n* = 4), rugby sevens (*n* = 3), American football (*n* = 1), Australian football (*n* = 1), and mixed football codes (*n* = 4). No studies in futsal or Gaelic football players satisfied the inclusion criteria. Studies were conducted in pre-season (*n* = 21), in-season (*n* = 26), or off-season (*n* = 3) periods, and across pre-season and in-season periods (*n* = 2). Eight studies did not report the phase of the season. Sprint assessment distances ranged from 22.9 to 50 m (0–30 m [*n* = 46], 0 to > 30 m [*n* = 20], and *V*_max_ phase [*n* = 13]). Timing devices included electronic timing gate systems (*n* = 52), high-speed video cameras (*n* = 3), radar measurement devices (*n* = 2), 1080 sprint device (*n* = 2), a digital timing device (*n* = 1), a laser measurement device (*n* = 1), a kinematic measurement system (*n* = 1), and a mobile application (mysprint; *n* = 1).

Sport-only training groups were described as some format of offensive or defensive match simulation and technical and tactical drills performed over two to ten sessions per week across 2–6 days per week lasting between 30 and 120 min per session as well as some form of endurance training and one to two competitive or friendly games per week. Various methods of endurance training were described, including simulated games performed in small-, medium-, or large-sided games formats (e.g., 3 vs. 3–11 vs. 11), low-intensity aerobic conditioning, high-intensity interval training, and recreational or cardiovascular activities (e.g., basketball, biking, running, aerobics). Sport-only training was conducted in both pre-season and in-season periods over a duration of 6–16 weeks.

Specific sprint-training groups completed sprinting, resisted and assisted sprinting, and technical sprint drills as individual modalities and/or in combination (e.g., complex and contrast sets). The training was performed 1–3 days per week, with intervention periods lasting from 4 to 8 weeks (8–21 sessions). The primary sprint-training methods included single-set interventions ranging from 8–10 repetitions of short-distance sprints (18.3–20 m; 160–183 m session totals) to 4–6 repetitions of long-distance springs (200 m; 800–1200 m session totals). Multiple-set methods ranged from 2–6 sets of 2–8 repetitions of medium- to long-distance sprints (30–50 m; 120–1200 m session totals). One study performed submaximal sprint efforts (85% *V*_max_), involving 4–6 sets of 4 repetitions of long sprints (50 m; 800–1200 m session totals) [102]. Resisted sprinting was performed as either a single set of 3–10 repetitions of short-distance sprints (18.3–20 m; 60–200 m session total) or multiple-set methods, ranging from 2 to 7 sets of 3–5 repetitions of short-medium distance resisted sled sprints (5–40 m; 130–455 m session totals). Resisted sprint loads ranged from light to very heavy loads [44]. Loads were prescribed based on percentage body mass (BM) (i.e., 10–80% BM). Assisted sprinting methods involved both single and multi-set methods. The single-set intervention included 1 set of 10 repetitions of short sprints over 18.3 m with a bungee cord assistive load at 14.7% BM (183 m session total [116]). Multi-set methods ranged from 1 to 3 sets of 3 repetitions of medium-distance sprints (40 m) with towing eliciting a 0.5- to 1-s faster 0–40 m time using a sprint master towing device (120–360 m session total) [101]. The same study used a combined study arm using the same assistance load while also wearing a 10-lb weighted vest.

Tertiary sprint-training groups consisted of strength, power, and/or plyometrics training performed as individual modalities and/or in combination (e.g., complex and contrast sets). The training was performed 1–4 days per week, with intervention periods lasting from 4 to 22 weeks (8–32 sessions). Lower body strength training (e.g., squat, hip hinge, and calf raise variations) ranged from moderate to supramaximal loads (55–110% one-repetition maximum [1RM]) with low- to high-volume training (e.g., 2–6 sets of 2–6 repetitions and/or 2–6 sets of 8–30 repetitions). Power sessions consisted of ballistic (e.g., squat jump) and Olympic weightlifting-type exercises (e.g., clean/snatch derivatives) at low to heavy loads (15–80% 1RM to + 30% BM) and velocity-based training using loads corresponding to the mass at which optimal power is produced (1–1.1 × optimal power load). Volume ranged from 2 to 5 sets of 2–12 repetitions. Plyometrics sessions involved low- to high-intensity plyometrics (e.g., ankle hops to 50 cm accentuated eccentric loading drop jump at + 20% BM) for 1–12 sets of 4–20 repetitions (20–260 foot contacts session totals). The only type of surface identified was a grass surface. Several of the sessions were performed in combination with upper body training.

Combined methods training groups consisted of various formats of both specific sprint training (primary and/or secondary methods) and tertiary methods in combination (e.g., strength, power, resisted and unresisted sprint training). These were completed as individual modalities and/or in combination (e.g., complex and contrast sets). The training was performed 1–4 days per week, with intervention periods lasting from 3 to 15 weeks (6–22 sessions). Strength training ranged from moderate to supramaximal loads (70–120% 1RM) with low to high volume (e.g., 2–6 sets of 2–6 repetitions and/or 3–4 sets of 8–12 repetitions). Power training consisted of ballistic (e.g., squat jump) and Olympic weightlifting-type exercises (e.g., clean/snatch derivatives) at light to heavy loads (20–86% 1RM) and/or velocity-based training using loads corresponding to the mass at which optimal power is produced (1–1.1 × optimal power load or 0.8–1.2 m·s^−1^ loads). This also included medicine ball throws of 3–12 kg. Volume ranges were from 2 to 6 sets of 2–8 repetitions per set. Plyometrics sessions involved low- to high-intensity plyometrics (e.g., ankle hops to 75 cm hurdle jumps), with 2–5 sets of 1–10 repetitions (9–250 foot contacts session totals). The only type of surface identified was a synthetic grass pitch. The specific sprint-training methods included single-set interventions ranging from 1 to 8 repetitions of short- to long-distance sprints (5–45.72 m) or multiple-set methods, ranging from 1 to 5 sets of 3–7 repetitions of short- to medium-distance sprints (5–40 m; 30–800 m session totals) from various starting positions. Resisted sprint loads ranged from light to very heavy loads. Loads were prescribed based on absolute loads (i.e., 10–30 kg), percentage BM, i.e., 5–20% BM or reduction in *V*_max_ corresponding to the additional resistance applied (10–60% reduction in *V*_max_). One training study used assisted sprints, involving 1 set of five medium-distance sprints (40 m) with 25 m of each sprint including a 2% gradient decline (200 m session total [83]). Several of the sessions were performed in combination with upper body training.

### Study Quality

The scores for the assessment of study quality [54] are shown in Table 4 and ranged from 11 to 20 with a mean score of 18 ± 1.9, demonstrating high study quality. Items 2 (subjects assigned appropriately [random/equal baseline]), 4 (control group inclusion), and 9 (results detailed [mea* n* ± SD, percent change, effect size]) were the most decisive factors in separating high-quality and low-quality studies.

**Table 4:** Methodological quality scale scores

Study	Question number										Score
	1	2	3	4	5	6	7	8	9	10	
Alptekin et al. [75]	2	2	2	2	2	1	2	2	0	2	17
Barr et al. [76]	2	2	2	2	2	2	2	2	2	2	20
Beato et al. [77]	2	2	2	0	2	2	2	2	2	2	18
Bianchi et al. [78]	2	2	2	0	2	2	2	2	2	2	18
Borges et al. [79]	2	2	2	0	2	2	2	2	0	2	16
Bouguezzi et al. [80]	2	2	2	0	2	2	2	2	2	2	18
Bremec [58]	2	2	2	2	2	2	2	2	2	2	20
Chelly et al. [81]	2	2	2	2	2	2	2	2	2	2	20
Christou et al. [82]	2	2	2	2	2	2	2	2	2	2	20
Cook et al. [83]	2	2	2	0	2	2	2	2	2	2	18
Coratella et al. [59]	2	2	2	2	2	2	2	2	2	2	20
Coutts et al. [84]	2	0	2	0	2	2	2	2	0	2	14
de Hoyo et al. [85]	2	2	2	0	2	2	2	2	2	2	18
Derakhti [60]	2	2	2	2	2	2	2	2	2	2	20
Douglas et al. [34]	2	2	2	2	2	2	2	2	2	2	20
Enoksen et al. [35]	2	2	2	2	2	2	2	2	2	2	20
Escobar-Álvarez et al. [86]	2	0	2	2	2	2	2	2	1	1	16
Escobar-Álvarez et al. [87]	1	0	2	0	1	2	2	2	1	0	11
Faude et al. [88]	2	2	2	2	2	2	2	2	2	2	20
Gabbett et al. [89]	2	0	2	0	2	2	2	2	1	2	15
García-Pinillos et al. [90]	2	2	2	2	2	2	2	2	0	2	18
Gil et al. [91]	2	2	2	0	2	2	2	2	2	2	18
Hammami et al. [92]	2	2	2	2	2	2	2	2	0	2	18
Hammami et al. [93]	2	2	2	0	2	2	2	2	2	2	18
Hammami et al. [61]	2	2	2	2	2	2	2	2	2	2	20
Harris et al. [94]	2	2	2	0	2	0	2	2	0	2	14
Karsten et al. [95]	2	2	2	2	2	2	2	2	0	2	18
Krommes et al. [96]	2	2	2	2	2	2	2	2	2	2	20
Lahti et al. [36]	2	2	2	2	2	2	2	2	2	2	20
López-Segovia et al. [97]	2	2	2	2	2	2	2	2	2	2	20
Loturco et al. [98]	2	2	2	0	2	2	2	2	0	2	16
Loturco et al. [99]	2	2	2	0	2	2	2	2	2	2	18
Loturco et al. [100]	2	2	2	0	2	2	2	2	0	2	16
Majdell and Alexander [101]	2	2	2	0	2	2	2	2	0	2	16
Manouras et al. [62]	2	2	2	2	2	2	2	2	0	2	18
McMaster et al. [120]	2	2	2	0	2	2	2	2	2	2	18
Meckel et al. [102]	2	2	2	0	2	2	2	2	0	2	16
Michailidis et al. [103]	2	2	2	2	2	2	2	2	0	2	18
Negra et al. [104]	2	2	2	2	2	2	2	2	0	2	18
Orange et al. [105]	2	2	2	2	2	2	2	2	2	2	20
Ozbar [106]	2	2	2	2	2	2	2	2	2	2	20
Ramírez-Campillo et al. [63]	2	2	2	2	2	2	2	2	2	2	20
Ramírez-Campillo et al. [64]	2	2	2	2	2	2	2	2	2	2	20
Ramírez-Campillo et al. [65]	2	2	2	2	2	2	2	2	2	2	20
Ramírez-Campillo et al. [66]	2	2	2	2	2	2	2	2	1	2	19
Randell et al. [107]	2	2	2	2	2	2	2	2	2	2	20
Rey et al. [108]	2	2	2	2	2	2	2	2	2	2	20
Rimmer and Sleivert [67]	2	2	2	2	2	2	2	2	0	2	18
Rønnestad et al. [68]	2	2	2	2	2	2	2	2	0	2	18
Rønnestad et al. [109]	2	2	2	0	2	2	2	2	0	2	16
Ross et al. [110]	2	2	2	0	2	2	2	2	2	2	18
Scott et al. [111]	2	2	2	2	2	2	2	2	0	2	18
Shalfawi et al. [112]	2	2	2	2	2	2	2	2	2	2	20
Söhnlein et al. [113]	2	0	2	2	2	2	2	2	2	2	18
Tønnessen et al. [114]	2	2	2	2	2	2	2	2	2	2	20
Tous-Fajardo et al. [115]	2	0	2	2	2	2	2	2	0	2	16
Upton [116]	2	2	2	0	2	2	2	2	0	2	16
West et al. [117]	2	2	2	0	2	2	2	2	0	2	16
Winwood et al. [118]	2	2	2	0	2	2	2	2	0	2	16
Wong et al. [119]	2	0	2	2	2	2	2	2	0	2	16

0 = clear no, 1 = maybe, 2 = clear yes

### Meta-analysis

Tables S1–S3 in the ESM provide the individual study statistics.

### Standardised Mean Difference (SMD) for 0–30 m Performance

**Fig. 2:**
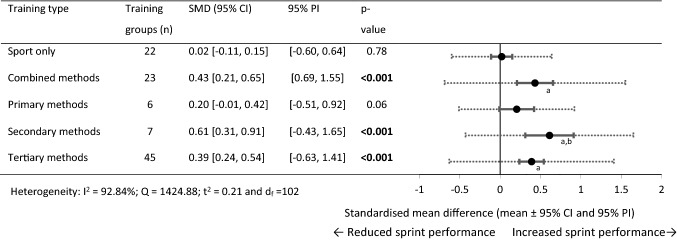
Forest plots showing the SMD (mean [95% CI and 95% PI]) for the studies evaluating the between-training-group effects on 0–30 m sprint performance. ^a^Significantly different to sport-only training, *p* < 0.05; ^b^Significantly different to primary training methods, *p* < 0.05. Bold formatting indicates *p* < 0.05. *CI* confidence interval, *PI* prediction interval, *SMD* standardised mean difference

**Fig. 3:**
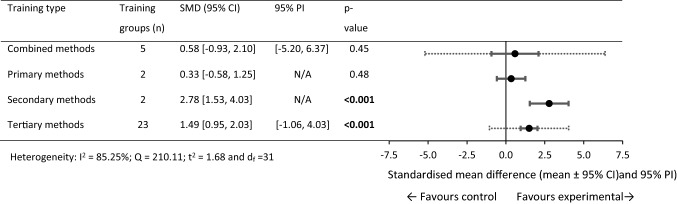
Forest plots showing the SMD (mean [95% CI and 95% PI]) in post-intervention 0–30 m sprint performance between intervention and control athletes. Bold formatting indicates *p* < 0.05. *CI* confidence interval, *N/A* fewer than three training groups available, *PI* prediction interval, *SMD* standardised mean difference

For 0–30 m performance, 103 within-training group effects were analysed from 45 original studies [34, 36, 58–60, 62–66, 75, 77–80, 82, 85–88, 90–100, 102–108, 113, 115–120]. In total, 32 training and control groups from 21 studies were eligible for a pairwise between-group analysis (sport-only control vs. experimental) [36, 58–60, 62–66, 75, 82, 88, 90, 92, 95–97, 104, 106, 113, 119]. In nine studies [36, 58–60, 62–66], the 21 available control groups were split to allow comparison between the multiple training groups in the studies [69]. Figures 2, 3 show the SMD for each training type.

#### Within-Group Changes (0–30 m)

The sport-only and primary methods training failed to show statistical significance for change in 0–30 m performance. Significant performance improvements were observed in the combined and secondary methods training groups (moderate SMD) and tertiary methods (small SMD).

The combined, secondary, and tertiary methods demonstrated a significantly larger training effect than sport-only training. Only secondary methods reported a significantly larger training effect than primary training methods.

#### Pairwise Between-Group Differences (0–30 m)

The combined and primary training methods failed to show statistical significance to sprint performance changes compared with sport-only training. Significant performance improvements were observed (large SMD) for the secondary and tertiary training groups compared with the sport-only control groups. Between-experimental subgroups analysis failed to show statistical significance between training methods. Between-experimental-subgroup analysis was not conducted on the primary or secondary subgroups with control groups because only two training groups were available.

### SMD for 0 to > 30 m Performance

**Fig. 4:**
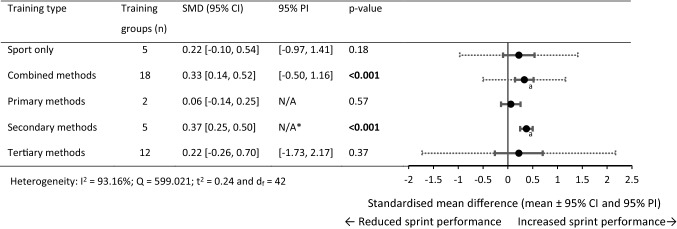
Forest plots showing the SMD (mean [95% CI and 95% PI]) for the studies evaluating the between-training-group effects on 0 to > 30 m sprint performance. ^a^Significantly different to primary training methods, *p* < 0.05. Bold formatting indicates *p* < 0.05. *CI* confidence interval, *N/A* fewer than three training groups available, N/A* all studies show a common effect size, *PI* prediction interval, *SMD* standardised mean difference

**Fig. 5:**
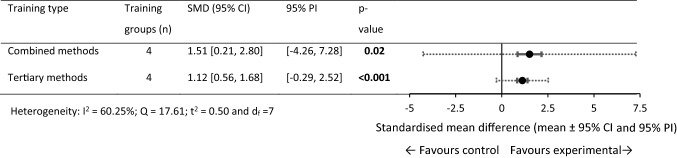
Forest plots showing the SMD (mean [95% CI and 95% PI]) in post-intervention 0 to > 30 m sprint performance between intervention and control athletes. Bold formatting indicates *p* < 0.05. *CI* confidence interval, *PI* prediction interval, *SMD* standardised mean difference

For 0 to > 30 m performance, 43 within-training group effects were analysed from 18 original studies [35, 61, 68, 76–78, 83–85, 89, 92, 101, 109–112, 114, 116]. Eight training and control groups from five studies were eligible for a pairwise between-group analysis (sport-only control vs. experimental) [35, 61, 68, 92, 114]. The five available control groups were split in three studies [35, 61, 68] to allow comparison between multiple training groups in the studies [69]. Figures 4, 5 show the SMD for each training type.

#### Within-Group Changes (0 to > 30 m)

The sport-only training, primary, and tertiary methods failed to show statistical significance for change in 0 to > 30 m sprint performance. Significant performance improvements were observed in the combined and secondary methods training groups (small SMD). Between-subgroups analysis failed to show statistical significance between training methods. Between-subgroup analysis was not conducted on the primary training methods subgroup as only two training groups were available.

#### Pairwise Between-Group Differences (0 to > 30 m)

Significant performance improvements were observed (large SMD) for the combined and tertiary training groups compared with the sport-only control groups. Between-experimental subgroups analysis failed to show statistical significance between training methods.

### SMD for Maximum-Velocity Phase Performance

**Fig. 6:**
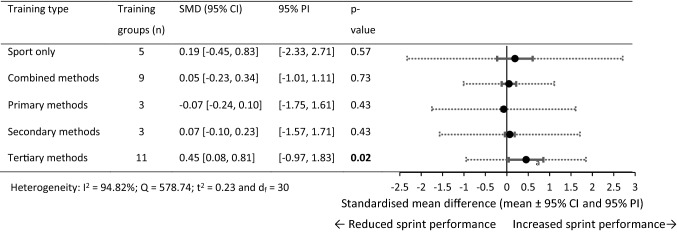
Forest plots showing the SMD (mean [95% CI and 95% PI]) for the studies evaluating the between-training-group effects on *V*_max_-phase sprint performance. ^a^Significantly different to primary training methods, *p* < 0.05. Bold formatting indicates *p* < 0.05. *CI* confidence interval, *PI* prediction interval, *SMD* standardised mean difference, *V*_max_ maximum sprinting velocity

**Fig. 7:**
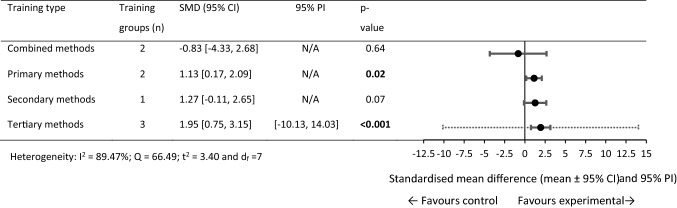
Forest plots showing the SMD (mean [95% CI and 95% PI]) in post-intervention *V*_max_-phase sprint performance between intervention and control athletes. Bold formatting indicates *p* < 0.05. *CI* confidence interval, *N/A* fewer than three training groups available, *PI* prediction interval, *SMD* standardised mean difference, *V*_max_ maximum sprinting velocity

For *V*_max_-phase performance, 31 within-training group effects were analysed from 13 original studies [34, 58, 67, 68, 76, 81, 93, 97, 110–112, 114, 116]. Eight training and control groups from five studies were eligible for a pairwise between-group analysis (sport-only control vs. experimental) [58, 67, 68, 97, 114]. The five available control groups were split in three studies [58, 67, 68] to allow comparison between the multiple training groups in the studies [69]. Figures 6, 7 show the SMD for each training type.

#### Maximum-Velocity Phase Within-Group Changes

The sport-only training, primary, secondary, and combined methods failed to show statistical significance for change in *V*_max_-phase performance. The tertiary training methods showed a significant moderate performance improvement. The tertiary training methods demonstrated a significantly larger training effect than primary training methods.

#### Maximum-Velocity Phase Pairwise Between-Group Differences

The secondary and combined training methods failed to show statistical significance to sprint performance change to sport-only training. Significant performance improvements were observed (large SMD) for the primary and tertiary methods training groups compared with the sport-only control groups. Between-subgroup analysis was not conducted as the tertiary methods were the only training group with more than two training groups available.

### Within-Group Change Design vs. Pairwise Between-Group Effect

No significant difference was observed for the combined methods subgroups (all distance outcomes). Both significant (*V*_max_ phase) and non-significant (0–30 m) differences were found for the primary training between-subgroup analysis. The between-group effect from comparative trials was significantly larger for both tertiary (all distance outcomes) and secondary methods (0–30 m and *V*_max_ phase) (Table 5).

**Table 5:** Subgroup analysis comparing the within-group change standardised mean difference in sprint performance and pairwise between-group effect from comparative trials

Subgroup within study	0–30 m	0 to > 30 m	*V*_max_ phase
Combined methods	*p* = 0.85	*p* = 0.08	*p* = 0.63
Primary methods	*p* = 0.79	NA	↑*p* = 0.02
Secondary methods	↑*p* < 0.01	NA	↑*p* = 0.01
Tertiary methods	↑*p* < 0.001	↑*p* = 0.02	↑*p* = 0.02

↑ indicates that the pairwise between-group effect standardised mean difference was significantly larger (*p* < 0.05) than the within-group change in sprint performance

### Heterogeneity

The degree of overall heterogeneity was high for all outcome measures between studies *I*^2^ (> 75%).

### Sensitivity Analysis

Omitting each study separately identified the effect that each study had on the mean effect. This revealed minor changes only for the secondary training methods. These changes did not have a substantial impact on the statistical significance of the overall mean effect. Sport-only, combined, primary, and tertiary training methods were sensitive to the exclusion of one or more studies independently and, in turn, moderated the statistical interpretation of the results. Removal of one of the five 0 to > 30 m studies [35] from the sport-only methods subgroup moderated the within-group change statistical significance from non-significant (*p* > 0.05) to significant (*p* < 0.05). Removal of one of the five 0–30 m studies [97] and one of two *V*_max_-phase studies from the pairwise between-group differences (sport-only vs. combined training methods) moderated the statistical significance from non-significant (*p* > 0.05) to significant (*p* < 0.05). Removal of one of the four 0 to > 30 m studies [35] from the pairwise between-group differences (sport-only vs. combined training methods) moderated the statistical significance from significant (*p* < 0.05) to non-significant (*p* > 0.05). Removal of two of the five 0–30 m studies [58, 60] and one of three *V*_max_-phase studies [58] from the within-group change primary methods subgroup moderated the statistical significance from non-significant (*p* > 0.05) to significant (*p* < 0.05). Removing one of two 0–30 m *V*_max_-phase primary methods subgroup studies [58] from the pairwise between-group differences (primary vs. combined training methods) moderated the statistical significance from non-significant (*p* > 0.05) to significant (*p* < 0.05). Removing one of the eight within-group 0 to > 30 m studies [89] and one of the six *V*_max_-phase studies [81] from the tertiary training method subgroup moderated the statistical significance from non-significant to significant and from significant to non-significant, respectively.

### Evaluation of Small Study Effects

Inspection of the funnel plots for the within-group change revealed the presence of a statistically significant Egger’s regression intercept, showing evidence of small study effects for the 0–30 m (intercept 9.36; 95% CI 5.68–13.04; *p* < 0.001) and *V*_max_-phase (intercept 11.38; 95% CI − 4.88 to 17.87; *p* < 0.01). For studies included in the pairwise between-group differences comparison, evidence indicated small study effects for the 0–30 m (intercept 8.90; 95% CI 4.22–13.21; *p* < 0.001), 0 to > 30 m (intercept 6.60; 95% CI − 0.10 to 13.27; *p* = 0.05), and *V*_max_-phase (intercept 15.83; 95% CI − 3.15 to 28.14; *p* = 0.02). The SMD between pre- and post-intervention sprint performance was therefore not considered symmetrical, suggesting the presence of significant publication bias [121]. However, there was little evidence to indicate a small study effect for the within-group change in the 0 to > 30 m outcome studies (intercept 3.69; 95% CI − 1.90 to 9.28; *p* = 0.19).

### Moderator Variables

Table 6 presents the subgroup analysis assessing potential moderating factors for sprint performance (0–30 m, 0 to > 30 m performance, and *V*_max_-phase). The between-subgroup analysis showed significant (*p* < 0.05) differences for football code, age, and phase of training; all moderated the overall magnitude of training effects (either smaller or larger SMD). However, the between-subgroup differences were not consistent across distance outcomes. Both playing standard and sex consistently demonstrated no significant difference between subgroups.

**Table 6:** Summary of moderator variable analysis for football code, sex, playing standard, age, and phase of training meta-analysis by subgroup with the sport-only training groups removed

Between-group differences	Subgroup standardised mean difference
*Football code*	
0–30 m Soccer vs. rugby league, *p* = 0.07 Soccer vs. rugby union, *p* = 0.98 Rugby league vs. rugby union, *p* = 0.10 American football^a^ Rugby sevens^a^ 0 to > 30 m American football vs. rugby league, *p* = 0.47 American football vs. rugby sevens, *p* = 0.31 American football vs. rugby union, *p* = 0.08 American football vs. soccer, *p* = 0.34 Rugby league vs. rugby union, *p* = 0.59 Rugby league vs. rugby sevens, *p* = 0.64 Rugby league vs. soccer, *p* = 0.37 Rugby sevens vs. rugby union, *p* = 0.49 Rugby sevens vs. soccer, *p* = 0.02* Rugby union vs. soccer, *p* < 0.001* Australian football^a^ *V*_max_ phase Rugby sevens vs. soccer, *p* = 0.16 Australian football^a^	Soccer 0–30 m (*n* = 62; SMD 0.47; 95% CI 0.34–0.59; 95% PI − 0.55 to 1.48); *p* < 0.001* 0 to > 30 m (*n* = 21; SMD 0.49; 95% CI 0.30–0.68; 95% PI − 0.41 to 1.39); *p* < 0.001* *V*_max_ (*n* = 14; SMD 0.32; 95% CI 0.02–0.62; 95% PI − 0.45 to 1.43); *p* = 0.04*
	Rugby union 0–30 m (*n* = 6; SMD 0.46; 95% CI 0.18–0.74; 95% PI − 0.50 to 1.42); *p* < 0.001* 0 to > 30 m (*n* = 4; SMD 0.07; 95% CI − 0.02 to 0.16; 95% PI − 0.12 to 0.26); *p* = 0.12 *V*_max_ (NA)
	American football 0–30 m (NA) 0 to > 30 m (*n* = 3; SMD 0.33; 95% CI 0.06–0.60; 95% PI − 2.43 to 3.08); *p* = 0.02* *V*_max_ (NA)
	Rugby league 0–30 m (*n* = 4; SMD − 0.06; 95% CI − 0.60 to 0.48; 95% PI − 2.64 to 2.53); *p* = 0.84 0 to > 30 m (*n* = 3; SMD − 0.39; 95% CI − 2.30 to 1.53; 95% PI − 25.17 to 24.39); *p* = 0.69 *V*_max_ (NA)
	Rugby sevens *0–30 m (*n* = 1; SMD 0.43; 95% CI 0.17–0.69); *p* < 0.01* 0 to > 30 m (*n* = 4; SMD 0.15; 95% CI − 0.06 to 0.36; 95% PI − 0.58 to 0.88); *p* = 0.16 *V*_max_ (*n* = 4; SMD 0.08; 95% CI − 0.06 to 0.22; 95% PI − 1.37 to 2.34); *p* = 0.27
	Australian Football 0–30 m (NA) 0 to > 30 m^a^ (*n* = 2; SMD − 0.14; 95% CI − 0.39 to 0.12); *p* = 0.29 *V*_max_^a^ (*n* = 2; SMD 0.09; 95% CI − 0.07 to 0.24); *p* = 0.27
*Sex*	
0–30 m Male vs. female, *p* = 0.15 0 to > 30 m Male vs. female, *p* = 0.77 *V*_max_ phase Male vs. female, *p* = 0.17	Male 0–30 m (*n* = 74; SMD 0.38; 95% CI 0.26–0.49; 95% PI − 0.59 to 1.35); *p* < 0.001* 0 to > 30 m (*n* = 34; SMD 0.30; 95% CI 0.11–0.48; 95% PI − 0.81 to 1.41); *p* < 0.001* *V*_max_ (*n* = 23; SMD 0.22; 95% CI 0.02–0.42; 95% PI − 0.41 to 1.38); *p* = 0.03*
	Female 0–30 m (*n* = 7; SMD 0.64; 95% CI 0.30–0.97; 95% PI − 0.54 to 1.81); *p* < 0.001* 0 to > 30 m (*n* = 3; SMD 0.25; 95% CI 0.00–0.50; 95% PI 2.53–3.03); *p* = 0.05 *V*_max_ (*n* = 3; SMD 0.02; 95% CI − 0.18 to 0.22; 95% PI − 4.99 to 5.96); *p* = 0.84
*Playing standard*	
0–30 m Elite vs. sub-elite, *p* = 0.21 0 to > 30 m Elite vs. sub-elite, NA *V*_max_ phase Elite vs. sub-elite, *p* = 0.55	Elite 0–30 m (*n* = 52; SMD 0.39; 95% CI 0.25–0.53; 95% PI − 0.60 to 1.38); *p* < 0.001* 0 to > 30 m (*n* = 36; SMD 0.28; 95% CI 0.10–0.45; 95% PI − 0.39 to 1.36); *p* < 0.001* *V*_max_ (*n* = 22; SMD 0.21; 95% CI 0.00–0.42; 95% PI …); *p* = 0.04*
	Sub-elite 0–30 m (*n* = 16; SMD 0.58; 95% CI 0.32–0.85; 95% PI − 0.59 to 1.75); *p* < 0.001* 0 to > 30 m (NA) *V*_max_ (*n* = 4; SMD 0.10; 95% CI − 0.18 to 0.22; 95% PI − 1.37 to 2.34); *p* = 0.48
*Age*	
0–30 m Senior vs. youth, *p* = 0.07 0 to > 30 m Senior vs. youth, *p* = 0.24 *V*_max_ phase Senior vs. youth, *p* = 0.37	Senior 0–30 m (*n* = 44; SMD 0.51; 95% CI 0.34–0.68; 95% PI − 0.63 to 1.65); *p* < 0.001* 0 to > 30 m (*n* = 25; SMD 0.19; 95% CI 0.08–0.31; 95% PI − 0.40 to 1.38); *p* < 0.001* *V*_max_ (*n* = 21; SMD 0.25; 95% CI 0.03–0.47; 95% PI − 0.41 to 1.39); *p* = 0.03*
	Youth 0–30 m (*n* = 35; SMD 0.32; 95% CI 0.20–0.44; 95% PI − 0.41 to 1.05); *p* < 0.001* 0 to > 30 m (*n* = 12; SMD 0.48; 95% CI 0.03–0.94; 95% PI − 0.47 to 1.45); *p* = 0.04* *V*_max_ (*n* = 5; SMD 0.00; 95% CI − 0.23 to 0.23; 95% PI − 0.88 to 1.86); *p* = 0.98*
*Phase*	
0–30 m In-season vs. off-season, *p* = 0.91 In-season vs. pre-season, *p* = 0.13 Pre-season vs. off-season, *p* = 0.33 0 to > 30 m In-season vs. off-season, *p* < 0.10 In-season vs. pre-season, *p* < 0.09 Pre-season vs. off-season, *p* = 0.11 *V*_max_ phase In-season vs. pre-seaso,n *p* = 0.36	In-season 0–30 m (*n* = 41; SMD 0.32; 95% CI 0.16–0.48; 95% PI − 0.72 to 1.36); *p* < 0.001* 0 to > 30 m (*n* = 11; SMD 0.64; 95% CI 0.38–0.89; 95% PI − 0.49 to 1.46); *p* < 0.001* *V*_max_ (*n* = 10; SMD 0.28; 95% CI − 0.14 to 0.71; 95% PI − 0.51 to 1.48); *p* = 0.19
	Off-season 0–30 m (*n* = 4; SMD 0.29; 95% CI − 0.13 to 0.71; 95% PI − 1.73 to 2.31); *p* = 0.18* 0 to > 30 m (*n* = 3; SMD 0.33; 95% CI 0.06–0.60; 95% PI − 4.99 to 5.96); *p* = 0.02* *V*_max_ (NA)
	Pre-season 0–30 m (*n* = 26; SMD 0.52; 95% CI 0.31–0.73; 95% PI − 0.58 to 1.62); *p* < 0.001* 0 to > 30 m (*n* = 17; SMD 0.02; 95% CI − 0.23 to 0.26; 95% PI − 0.43 to 1.41); *p* = 0.94 *V*_max_ (*n* = 8; SMD 0.08; 95% CI − 0.04 to 0.19; 95% PI − 0.57 to 1.54); *p* = 0.19

Subgroup analyses showing the SMD (mean; 95% CI and 95% PI) between post and pre-intervention sprint performance outcomes. Some studies were not included because the value used for subgroup analysis was not reported or did not match the appropriate categories. PI were not included for subgroups with fewer than three training groups

*CI* confidence interval, *NA* no training group met the inclusion criteria, *PI* prediction interval, *SMD* standardised mean difference, *V*_max_ maximum velocity-phase sprint performance outcome

^a^Fewer than three training groups

*p < 0.05

## Discussion

### Overview of the Main Findings

Multiple training methods are recommended for improving medium- to long-distance sprint performance because of its importance in the football codes [32, 33, 40–49]. This systematic review with meta-analysis is the first to (1) analyse the impact of different methods in enhancing medium- to long-distance sprint performance outcomes (0–30 m, 0 to > 30 m, and the *V*_max_ phase) within football code athletes and (2) identify how moderator variables (i.e., football code, sex, playing standard, age, and phase of season) affected the training response. This review analysed 60 studies [34–36, 58–68, 75–120], totalling sprint performance measurements from 1500 athletes, thus providing the largest systematic evidence base for enhancing medium- to long-distance sprint performance over distances > 20 m exclusively including football code athletes.

In summary, the meta-analysis of all the included studies showed enhanced sprint performance in the combined, secondary, and tertiary training methods groups. Combined and secondary methods showed small to moderate improvements in 0–30 m and 0 to > 30 m performance. Tertiary methods showed small and moderate performance improvements in both 0–30 m and *V*_max_-phase outcomes, respectively. Significant performance improvements (large SMD) were observed for the combined (0 to > 30 m), primary (*V*_max_ phase), secondary (0–30 m), and tertiary methods (all outcomes) when compared pairwise with the sport-only control groups. These findings support previous literature that stated that the medium to long sprint performance of football code athletes can be enhanced concurrently alongside football code-specific training [25, 41]. Despite several training methods demonstrating significant improvement in sprint performance, it is important to note that the PIs contained both null and negative effects in all training groups. This indicates that, for all training subgroups and assuming a normal distribution of the data, some athletes experienced null or negative performance effects even though the point estimate suggested benefit. Sport-only training showed no significant change in medium to long sprint performance, suggesting such training alone is insufficient to improve performance. The significant differences in between-group effect comparisons for studies with control groups and the within-group change consistently demonstrated a larger effect. Despite sprint measures over > 20 m being a proxy measure of *V*_max_ improvements, changes in performance may not result exclusively from *V*_max_-specific adaptations. Instead, performance changes in outcomes > 20 m may be attributed to either or both adaptations specific to the acceleration or *V*_max_ phases. Between-subgroup analysis identified that football code, age, and phase of training all moderated the overall magnitude of training effects (either smaller or larger SMD). However, the between-subgroup differences were not consistent across distance outcomes. The increase in performance was significantly greater for soccer than for rugby union, rugby sevens, and American football for 0 to > 30 m, whereas the improvement in performance was significantly greater for American football than for rugby union (0 to > 30 m). The increase in performance was significantly greater for youth athletes than for senior athletes (0 to > 30 m). In-season performance changes were significantly greater than in the pre-season and off-season periods in the 0 to > 30 m outcomes only. Playing standard and sex consistently demonstrated no significant difference between subgroups. The lack of consistency may suggest greater importance of other moderator variables, such as training and load prescription (e.g., mode, volume, intensity, and frequency), over the described individual population characteristics.

### Summary of Interventions to Develop Sprint Performance

The 60 studies were categorised into five training modes, resulting in 111 training groups (i.e., sport only, *n* = 27; combined methods, *n* = 35; primary methods, *n* = 8; secondary methods, *n* = 9; tertiary methods, *n* = 59). Of the 60 studies, 26 had sport-only comparator groups [35, 36, 58–68, 75, 82, 88, 90, 92, 95–97, 104, 106, 113, 114, 119], which provided 41 training groups for between-group effect comparisons (combined methods, *n* = 9; primary methods, *n* = 3; secondary methods, *n* = 2; tertiary methods, *n* = 27). No research met the inclusion criteria for the combined specific training methods group, which combined both primary and secondary training methods. These findings highlight the volume of tertiary method training studies and the reported gap in the available literature to support specific sprint-training methods (primary, secondary, and combined specific training methods) in football code athletes [33, 44]. This also further supports the requirement for the within-group analysis, including a greater range of study designs given the small number of studies with a sport-only control group available. The scarcity of specific sprint-training method studies is most probably because football code training typically consists of tertiary training methods to develop the multiple physical capacities (e.g., strength, speed, power) required within these sports. This is a strength of the current study, as previous reviews [32, 33] did not include all training undertaken by the intervention groups within their analysis (e.g., primary or secondary training groups also completing tertiary training methods or vice versa [94, 117, 122, 123]).

The current degree of overall heterogeneity was high for all outcome measures between studies (*I*^2^ > 75% [124]). Heterogeneity is to be expected in systematic reviews given the grouping of both clinically and methodologically diverse studies [124]. The high degree of heterogeneity reflects the diversity of the training effects presented. This is likely due to the wide variation in the intervention characteristics, including training frequency [78, 80], intensity [34, 36, 59, 125], duration [76], volume [109], other training completed [62, 100]), population characteristics (e.g., sex [65], baseline physical characteristics [60, 110], training experience [34, 80]), sprint monitoring methods (e.g., start position, environmental factors [56]), and technology (e.g., equipment [58]). Therefore, these findings should be interpreted carefully as the variation of the effect sizes demonstrates that training response is highly individualised.

The quality of the studies was high (18 ± 1.9; range 11–20) because most studies provided clearly described research methodology, enabling practitioners and/or researchers to replicate or build on research findings reliably [126]. A methodological study scale used to evaluate research conducted in athletic-based training environments [54] showed that, to increase the quality of future studies, researchers should randomise participants, include a control group, and provide a detailed results section. The inclusion of detailed information on additional training conducted in applied settings is important for the understanding of the training intervention undertaken and to fully assess whether any outside interactions with any adaptations were seen following a training intervention [127].

Most training interventions reported positive effects on sprinting capabilities, which suggests that sprint performance outcomes can easily be improved with a variety of methods. However, this needs to be considered from the context of the literature base and the relative importance of phase-specific adaptations. Included studies represented both youth and senior athletes from elite and sub-elite cohorts, with the majority having limited previous systematic exposure to the intervention methods [58, 80, 82, 85, 89, 95, 114]. Based on the dose–response relationship and the principle of diminishing returns, athletes with a relatively low training age are more likely to have greater training responses [128–130]. However, as previously reported [33], this does not appear to be the case for the *V*_max_ phase or highly trained populations. Highly trained athletes have demonstrated that mean annual within-athlete sprint performance differences are lower than typical variations, or smallest worthwhile change, and the influence of external conditions (e.g., wind, temperature, altitude, timing methods/procedures [56, 130]). Inspection of the funnel plot and Egger’s regression intercept identified evidence of small study effects in the 0–30 m and *V*_max_-phase performance outcomes. The SMDs between pre- and post-intervention sprint outcomes were not considered symmetrical, suggesting the presence of significant publication bias. While publication bias towards studies reporting positive outcomes may be involved, another plausible explanation is the lack of a control group in many studies, as the results might have been affected by learning effects or the football code training in the intervention period.

### Subgroup Analyses of Training Methods

The principle of specificity [137, 138] was used to categorise the training intervention subgroups (i.e., sport only, primary, secondary, tertiary, and combined). Primary methods present the greatest specificity by simulating the sprint movement pattern [131], whereas the secondary methods are less specific, involving overloaded sprinting actions. The tertiary training methods included strength, power, and plyometric training, which are considered the least ‘specific’ to sprint performance as these methods are commonly performed to target neuromuscular adaptations rather than simulating movement mechanics [132]. The extent to which the method impacts on and ‘transfers’ to sprint performance ultimately determines the quality of a training programme to improve athletic performance [133].

The factors underpinning the development of sprint performance appear to be consistent across sports [134–140]. Practitioners can target the determinants of performance, such as optimising the sequencing of stride length and frequency, enhancing the athlete’s physical capacities relative to BM (e.g., lower limb force–velocity–power; stiffness) and increasing the mechanical effectiveness of force application [134, 136, 138, 140–145]. These methods provide practitioners with multiple methods of developing sprinting performance [130, 144, 146]. Performance improvements result from specific transferable training adaptations typically categorised as neural or morphological (architectural or structural) factors [26, 146–149]. However, training effects appear to be mode specific, with distance-specific performance changes (e.g., 0–30 and 0 to > 30 m) associated with phase-specific adaptations [32]. Although the factors underpinning sprint development are consistent, phase-specific differences in both kinetic and kinematic variables are clear [26]. The importance of mechanical variables appears to shift as sprint distance increases (e.g., greater association between theoretical maximal force generation in shorter sprints vs. greater associations in maximum theoretical velocity force can be applied in longer sprints [150]). Therefore, it is important to consider the phase-specific adaptations that may be present across medium- to long-distance sprint outcomes.

Despite researchers and practitioners typically using outcome measures over distances > 20 m as a proxy measure of *V*_max_-phase capabilities, performance changes may be attributed to either or both adaptations specific to the acceleration or *V*_max_ phases, not the *V*_max_ phase exclusively. This is evident as the *V*_max_ phase presented performance changes that were distinctly different from both the 0–30 m and the 0 to > 30 m outcomes. Although the acceleration and *V*_max_ phases are related [8, 132, 150–153], separate physical capacities and mechanical parameters determine sprint performance [27, 29, 129, 137, 140, 154–156]. Research has demonstrated that football code athletes can attain *V*_max_-phase sprinting patterns at distances ≤ 20 m [6–10, 29]. Therefore, after 20 m, there is likely an increasing influence of the *V*_max_ phase, with the time spent increasing with distance. Therefore, given the sequential phases of sprinting, both 0–30 and 0 to > 30 m outcomes will be influenced by changes in acceleration, with the 0 to > 30 m outcome influenced to a lesser extent (more time performing *V*_max_ sprinting patterns), whereas the *V*_max_-phase flying sprint split times and *V*_max_ assessments do not include, or include a limited, acceleration phase. Hence, it is important to emphasize that, although the sequential phases are related, different factors affect performance in each phase. Therefore, training protocols to develop each of these phases must also differ [33]. This was evident in both the secondary and the combined methods training groups. Hence, when including all studies, both training methods presented a significant improvement in both 0–30 and 0 to > 30 m performance, whereas they produced non-significant trivial changes in *V*_max_-phase performance. Therefore, practitioners should also consider the mechanical and neuromuscular requirements that shift across the sub-phases (acceleration, maximal speed, and maintenance) of medium- to long-distance sprint outcomes and the implications of these for training phase-specific performance [26, 150, 154, 157].

#### Sport-Only Training

Sport-only training focuses on the development of technical and tactical performance within football and does not include any specific or non-specific sprint training. The meta-analysis showed that sport-only training groups did not significantly change sprint performance [35, 36, 58–68, 75, 82, 88, 90, 95–97, 104, 106, 113, 114, 119]. Football code training is characterised by multidirectional and intermittent bouts of high-intensity running and sprinting interspersed with bouts of moderate- and low-intensity activity (e.g., jogging, walking, and repositioning [158–161]). Therefore, although football code training may involve athletes repeatedly performing short sprints (e.g., 5–20 m, 2–3 s) during and between sport-specific actions [2, 23, 158, 159, 162], this most likely has limited or no very-high-speed or sprint threshold running [160, 161, 163]. Such training methods do not meet the recommendations that athletes be exposed to multiple sprints where they maximally accelerate to achieve and maintain *V*_max_ with complete recovery between efforts to effectively enhance sprint performance [130]. Further explanations could include residual fatigue and the interference effect affecting maximal force and velocity outcomes within sport-only practices [130, 164–166]. Therefore, evidence suggests that sport-only training alone is insufficient to improve medium to long sprint performance, and football code practitioners should consider this within their planning and delivery of training.

#### Primary Methods

Primary methods simulate the sprint movement pattern (e.g., sprint-technique drills, stride length and frequency exercises, and sprints of varying distances and intensities). The combined exposure of large forces (> 2 × BM) produced over short ground-contact periods (~ 0.08 to ~ 0.20 s) performed at high movement velocities (7–10 m·s^−1^) while maximally sprinting results in both a coordinative overload and high neuromuscular stimulation [134, 136–138, 140, 155, 167]. Therefore, exposure to maximal sprinting is expected to facilitate chronic physical adaptations and enhanced mechanical efficiency to improve sprint performance [133, 134, 136–138, 140, 167]. However, no studies have measured chronic kinematic changes over distances > 20 m in response to primary training methods (no additional tertiary methods) to support their use in football code athletes [67, 101]. Our findings suggest that primary training methods [58, 60, 67, 86, 101, 102, 116] may not significantly improve sprint performance and—in some cases—may impair performance. The primary methods within-group changes presented no significant change in sprint performance (i.e., 0–30 m, SMD 0.20 [95% CI − 0.01 to 0.42; 95% PI − 0.51 to 0.92]; 0 to > 30 m, SMD 0.06 [95% CI − 0.14 to 0.25; 95% PI not applicable as *n* < 3]; *V*_max_ SMD − 0.07 [95% CI − 0.24 to 0.10; 95% PI − 1.75 to 1.61]). This was further supported by the pairwise between-group comparisons (sport only vs. primary), which confirmed no significant difference was evident in the 0–30 m: SMD 0.33 (95% CI − 0.58 to 1.25; 95% PI not applicable as *n* < 3). Despite the *V*_max_-phase outcome reporting, the primary methods were superior (large SMD) to sport-only training (SMD 1.13 [95% CI 0.17–2.09; 95% PI not applicable as *n* < 3]), and this difference reflects the maintenance of sprint performance rather than the reduced performances reported in the sport-only groups [58, 67]. The contradictions between our findings and previous reviews supporting primary training methods is likely because other studies misclassified training methods by not including additional training (e.g., resistance training), most probably as part of their usual training programme [38, 117, 168–171]. Therefore, previous review findings may support a combined approach of both specific and non-specific training, not primary training alone [32, 33].

Football code athletes have high chronic exposure to short sprints (< 20 m) with incomplete recovery between sprints as part of the demands of training and matches; therefore, replicating these exposures is unlikely sufficient stimulus for neurological or morphological adaptations [158–161, 172]. Prescribing short-sprint repetition distances (e.g., 18.7–20 m [58, 60, 116]) limits athlete exposure to sprinting at *V*_max_ (typically achieved at > 20 m in football code athletes [8, 9, 27, 29–31]), performed at submaximal efforts (< 95% *V*_max_ [102]) and/or with incomplete recovery (e.g., 2–3 min between repetitions [< 1–2 min of activity^−1^]) for medium to long sprints (e.g., 30–55 m sprints, ~ 4–7 s duration [67, 86, 102]). Furthermore, incomplete rest between sprint efforts may reduce maximal sprint intensity, causing metabolic stress and reduction in energy substrates [173–175]. However, it is worth noting that the removal of two studies [58, 60] that prescribed short sprints moderated the statistical significance for the 0–30 m and *V*_max_-phase outcomes from non-significant to significant. These findings contrast with the findings in short-sprint performance, indicating that longer sprints and *V*_max_-phase outcomes may be more susceptible to performance changes from primary training methods when prescribed appropriately [25]. Future studies should provide complete rest periods between maximal intensity sprints reaching and maintaining *V*_max_.

Running technique drills that simulate the sprinting action by isolating specific movements into more manageable components [130, 176] are a component of primary training. For positive reinforcement of the technique, sprinting biomechanics must closely resemble the action and develop the athlete’s limiting factor(s) [131, 177]. However, technique drills (e.g., A and B drills) are often performed at much slower velocities than sprinting, potentially not replicating sprinting from a kinematic standpoint [178]. It has been questioned whether running drills have value, especially when performed incorrectly [179, 180]. However, as with short-distance sprint outcomes [25], no study has evaluated the effects of including/excluding sprint-technique drills in football code athletes, and explanations of the training prescription are often limited. Therefore, sprint training that addresses the magnitude and rate of force production on the ground and the mechanical efficiency (e.g., tertiary or secondary methods) may be more appropriate [180].

#### Secondary Methods

Secondary training modalities apply overload to the sprinting action by reducing (e.g., resisted sprinting) or increasing (e.g., assisted sprinting) the movement speed, allowing athletes to reach supramaximal velocities. Across the seven studies [58, 60, 79, 85, 86, 101, 116], findings showed a significant moderate within-group improvement in 0–30 m (SMD 0.61 [95% CI 0.31–0.91; 95% PI − 0.43 to 1.65]) and small improvements in 0 to > 30 m (SMD 0.37 [95% CI 0.25–0.50; 95% PI: all studies shared a common effect size]), with no significant changes in *V*_max_ phase (SMD 0.07 [95% CI − 0.10 to 0.23; 95% PI − 1.57 to 1.71]). These findings are supported by the pairwise between-group analysis (sport only vs. control), confirming the effectiveness of the secondary methods (large SMD) in enhancing or maintaining medium to long sprint performance, respectively, compared with reductions in sport-only training groups (0–30 m, SMD 2.78 [95% CI 1.53–4.03; 95% PI not applicable as *n* < 3]) and *V*_max_ phase (SMD 1.27 [95% CI − 0.11 to 2.65; 95% PI not applicable as *n* < 3]). Training adaptations have been reported as being velocity change specific (%*V*_max_ increase vs. reduction [181]), with variations in distance-specific improvements for secondary methods (i.e., assisted vs. resisted) [116]. This is evident in both our findings and those of another review, reporting no significant improvements in *V*_max_-phase outcomes in secondary training methods [33]. Hence, the improvements in 0–30 and 0 to > 30 m performance may be a result of acceleration-specific adaptations reflected in short-sprint improvements included in the sprint outcome. The overload of the secondary training methods results in neurological or morphological adaptations, allowing greater generation of ground reaction forces and improved mechanical efficiency to enhance performance [33, 44].

Resisted sprints (i.e., loaded sleds) were shown to increase both stride length and frequency and lead to an acute increase in forward trunk lean (improved position to generate horizontal impulse) during sprints < 20 m in team sport athletes and university students [182–185]. In contrast, assisted methods demonstrated increased stride length and decreased stride frequency in track athletes [33, 44], whereas reduced ground contact times were reported in football code athletes [101]. Studies measuring chronic temporospatial changes in response to secondary training methods (no additional tertiary methods) to support these in football code athletes are currently limited [101]. Of the two overload strategies, resisted sprint training [58, 60, 79, 85, 86, 116] has received the greatest attention in the research on football code athletes despite significant improvements in both training methods (resisted [58, 60, 85, 86, 116], assisted [116], and a combination of both [101]). Currently, no study has reported a statistically superior training effect between assisted and resisted training modes, so which training mode is the most effective for developing sprint performance remains unclear. Therefore, secondary training methods appear to be an effective method for coaches and athletes to improve 0–30 and 0 to > 30 m sprint performance outcomes. However, if the aim is to develop the *V*_max_-phase performance, then training strategies other than sled towing (e.g., weighted vests) may be needed to develop phase-specific adaptations. For example, vertical forces have a greater relative contribution to the *V*_max_ phase [136, 137]. Acute kinematic differences suggest vertical force production when sprinting could be developed by undertaking training strategies utilising weighted vests by providing a greater load on the eccentric braking phase at the beginning of the stance phase [185, 186], whereas sled towing is expected to provide a superior adaptation in horizontal force production [185, 187, 188]. Further research is required to determine the optimal load, loading strategy, and dose for performance enhancement, particularly for *V*_max_ development.

#### Tertiary Methods

Tertiary training methods represent a wide range of training methods (e.g. strength, power, plyometrics [32, 189]) that are commonly performed to target neuromuscular adaptations that determine sprint performance (e.g., force–velocity–power and force–velocity profile) rather than simulating movement mechanics [26, 130, 146, 150]. Using the load–velocity relationship, the appropriate resistance (bodyweight or external loads) limits either the maximum velocity or the force at which the maximum effort will occur, or both [190]. Therefore, practitioners are able to use force–velocity–power-orientated exercises in isolation or in combination (e.g., high force/low velocity vs. low force/high velocity vs. peak power load) to target load-specific adaptations [26, 130, 146, 150].

Despite previous criticisms of tertiary training methods questioning the effectiveness of developing sprint performance, significant within-group moderate improvements were found for the 0–30 m (SMD 0.38 [95% CI 0.23–0.53; 95% PI − 0.63 to 1.41]) and *V*_max_-phase outcomes (SMD 0.45 [95% CI 0.08–0.81; 95% PI − 0.97 to 1.83]). No significant change was found for the 0 to > 30 m outcome when all studies were included (SMD 0.22 [95% CI − 0.26 to 0.70; 95% PI − 1.73 to 2.17]). The significant within-group changes in point estimate in the 0–30 m and *V*_max_ outcomes were supported by significant findings in the pairwise between-group analysis (sport only vs. tertiary), with observed performance improvements (large SMD) confirming the effectiveness of the tertiary training methods in enhancing medium to long sprint performance compared with sport-only training: 0–30 m (SMD 1.49 [95% CI 0.95–2.03; 95% PI − 1.06 to 4.03]), 0 to > 30 m (SMD 1.12 [95% CI 0.56–1.68; 95% PI − 0.29 to 2.52]), and *V*_max_ phase (SMD 1.95 [95% CI − 0.75 to 3.15; 95% PI − 10.13 to 14.03]). Therefore, phase-specific adaptations may be present. However, the presence of significant improvements in both 0–30 m (likely a greater influence of the acceleration phase) and the *V*_max_-phase performance changes are likely a result of both acceleration- and *V*_max_-phase-specific adaptations. Research comparing the kinetic factors underlying differences between athletes with higher *V*_max_ capabilities (sprinters) and slower athletes (soccer players), found that, at the same touchdown velocity, the sprinters attenuated the eccentric forces to a greater extent in the late braking phase and produced a higher antero‐posterior component of force, yet ground contact durations were similar across groups [27]. Therefore, training methods such as strength, power, or plyometrics training that increase an athlete’s ability to produce sufficient vertical force, to withstand and reverse eccentric braking forces, and to generate high antero‐posterior propulsive force may be required to enhance an athlete’s ability to accelerate more rapidly while also attaining a greater *V*_max_ [27, 130]. The improved physical capacities developed during tertiary training methods have previously been shown to manifest in significant improvements in sprint performance with associated reductions in contact time or changes in stride frequency and length [34, 67, 169, 170]. Therefore, correspondence between the larger ground reaction forces produced across medium- to long-distance sprints and the neural and morphological adaptations induced by these training methods is likely to be high [140]. Hence, the lack of significance in the 0–30 m outcomes is likely due to large significant reductions in sprint performance as presented by Gabbett et al. [89], moderating the statistical interpretation of the results and therefore supporting previous research [32] for the use of tertiary training methods (i.e., strength, power, and plyometric training) performed individually or in combination (e.g., strength power and plyometrics training) for improving sprint performance.

Considerations should be made when training for increased mass development, which is often associated with tertiary methods: as an athlete gets heavier they may not produce higher maximal force characteristics when normalised for BM [132]. Therefore, the force requirements in the stance leg increase with BM to minimise the braking forces and maximise propulsive forces to attain *V*_max_, as does the aerodynamic drag resulting from a larger frontal surface area [132, 191]. Hence, increases in BM may be counterproductive for sprinting, at least when not moving an external mass [132].

#### Combined Methods

Combined methods training includes both specific sprint training (primary and or secondary methods) and tertiary methods, recommended by researchers and sprint and football code practitioners to develop sprint performance [24, 32, 133, 192–194]. This combination of both training methods enables practitioners to provide stimuli to develop both mechanical efficiency and the maximal physical capabilities of the lower limb concurrently [110, 169, 170, 195]. Previous studies of combined specific and tertiary training methods demonstrated significant improvements in physical capacities (e.g., force, velocity, and power [36, 110]), increased stride lengths, reduced stride frequencies, and reduced stance contact times [76, 169, 170]. However, the changes in spatiotemporal variables are limited to short distances, with no significant changes presented in medium-distance sprints (e.g., stride length or frequency and contact or flight times [36, 76]). This review found significant within-group moderate improvement at 0–30 m (SMD 0.43 [95% CI 0.21–0.65; 95% PI − 0.69 to 1.55]) and small improvements in 0 to > 30 m (SMD 0.33 [95% CI 0.15–0.51; 95% PI − 0.50 to 1.16]), with no significant change in the *V*_max_ phase (SMD 0.05 [95% CI − 0.23 to 0.34; 95% PI − 1.01 to 1.11]). Pairwise within-group analysis (sport only vs. combined) indicated significant performance improvements in favour of combined methods (large SMD): 0 to > 30 m, SMD 1.51 [95% CI 0.21–2.80; 95% PI − 4.26 to 7.28]). Interestingly, the 0–30 m and *V*_max_-phase contrasted with these findings, suggesting the combined methods were no more effective than sport-only training: 0–30 m (SMD 0.58 [95% CI − 0.93 to 2.10; 95% PI − 5.20 to 6.37]); *V*_max_ phase (SMD − 0.83 [95% CI − 4.33 to 2.68; 95% PI not applicable as *n* < 3]). Sensitivity analysis appeared to indicate that the single study demonstrating a large reduction in the *V*_max_-phase sprint performance changed both the statistical significance and the direction of the reported effect [97]. The negative effects reported in this study were attributed to the interference of the volume of aerobic training and thus is an important consideration when attempting to develop medium to long sprint performance. As discussed in Sect. 4.3.3, phase-specific adaptations appear to be present, with performance changes likely a result of acceleration-specific adaptations reflected in short-sprint improvements included in the sprint outcome. Despite presenting significant training effects, each method presented different training methods (see Table S3 in the ESM). Therefore, combined specific methods appear to be an effective training method for football code athletes for 0–30 and 0 to > 30 m sprint performance outcomes. However, if the aim is to develop the *V*_max_-phase performance, training strategies may be modified to develop phase-specific adaptations (e.g., increase vertical ground reaction in reduced stance phases). Further research is required to identify the optimal combination of exercises and training loads to improve phase-specific performance.

### Moderator Variables

It is important to identify the moderator variables (i.e., football code, sex, age, playing standard, stage of the season) that may impact upon sprint training outcomes. Studies were excluded from the analysis if the value used for subgroup analysis was not reported, if they did not provide sufficient detail, or if they did not match the appropriate moderator categories.

#### Sex

The meta-analysis of the intervention training groups found that the sprint performance of both male and female football code athletes could be improved. However, the improvements for the 0 to > 30 m and *V*_max_-phase outcomes in females were not significant. When comparing male and female athletes, there was no significant difference between the training effects. This should be taken within the context of the scarcity of the available information on female athlete training compared with that for males [196]. The limited research comparing sex differences in training response in football code athletes found no significant effect of sex on changes in sprint performance [65]. Therefore, despite the demonstrated differences between physical characteristics [21, 132] and endocrine response [197] to training between males and females, evidence is currently insufficient to suggest that practitioners should approach developing sprint performance differently based on an athlete’s sex.

#### Playing Standard

Both elite and sub-elite subgroups improved sprint performance. However, there was no significant improvement in sub-elite *V*_max_-phase performance. The between-group comparison identified no significant difference between the training effects for elite and sub-elite groups. Despite sprint performance differentiating between performance standards [19–21], no study has explored whether sub-elite athletes are more sensitive to training than elite populations. However, research has demonstrated a decrement in the magnitude of the correlations with increasing levels of practice between the lower limb neuromuscular maximal capabilities and the ability to generate force during sprinting for sub-elite athletes compared with elite athletes [129, 150]. Therefore, further improvements may be represented by the ability to effectively apply force into the ground at progressively increasing velocities (mechanical effectiveness) to achieve either a greater rate of acceleration or enhanced *V*_max_ performance, or both. Hence, for medium- to long-distance sprints, a greater focus on developing the mechanical capabilities contributing to the athlete’ s ability to generate propulsive impulse (force × time) and their application at higher velocities and decreasing ground contact times (i.e., mechanical efficiency, theoretical maximal horizontal velocity and maximal power) is required [146, 150]. Theoretically, this may be achieved by using resisted sprints that enable athletes to apply force at high velocities (low loads or assisted sprinting), by training at loads that correspond with optimal load for maximal power as well as low load (BM) or assisted horizontal or vertical jumps [146]. However, further research is required to demonstrate the effectiveness of these training strategies. Therefore, despite the demonstrated differences between physical characteristics between elite and sub-elite athletes [132] when considered independent of training status, evidence is insufficient to suggest that practitioners should approach developing sprint performance differently based on athlete’s playing standard within the football codes.

#### Age

The sprint performance of both senior and youth cohort subgroups was enhanced following training interventions, apart from the youth *V*_max_ phase. Between-group comparisons identified that youth athletes enhanced sprint performance more than senior athletes at 0 to > 30 m, which supports research stating that training response is typically greater in younger athletes than in their older counterparts [89]. Factors such as chronological age may have moderated the training effects of the tertiary training methods in male youth athletes, with a greater training effect in younger (< 15 years) than in older (< 18 years) athletes [89]. Youth athletes experience multiple morphological and neural changes as a result of growth and maturation [198], which has implications for sprinting performance changes [48, 50, 199]. The stage of maturation has been shown to moderate the training effect, with youth athletes training at pre-peak height velocity presenting lesser improvements than those at mid- and post-peak height velocity [48, 50]. Changes in youth cohorts may have been affected by the inclusion of pre-pubescent athletes and ineffective training exposures [93], which was not considered in the current analysis. These training effects suggest that coaches of youth athletes should take into consideration chronological and maturational age, increased baseline performance levels, and greater training experience [89, 200]. However, further research is required to understand sprint performance outcomes by age, which could include maturity grouping.

#### Sport

All sprint performance outcomes were improved in the soccer subgroup. Rugby union and American football presented significant improvements in 0–30 and 0 to > 30 m, respectively. No significant improvement was found in rugby league, rugby sevens, or Australian Rules football. Football codes training subgroups with limited representation in the literature (one to two training groups for a given distance outcome) were not considered for subgroup analysis (e.g., 0–30 m rugby sevens [*n* = 1] [87]). Despite differences in physical characteristics [129, 132] and movement demands [158, 159], there were limited between-subgroup differences. The between-group comparison showed that the increase in performance was significantly greater in soccer than in rugby union, rugby sevens, and American football (0 to > 30 m). The improvement in performance was significantly greater in American football than in rugby union (0 to > 30 m). No significant differences were found between the training effects for the football code subgroups for the *V*_max_-phase outcome. Although several factors may have contributed to the significant differences (e.g., training content, duration, frequency), the greater training experience in various forms of resistance training in the rugby codes and American football (e.g., ≥ 2 years’ systematic resistance training [76, 83, 105, 117, 118, 201]) may have resulted in lower morphological or neurological adaptability to the training stressors, resulting in lower training responses compared with the less training-experienced soccer subgroups [130, 132]. However, the literature is insufficient to demonstrate the between-subgroup differences across all sprint performance outcomes, and it remains unclear whether these are specific to training methods or distance outcomes. No study has compared the difference in training effects between football codes implementing matched training interventions in football code athletes on sprint performance. Therefore, evidence is insufficient to support coaches adapting sprint-training methods based on football code.

#### Season

The in-season and off-season subgroups presented significant improvements in 0–30 and 0 to > 30 m, despite practitioners typically having less time available to develop physical or movement capacities during the in-season and off-season periods [51]. Pre-season subgroups only significantly enhanced 0–30 m performance, and no significant improvement in the *V*_max_ phase was observed at any phase of the season. It is generally reported that fitness improvements are observed in the pre-season, with subsequent stabilisation of such fitness variables in-season [202]. Consequently, greater benefits are expected in trials performed during the pre-season period than in those in the in-season [203, 204]. The between-group comparison found significantly greater improvements in-season compared with pre-season and off-season in the 0 to > 30 m outcome only. Therefore, with appropriate prescribed training methods, 0–30 and 0 to > 30 m sprint performance can be enhanced in-season. The 0 to > 30 m pre-season subgroup was sensitive to the large reduction in training performance presented by Gabbett et al. [89], explaining the lack of significant improvement. The *V*_max_ phase appeared to present greater resistance to change based on the current training programmes. None of the included studies compared the difference between training effects between the phase of the season, implementing matched training interventions in football code athletes on sprint performance. Therefore, despite the differences in training demands between training phases, evidence is insufficient to support coaches adapting sprint-training methods based on the phase of the season.

### Limitations

Whilst this work represents the largest systematic review and meta-analysis of medium- to long-distance sprint performance, limitations do exist. First, this review classified training into groups (i.e., sport-only, primary, secondary, tertiary, combined, and combined specific methods), which improved on previous classifications [32, 39] but also did not consider the complexity of sprint performance development within the training prescription, the population, and the assessment methodologies. The broad within-group change approach taken was used to review all available literature; however, this method represents a suboptimal method of exploring training causality while also providing additional areas of bias to the interpretation (e.g., regression to the mean [205]). However, we attempted to address this by combining a within-group pre-post change design and a pairwise between-group design, enabling an evaluation of both high-quality controlled trial comparisons and an exploration of the breadth of the available literature using a range of research designs. Despite the important influence of prior training status and physical capacities [128–130], it was not possible to include these as moderators for several reasons: (1) most studies did not report physical capacity and/or training experience within their descriptive statistics; (2) those that did were inconsistent in how they were reported and the testing methods used; and (3) studies were often limited to years of football code-specific training or resistance training, with little consideration of how the stimulus was provided. Therefore, the level of detail to fully understand sprint development is lacking, but this is difficult in the context of understanding sprint development and the multiple factors that interact. However, the review attempted to analyse several moderator variables (i.e., football code, sex, playing standard, age, and phase of the season), highlighting a limitation that most research is undertaken using parallel-group trials within male soccer athletes involving mainly tertiary training methods. Therefore, research including randomised controlled trials across the football codes and female cohorts using multiple training methods is limited, which may affect the meta-analysis and moderator variable analysis and subsequent interpretation. Despite these limitations, the information gathered from the current review with meta-analysis may support practitioners in making evidence-informed decisions when organising and evaluating training.

### Future Research Directions

This review presented similar research directions to those presented in the short-sprint training literature as the limitations were consistent across all outcomes [25]. Where possible, future research should use high-quality research designs (e.g., randomised controlled trials) to expand and reaffirm the current findings whilst addressing the multiple gaps in specific populations. Research is required to examine the training effects in football codes other than soccer (e.g., rugby codes, American football, Australian Rules, Gaelic football, and futsal), in world-class and successful elite athletes, in trained populations with systematic training exposures, in youth athletes of various growth and maturational status, and in female athlete cohorts. It is worth highlighting that, although several effective training methods are reported, it may be inappropriate to try to define the best methods for enhancing sprint performance in football (e.g., exercises, set and repetition schemes). Instead, the integration of different methods based on the training background, individual requirements, and progression over the training process needs to be further analysed to inform the optimal stimulus and organisation of training. It is essential that future research designs include pairing subjects based on resistance training experience and/or physical capacities (i.e., lower limb force characteristics) to establish a greater understanding of whether training changes and adaptations are dependent upon these variables. Both researchers and practitioners should consider the combined modelling of velocity–time curves with kinematic and kinetic changes assessed at more frequent intervals. This would enable practitioners to isolate and confirm a time course of adaptations and the underlying causes of changes in performance [21, 129, 150] whilst also reducing the limitations associated with pre- and post-sprint times or velocities [55]. Given the respective importance of repeated sprint ability and non-linear sprint outcomes in the football codes, future research should explore their development, providing practitioners with a more comprehensive overview of developing athletes’ sprint characteristics.

Research identified that the majority of elite sprint and football code coaches reported utilising and advocating for an integrated approach using the combined training methods approach [24, 32, 133, 192–194]. This is performed both individually and in separate sessions and combinations (e.g., complex or contrast sets), enabling the development of multiple physical capacities and skills simultaneously [24, 32, 133, 192–194]. Therefore, further research would be better suited to manipulating the combination, sequencing, and loading parameters of combined specific and non-specific methods to enhance sprint performance longitudinally as, ultimately within the football codes, combined training is implemented. This should be combined with methods of profiling that allow optimisation and individualisation of training exposures [150, 189, 206–208], which may reduce the variability in performance change [189]. While exercise specificity is certainly an important principle when developing a training programme, it is only one of several principles that will influence the effectiveness of the programme. Therefore, future research should continue to explore within and between subgroups the effects of overload, variation, and reversibility and the effect on sprint performance change [26]. Furthermore, this needs to be supported with determining the minimal and optimal training doses to retain and develop sprint performance in football code athletes. This will directly influence practitioners’ organisation of training and the prescribed loading variables.

## Conclusions

Establishing the most effective methods to improve medium- to long-distance performance outcomes is an important consideration for practitioners working across the football codes. This work represents the first systematic review and meta-analysis of sprint performance development using medium- to long-distance outcomes that include all training modalities while exclusively assessing within- and across-football code athletes. The results indicate that medium to long sprint performance outcomes can be enhanced through secondary (i.e., resisted or assisted sprinting), combined (i.e., primary or secondary and tertiary training methods) (0–30 m and 0 to > 30 m), and tertiary training methods (0–30 m). In addition, tertiary training methods were the only method that enhanced *V*_max_-phase performance significantly. Performance changes in outcomes > 20 m may be attributed to either or both adaptations specific to the acceleration or *V*_max_ phases, and not *V*_max_ exclusively. Despite this, when comparing training typology, no individual mode was found to be the most effective. However, both sport-only training and primary training methods appeared to be insufficient to develop medium- to long-distance sprint performance outcomes. The null and negative performance effects present in all training group PIs warrant caution, as—regardless of training mode-specific point estimate—factors such as athlete’s capacities, previous training exposures, and the programme design may moderate positive performance adaptations. Moderator effects, although not mode specific, suggested that there is no consistent effect of age, sex, playing standard, and phase of the season on sprint performance change across outcomes. Regardless of the population characteristics, medium- to long-distance sprint performance can be enhanced by increasing either the magnitude or the orientation of force an athlete can generate and express in the sprinting action, or both. These findings present practitioners with several options to suit their programme to enhance medium- to long-distance sprint performance.

## Supplementary Information

Below is the link to the electronic supplementary material.

Supplementary file1 (DOCX 59 kb)

Supplementary file2 (DOCX 58 kb)

Supplementary file3 (DOCX 34 kb)

## Declarations

### Availability of data and materials

The datasets generated during and/or analysed during the current study are available from the corresponding author on reasonable request.
