# Supplementary material for: The Training of Medium- to Long-Distance Sprint Performance in Football Code Athletes: A Systematic Review and Meta-analysis
Source: Sports Med. 2021 Sep 9;52(2):257–86. doi: 10.1007/s40279-021-01552-4 (PMC8803780; doi:10.1007/s40279-021-01552-4)
Supplement: Supplementary file 1 — Supplementary file1 (DOCX 59 kb) [file 40279_2021_1552_MOESM1_ESM.docx]

**Electronic Supplementary Material Table S1**

Article title - The Training of Medium-Long-Sprint Performance in Football Code Athletes: A Systematic Review and Meta-Analysis

Journal name – Sports Medicine

Author names - Ben Nicholson, Alex Dinsdale, Ben Jones, and Kevin Till.

Affiliations - Leeds Beckett University, Carnegie Applied Rugby Research (CARR) centre, Carnegie School of Sport, Leeds, United Kingdom. Yorkshire Carnegie Rugby Union club, Leeds, United Kingdom. Leeds Rhinos Rugby League club, Leeds, United Kingdom. England Performance Unit, The Rugby Football League, Leeds, United Kingdom. School of Science and Technology, University of New England, Armidale, NSW, Australia. Division of Exercise Science and Sports Medicine, Department of Human Biology, Faculty of Health Sciences, the University of Cape Town and the Sports Science Institute of South Africa, Cape Town, South Africa.

corresponding author e-mail address – b.t.nicholson@leedsbeckett.ac.uk

**Table S1**

**Characteristics of the non-specific/tertiary sprint training groups included in the review**

| **Study (year)** | **Subjects** | **Training type and organisation** | **Training methods** | **Other training and testing equipment** | **Mean difference, 95% CI, percentage change, Std. Mean Difference IV, Random, 95% CI, weight, Qualitative inference** |
| --- | --- | --- | --- | --- | --- |
| Alptekin et al. (2013) A (1) | M, n=12, Soccer Players; Age 13.71±0.53 years | Plyometrics training 2d/wk, 8wks, 16 sessions, Phase not reported | High intensity low volume (2 sets of 10-20 reps/set) plyometrics (50cm hurdle jumps) + low load (BW, BW + 1kg med ball) low volume (2 sets of 20 reps/set) UB + core strength training (press ups, chest passes and weighted sit ups @1kg med ball) | Soccer training 2 times/wk.  Newtest 1000 | 0-30m performance = MD (s): -0.02; 95% CI [-0.09, 0.05]; % Change -0.42%;  SMD: -0.06; 95% CI [-0.29, 0.16] Weight 2.26%; Inference - Trivial |
| Borges et al. (2016) B (2) | M, n=11, Elite Soccer Players; Age 16±0.6 years | Plyometrics training 1-2d/wk, 7wks, 12 sessions, In-season | 2-7 sets of (10 foot contacts/set) high-very high intensity plyometrics (bi-lateral and unilateral box jumps and drop jumps) | 90-150 minutes soccer training session every morning, 5 times/wk, and one official match every Saturday  Photocell system | 0-30m performance = MD (s): -0.03; 95% CI [-0.11, 0.05]; % Change -0.7%;  SMD: -0.08; 95% CI [-0.32, 0.15] Weight 2.25%; Inference - Trivial |
| Bouguezzi et al. (2018) A (3) | M, n=15, Elite Soccer Players; Age 11.32±0.27 years | Plyometrics training 1d/wk, 8wks, 16 sessions, In-season | Low volume (3-6 sets of 8-14 reps/set) moderate intensity plyometric training of unilateral and bilateral acyclical exercises (CMJ's, ankle Hops, zig-zags) | 3-4 soccer training sessions/wk  Electronic timing system (Microgate SARL, Bolzano, Italy) | 0-30m performance = MD (s): 0.06; 95% CI [-0.03, 0.15]; % Change 1.12%;  SMD: 0.14; 95% CI [-0.06, 0.34] Weight 2.28%; Inference - Trivial |
| Bouguezzi et al. (2018) B (3) | M, n=15, Elite Soccer Players; Age 12.27±0.33 years | Plyometrics training 2d/wk, 8wks, 16 sessions, In-season | Moderate volume (6-12 sets of 8-12 reps/set) moderate intensity plyometric training of unilateral and bilateral acyclical exercises (CMJ's, ankle Hops, zig-zags) | See Bouguezzi et al. (2018) A | 0-30m performance ↓ MD (s): -0.05; 95% CI [-0.09, -0.01]; % Change -0.93%;  SMD: -0.23; 95% CI [-0.44, -0.03] Weight 2.28%; Inference - Small |
| Chelly et al. (2010) A (4) | M, n=12, Elite Soccer Players; Age 19.1±0.7 years | Strength and plyometrics training 2d/wk, 8wks, 16 sessions, In-season | Moderate-high volume (10 sets of 4-10 reps/set) high intensity plyometric training (wk1-4 40-60cm hurdle jumps and wk 5-8 40cm bilateral drop jumps) on a grass track and a light UB and LB resistance training programme (not identified) | Weekly school physical education sessions; these lasted for 40 minutes and consisted mainly of ball games.  Video cameras (Sony Handycam, DCRPC105E, 2003 Sony corporation, Tokyo, Japan) | Vmax ↑ MD (m·s^-1^): 0.8; 95% CI [0.76, 0.84]; % Change 9.76%;  SMD: 4; 95% CI [3.46, 4.54] Weight 7.92%; Inference - Large |
| Christou et al. (2006) A (5) | M, n=9, Elite Soccer Players; Age 13.8±0.4 years | Strength training 2d/wk, 16wks, 32 sessions, In-season | "Moderate-high load (55-80% 1RM) FB strength training 2-3 sets of 8-15 reps/set of leg press, bench press, leg extension, | Strength training 2d/wk, 16wks, 32 sessions, In-season | 0-30m performance ↑ MD (s): 0.13; 95% CI [0.09, 0.17]; % Change 2.63%;  SMD: 0.76; 95% CI [0.46, 1.06] Weight 2.18%; Inference - Moderate |
| Coratella et al. (2019) A (6) | M, n=16, Sub-elite Soccer Players; Age 21±3 years | Power training 2d/wk, 8wks, 16 sessions, Off-season | 2-5 sets of 10 reps/set of BW jump squat power training | NA  Infrared device (Polifemo, Microgate, Bolzano, Italy) | 0-30m performance = MD (s): 0; 95% CI [-0.04, 0.04]; % Change 0%;  SMD: 0; 95% CI [-0.2, 0.2] Weight 2.29%; Inference - Trivial |
| Coratella et al. (2019) B (6) | M, n=16, Sub-elite Soccer Players; Age 21±3 years | Power training 2d/wk, 8wks, 16 sessions, Off-season | 2-4 sets of 10-11 reps/set of +20-25%BW jump squat power training | See Coratella et al. (2019) A | 0-30m performance ↑ MD (s): 0.2; 95% CI [0.16, 0.24]; % Change 4.55%;  SMD: 1; 95% CI [0.76, 1.24] Weight 2.25%; Inference - Large |
| De Hoyo et al. (2016) A (7) | M, n=11, Elite Soccer Players; Age 18±1 years | Power training 2d/wk, 8wks, 16 sessions, In-season | Light-moderate loads squat (40-60%1RM) moderate volume (2-3 sets of 4-8 reps/set) using a full range back squat | All players participated on an average of ;10 hours of combined soccer (4– 5 sessions) and conditioning (1 session) training, and 1 competitive match per week.  Dual-beam electronic timing gate OptoJump System (Polifemo Radio Light, Microgate, Bolzano, Italy) | 0-30m performance ↑ MD (s): 0.04; 95% CI [0.01, 0.07]; % Change 0.98%;  SMD: 0.34; 95% CI [0.1, 0.58] Weight 2.25%; Inference – Small  >30m performance ↑ MD (s): 0.13; 95% CI [0.1, 0.16]; % Change 1.88%;  SMD: 0.61; 95% CI [0.35, 0.87] Weight 8.39%; Inference - Moderate |
| Douglas et al. (2018) A (8) | M, n=7, Elite Rugby Players; Age 19.4±0.8 years | Strength and power training 2d/wk, 4wks, 8 sessions, In-season | 2 strength sessions/wk + 1 power session. Strength sessions: AEL smith machine back squat, assistance LB and UB assistance exercise for 3–4 sets of 6–8 reps/set. Power training session: AEL broad jump (30% BM), partner AEL KB swing and an assistance FB power for 3–4 sets of 4–6 reps/set. | A combination of conditioning- and skill-based field sessions, and a club rugby game each week  Radar gun device (Stalker ATS II; Applied Concepts, Dallas, TX, USA) | 0-30m performance ↑ MD (s): 0.05; 95% CI [0.01, 0.09]; % Change 0.93%;  SMD: 0.36; 95% CI [0.06, 0.67] Weight 2.17%; Inference – Small  Vmax ↑ MD (m·s^-1^): 0.15; 95% CI [0.08, 0.22]; % Change 1.72%;  SMD: 0.52; 95% CI [0.27, 0.77] Weight 9.15%; Inference - Moderate |
| Douglas et al. (2018) B (8) | M, n=7, Elite Rugby Players; Age 19.4±0.8 years | Strength and power training 2d/wk, 10wks, 18 sessions, In-season | 4 wks slow + 4 weeks fast accentuated eccentric training consisting of 2 strength sessions/wk + 1 power session. Slow Strength sessions: AEL smith machine back squat, assistance LB and UB assistance exercise for 2–4 sets of 6–8 reps/set. Fast strength sessions: AEL smith machine back squat, assistance LB and UB assistance exercise for 2–4 sets of 4–5 reps/set. Slow Power training session: AEL broad jump (30% BM), partner AEL KB swing and an assistance FB power for 3–4 sets of 4–6 reps/set. Fast power training session: 50cm AEL drop Jump (20% BM), Partner AEL Banded KB Swing, assistance FB power exercise. | See Douglas et al. (2018) A | 0-30m performance = MD (s): -0.03; 95% CI [-0.07, 0.01]; % Change -0.55%;  SMD: -0.22; 95% CI [-0.52, 0.08] Weight 2.18%; Inference – Small  Vmax = MD (m·s^-1^): 0; 95% CI [-0.06, 0.06]; % Change 0%;  SMD: 0; 95% CI [-0.23, 0.23] Weight 9.2%; Inference - Trivial |
| Douglas et al. (2018) C (8) | M, n=7, Elite Rugby Players; Age 19.4±0.8 years | Strength and power training 2d/wk, 4wks, 8 sessions, In-season | Slow strength training consisting of 2 strength sessions/wk + 1 power session. Strength sessions: back squat, assistance LB and UB assistance exercise for 3–4 sets of 6–8 reps/set. Power training session: broad jump (30% BM), KB swing and an assistance FB power exercise for 3–4 sets of 4–6 reps/set. | See Douglas et al. (2018) A | 0-30m performance = MD (s): -0.01; 95% CI [-0.09, 0.07]; % Change -0.18%;  SMD: -0.04; 95% CI [-0.33, 0.26] Weight 2.18%; Inference – Trivial  Vmax = MD (m·s^-1^): -0.05; 95% CI [-0.14, 0.04]; % Change -0.6%;  SMD: -0.13; 95% CI [-0.36, 0.11] Weight 9.2%; Inference - Trivial |
| Douglas et al. (2018) D (8) | M, n=7, Elite Rugby Players; Age 19.4±0.8 years | Strength and power training 2d/wk, 10wks, 18 sessions, In-season | 4 wks slow + 4 weeks fast strength training consisting of 2 strength/wk + 1 power session. Slow strength sessions: back squat, assistance LB and UB assistance exercise for 2–4 sets of 6–8 reps/set. Fast strength sessions: back squat, assistance LB and UB assistance exercise for 2–4 sets of 4–5 reps/set. Slow power training session: broad jump, KB swing and an assistance FB power exercise for 3–4 sets of 4–6 reps/set. Fast power training session: 50cm drop Jump, banded KB, assistance FB power exercise. | See Douglas et al. (2018) A | 0-30m performance = MD (s): -0.05; 95% CI [-0.13, 0.03]; % Change -0.87%;  SMD: -0.19; 95% CI [-0.49, 0.11] Weight 2.18%; Inference – Trivial  Vmax ↓ MD (m·s^-1^): -0.16; 95% CI [-0.25, -0.07]; % Change -1.93%;  SMD: -0.42; 95% CI [-0.66, -0.17] Weight 9.17%; Inference - Moderate |
| Escobar-Álvarez et al. (2019) A (9) | Elite Rugby Sevens Players; Age 26.2±5.4 years | Strength training 2d/wk, 6wks, 12 sessions, Phase not reported | Velocity based high load (70-85% 1RM) LB strength training 3 sets of 8 reps/set of half squat and deadlift. | NA  Timing gates (Smart Speed, Fusion Sport, Queensland, Australia) | 0-30m performance ↑ MD (s): 0.03; 95% CI [0.01, 0.05]; % Change 0.72%;  SMD: 0.43; 95% CI [0.17, 0.69] Weight 2.23%; Inference - Moderate |
| Gabbett et al. (2008) A (10) | M, n=14, Elite Rugby league Players; Age 14.1±0.2 years | Strength training 3d/wk, 10wks, 30 sessions, Pre-season | Moderate load strength training (2-3 sets of 12-15 reps/set) for 6wks followed by (3-4 sets of 8 to 12 reps/set) for 4wks of LB (leg press) and UB (bench press, shoulder press, wide grip pull-down, and chin-ups) using machine weights | Repeated sprint training as well as pre-season technical tactical training  Dual-beam electronic timing gates (Swift Performance Equipment). | 0->30m performance ↑ MD (s): 0.1; 95% CI [0.08, 0.12]; % Change 1.76%;  SMD: 1.17; 95% CI [0.9, 1.44] Weight 7.72%; Inference - Large |
| Gabbett et al. (2008) B (10) | M, n=21, Elite Rugby league Players; Age 16.9±0.3 years | Strength training 3d/wk, 10wks, 30 sessions, Pre-season | See Gabbett et al. (2008) A | See Gabbett et al. (2008) A | >30m performance ↓ MD (s): 0.07; 95% CI [0.05, 0.09]; % Change -3.26%;  SMD: -2.22; 95% CI [-2.54, -1.9] Weight 8.28%; Inference - Large |
| Garci´a-Pinillos et al. (2014) A (11) | M, n=17, Elite Soccer Players; Age 15.47±1.28 years | Strength and plyometrics training 2d/wk, 12wks, 24 sessions, In-season | Moderate volume 4-6 sets of isometric wall squat (half squat 40–80 seconds) and low-moderate intensity plyometric training using jumping from a seated position and single-leg jumping using arm swing (6 reps/set) | Soccer training 3 times/wk and played competitive matches at least once/wk  Casio Exilim EXZR-10 high speed camera (Dover, NJ, USA) | 0-30m performance ↑ MD (s): 0.32; 95% CI [0.25, 0.39]; % Change 6.68%;  SMD: 0.92; 95% CI [0.69, 1.15] Weight 2.26%; Inference - Large |
| Hammami et al. (2016) 1A (12) | M, n=15, Soccer Players; Age 15.7±0.2 years | Plyometrics training 2d/wk, 8wks, 16 sessions, In-season | High intensity moderate volume (4-10 sets of 7-10 foot contacts/set) plyometric training (hurdle jumps 50-60cm and drop jumps 60-70cm) | Soccer training 4-5 times/wk, and one official soccer match every Sunday  Photocells system (Microgate, Bolzano, Italy) | 0-30m performance ↑ MD (s): 0.16; 95% CI [0.12, 0.2]; % Change 3.68%;  SMD: 0.75; 95% CI [0.52, 0.98] Weight 2.26%; Inference – Moderate  >30m performance ↑ MD (s): -0.2; 95% CI [-0.32, -0.08]; % Change 3.96%;  SMD: 0.83; 95% CI [0.6, 1.07] Weight 8.42%; Inference - Large |
| Hammami et al. (2018) B (13) | M, n=14, Elite Soccer Players; Age 15.7±0.2 years | Plyometrics training 2d/wk, 8wks, 16 sessions, In-season | High intensity moderate volume (4-10 sets of 7-10 foot contacts/set) plyometric training (hurdle jumps 50-60cm and drop jumps 60-70cm) on a tartan track | Soccer training 4–5 times/wk and 1 official game/wk Paired photocell timers (Microgate, Bolzano, Italy) | >30m performance ↑ MD (s): 0.5; 95% CI [0.36, 0.64]; % Change 3.6%;  SMD: 0.77; 95% CI [0.53, 1.01] Weight 8.42%; Inference - Moderate |
| Karsten et al. (2016) A (14) | M, n=13, Non-elite Soccer Players; Age 18±1 years | Strength training 2d/wk, 6wks, 12 sessions, Pre-season | FB strength training 2 wks of 3 sets of 12 reps/set at 60-65%1RM and 4 wks of 3 sets of 4 reps at 70-75%1RM of parallel squat, lunges, RDL and dumbbell upright row and bench press. | Soccer training 3 d/wk  Timing gates | 0-30m performance ↑ MD (s): 0.12; 95% CI [0.07, 0.17]; % Change 2.63%;  SMD: 0.5; 95% CI [0.27, 0.73] Weight 2.26%; Inference - Moderate |
| Krommes et al. (2017) A (15) | M, n=9, Elite Soccer Players; Age 23±3.9 years | Strength training 1-3d/wk, 10wks, 27 sessions, Pre-season | Nordic curls 2-3 sets of 5-12 reps/set | N/A Electronic timing gates (Newtest, Oulu, Finland) | 0-30m performance ↓ MD (s): -0.1; 95% CI [-0.14, -0.06]; % Change -2.38%;  SMD: -0.68; 95% CI [-0.97, -0.39] Weight 2.19%; Inference - Moderate |
| Loturco et al. (2013) A (16) | M, n=16, Elite Soccer Players; Age 19.18±0.72 years | Strength and power training 2d/wk, 6wks, 12 sessions, Pre-season | 3wk LB strength mesocycle followed by a 3 wk LB power mesocycle with a reducing jump squat load. wk1-3, 4 sets of 6-8 reps/set @50-80% 1RM of back squat. Wk4-6, 4 sets of 4-6 reps/set @ 30-60% 1RM of jump squat. The load reduced from 60% 1RM to 30% 1RM across the 3 wks. | Training for general development of soccer-specific technical-tactical skills (4 times/wk).  Photocells (Smart Speed; Fusion Equipment) | 0-30m performance ↑ MD (s): 0.06; 95% CI [0.03, 0.09]; % Change 1.37%;  SMD: 0.43; 95% CI [0.22, 0.63] Weight 2.28%; Inference - Moderate |
| Loturco et al. (2013) B (16) | M, n=16, Elite Soccer Players; Age 19.11±0.7 years | Strength and power training 2d/wk, 6wks, 12 sessions, Pre-season | See Loturco et al. (2013) A, instead the load increases from 30% 1RM to 60% 1RM across the 3 wks | See Loturco et al. (2013) A | 0-30m performance = MD (s): -0.02; 95% CI [-0.05, 0.01]; % Change -0.34%;  SMD: -0.13; 95% CI [-0.33, 0.06] Weight 2.29%; Inference - Trivial |
| Loturco et al. (2016) A (17) | M, n=9, Elite Soccer Players; Age 18.4±1.2 years | Power training 2d/wk, 6wks, 12 sessions, Pre-season | Velocity based training performing 6 sets of 4-8 reps/set of jump squats performed @ a load corresponding to the mass at which optimal power is produced (1-1.1* optimal power load) | Small sided game training 6/wk with a friendly on the Saturday.  Photocells (Smart Speed; Fusion Equipment) | 0-30m performance ↑ MD (s): 0.22; 95% CI [0.16, 0.28]; % Change 3%;  SMD: 1.02; 95% CI [0.69, 1.34] Weight 2.15%; Inference - Large |
| Loturco et al. (2016) B (17) | M, n=8, Elite Soccer Players; Age 18.4±1.2 years | Power training 2d/wk, 6wks, 12 sessions, Pre-season | Velocity based training performing 6 sets of 4-8 reps/set of overhead push press performed @ a load corresponding to the mass at which optimal power is produced (1-1.1* optimal power load) | See Loturco et al. (2016) B | 0-30m performance = MD (s): -0.04; 95% CI [-0.15, 0.07]; % Change -0.54%;  SMD: -0.1; 95% CI [-0.38, 0.17] Weight 2.21%; Inference - Trivial |
| Loturco et al. (2017) B (18) | M, n=11, Elite Soccer Players; Age 22.2±2.4 years | Power and plyometrics training 2-3d/wk, 5wks, 12 sessions, Pre-season | Velocity based training performing 6 sets of 4-8 reps/set of jump squats performed @ a load corresponding to the mass at which optimal power is produced + 6-8 sets of 6 reps/set of moderate intensity plyometrics (wk 1-2 horizontal, wk 3-4 mixed, wk 5-6 vertical CMJ's) | Pre-season soccer training 6/week + 2 pre-season friendly games  Photocells (Smart Speed, Fusion Equipment, AUS) | 0-30m performance ↑ MD (s): 0.06; 95% CI [0.03, 0.09]; % Change 1.49%;  SMD: 0.54; 95% CI [0.29, 0.79] Weight 2.23%; Inference - Moderate |
| Manouras et al. (2016) A (19) | M, n=10, Sub-elite Soccer Players; Age 19.1±5.75 years | Plyometrics training 1d/wk, 8wks, 8 sessions, In-season | Moderate-high intensity horizontal plyometric training (ankle jumps, long jumps, obstacle jumps and drop jumps) for 3-5 sets of 4-10 foot contacts/set. | Soccer training 3 times/wk and one official game/wk  Electronic timing lights (Newtest PowerTimer, Newtest Oy, Oulu, Finland) | 0-30m performance ↑ MD (s): 0.1; 95% CI [0.05, 0.15]; % Change 2.78%;  SMD: 0.53; 95% CI [0.26, 0.79] Weight 2.22%; Inference - Moderate |
| Manouras et al. (2016) B (19) | M, n=10, Sub-elite Soccer Players; Age 20.75±6.14 years | Plyometrics training 1d/wk, 8wks, 8 sessions, In-season | Moderate-high intensity horizontal plyometric training (ankle jumps, CMJs, obstacle jumps and drop jumps) for 3-5 sets of 4-10 foot contacts/set. | See Manouras et al. (2016) A | 0-30m performance ↑ MD (s): 0.11; 95% CI [0.06, 0.16]; % Change 3.11%;  SMD: 0.51; 95% CI [0.25, 0.78] Weight 2.22%; Inference - Moderate |
| McMaster et al. (2014) A (20) | M, n=6, Elite Rugby union Players; Age 20.9±1.6 years | Strength and power training 4d/wk, 5wks, 20 sessions, Pre-season | 4d complex training wk Monday: CMJs, countermovement bench throws, power cleans, 1 arm jammer press, 1 arm DB snatch. Tuesday: bench press, concentric-only bench throws, weighted chin ups, high pulls, 1 arm DB floor press, 1 arm DB rows. Thursday: back squat, ¼ squat jumps, Bulgarian split squats and (off-squat rack) and speed lunge (elevated landing) Friday: incline DB press, alternate arm DB bench press, box squat, hip thrusts and calf raises). Strength exercises (4-8 sets of 2-6 reps/set @ 85-99% 1RM) and Heavy ballistic exercises (4-5 sets of 2-4 reps @ 60-75% 1RM) | N/A  Dual beam infrared timing lights (Swift Performance, Lismore, Australia) | 0-30m performance = MD (s): 0.03; 95% CI [0, 0.06]; % Change 0.72%;  SMD: 0.3; 95% CI [-0.03, 0.63] Weight 2.15%; Inference - Small |
| McMaster et al. (2014) B (20) | M, n=8, Elite Rugby union Players; Age 20.9±1.6 years | Strength and power training 4d/wk, 5wks, 20 sessions, Pre-season | Four-day complex training week Monday: CMJs, countermovement bench throws, power cleans, 1 arm jammer press, 1 arm DB snatch. Tuesday: bench press, concentric-only bench throws, weighted chin ups, high pulls, 1 arm DB floor press, 1 arm DB rows. Thursday: back squat, ¼ squat jumps, Bulgarian split squats and (off-squat rack) and speed lunge (elevated landing) Friday: incline DB press, Alternate arm DB bench press, box squat, hip thrusts and calf raises). Strength exercises (4-8 sets of 2-6 reps/set @ 85-99% 1RM) and light ballistic exercises (6-8 sets of 5-8 reps/set @ 15-30%1RM) | See McMaster et al. (2014) A | 0-30m performance ↑ MD (s): 0.03; 95% CI [0, 0.06]; % Change 0.72%;  SMD: 0.28; 95% CI [0, 0.56] Weight 2.2%; Inference - Small |
| Negra et al. (2016) A (21) | M, n=13, Elite Soccer Players; Age 12.8±0.25 years | Power training 2d/wk, 12wks, 24 sessions, In-season | Low-moderate load (40-60%) high volume high-velocity power training 4 sets of 8–12 rep/set of half squats + abdominal curl and back extension exercises 6 sets of 15 reps/set. | Regular soccer training with 3 sessions/wk and 1 game/wk.  Single-beam photoelectric gates | 0-30m performance ↑ MD (s): 0.21; 95% CI [0.13, 0.29]; % Change 4.32%;  SMD: 0.6; 95% CI [0.36, 0.84] Weight 2.25%; Inference - Moderate |
| Orange et al. (2019) A (22) | M, n=15, Elite Rugby league Players; Age 17±1 years | Strength training 2d/wk, 7wks, 14 sessions, In-season | 2-4 sets of FB strength training (60-80%1RM or 1-2 reps in reserve), 2-4 sets of 5-10 reps/set. Session 1, back squat, nordic lower, incline DB bench, BB bent over row, front plank. Session 2, back squat, SL RDL, DB push press, Pull Ups, Barbell Rollout. | Rugby league skills and conditioning 2d/wk, low-intensity team run 24hr pre games and recovery session 48 hrs post-match  Photocell timing system (Witty Timing System, Microgate, Balzano, Italy) | 0-30m performance ↓ MD (s): -0.1; 95% CI [-0.13, -0.07]; % Change -2.33%;  SMD: -0.65; 95% CI [-0.87, -0.43] Weight 2.27%; Inference - Moderate |
| Orange et al. (2019) B (22) | M, n=12, Elite Rugby league Players; Age 17±1 years | Strength training 2d/wk, 7wks, 14 sessions, In-season | 2-4 sets of FB velocity based strength training (60-80%1RM or 1-2 reps in reserve), 2-4 sets of 5-10 reps/set. Session 1, back squat, nordic lower, incline DB bench, BB bent over row, front plank. Session 2, back squat, SL RDL, DB push press, Pull Ups, Barbell Rollout. | See Orange et al. (2019) A | 0-30m performance ↓ MD (s): -0.09; 95% CI [-0.14, -0.04]; % Change -2.06%;  SMD: -0.39; 95% CI [-0.62, -0.15] Weight 2.25%; Inference - Small |
| Ramirez-Campillo et al. (2015) 1A (23) | M, n=10, Soccer Players; Age 11.6±1.4 years | Plyometrics training 2d/wk, 6wks, 12 sessions, In-season | Mod-high intensity low volume (3 sets of 5-10 reps/set) vertical plyometrics (bilateral and unilateral cyclic and acyclic jumps) on a grass pitch surface | Soccer training 3x/wk  Single beam infrared photoelectric cells (Globus Italy) | 0-30m performance = MD (s): 0.15; 95% CI [-0.04, 0.34]; % Change 2.55%;  SMD: 0.2; 95% CI [-0.05, 0.45] Weight 2.24%; Inference - Small |
| Ramirez-Campillo et al. (2015) 1B (23) | M, n=10, Soccer Players; Age 11.4±1.9 years | Plyometrics training 2d/wk, 6wks, 12 sessions, In-season | Mod-high intensity low volume (3 sets of 5-10 reps/set) horizontal plyometrics (bilateral and unilateral cyclic and acyclic jumps) on a grass pitch surface | See Ramirez-Campillo et al. (2015) 1A | 0-30m performance ↑ MD (s): 0.24; 95% CI [0.09, 0.39]; % Change 4.18%;  SMD: 0.39; 95% CI [0.13, 0.65] Weight 2.23%; Inference - Small |
| Ramirez-Campillo et al. (2015) 1C (23) | M, n=10, Soccer Players; Age 11.2±2.3 years | Plyometrics training 2d/wk, 6wks, 12 sessions, In-season | Mod-high intensity low volume (2 sets of 5-10 reps/set) vertical + horizontal plyometrics (bilateral and unilateral cyclic and acyclic jumps) | See Ramirez-Campillo et al. (2015) 1A | 0-30m performance ↑ MD (s): 0.36; 95% CI [0.23, 0.49]; % Change 6.24%;  SMD: 0.7; 95% CI [0.43, 0.98] Weight 2.21%; Inference - Moderate |
| Ramirez-Campillo et al. (2015) 2A (24) | M, n=12, Sub-elite Soccer Players; Age 11±2 years | Plyometrics training 2d/wk, 6wks, 12 sessions, Phase not reported | Mod-high intensity low volume (3 sets of 5-10 reps/set) horizontal plyometrics (bilateral and unilateral cyclic and acyclic jumps) on a grass pitch surface | Soccer training 3x/wk and 1 game/wk Single beam infrared photoelectric cells (Globus Italy) | 0-30m performance ↑ MD (s): 0.16; 95% CI [0.05, 0.27]; % Change 2.89%;  SMD: 0.33; 95% CI [0.09, 0.56] Weight 2.26%; Inference - Small |
| Ramirez-Campillo et al. (2015) 2B (24) | M, n=16, Sub-elite Soccer Players; Age 11.6±1.7 years | Plyometrics training 2d/wk, 6wks, 12 sessions, Phase not reported | Mod-high intensity low volume (3 sets of 5-10 reps/set) horizontal plyometrics (bilateral and unilateral cyclic and acyclic jumps) on a grass pitch surface | See Ramirez-Campillo et al. (2015) 2A | 0-30m performance ↑ MD (s): 0.37; 95% CI [0.21, 0.53]; % Change 6.46%;  SMD: 0.46; 95% CI [0.25, 0.66] Weight 2.28%; Inference - Moderate |
| Ramirez-Campillo et al. (2015) 2C (24) | M, n=12, Sub-elite Soccer Players; Age 11.6±2.7 years | Plyometrics training 2d/wk, 6wks, 12 sessions, Phase not reported | Mod-high intensity low volume (3 sets of 5-10 reps/set) horizontal plyometrics (bilateral and unilateral cyclic and acyclic jumps) on a grass pitch surface | See Ramirez-Campillo et al. (2015) 2A | 0-30m performance ↑ MD (s): 0.24; 95% CI [0.11, 0.37]; % Change 4.17%;  SMD: 0.41; 95% CI [0.17, 0.64] Weight 2.25%; Inference - Moderate |
| Ramirez-Campillo et al. (2015) 3A (25) | F, n=19 Non-elite Soccer Players; Age 22.4±2.4 years | Plyometrics training 2d/wk, 6wks, 12 sessions, In-season | Mod-high intensity low volume (2 sets of 5-10 reps/set) vertical plyometrics (Bi-lateral and unilateral cyclic and acyclic jumps) | N/A  Single-beam infrared photoelectric cells (Globus Italia, Codogne, Italy) | 0-30m performance ↑ MD (s): 0.29; 95% CI [0.23, 0.35]; % Change 5.37%;  SMD: 0.92; 95% CI [0.7, 1.13] Weight 2.27%; Inference - Large |
| Ramirez-Campillo et al. (2015) 3B (25) | M, n=21, Non-elite Soccer Players; Age 20.4±2.8 years | Plyometrics training 2d/wk, 6wks, 12 sessions, In-season | See Ramirez-Campillo et al. (2015) 3A | See Ramirez-Campillo et al. (2015) 3A | 0-30m performance ↑ MD (s): 0.26; 95% CI [0.23, 0.29]; % Change 5.43%;  SMD: 1.47; 95% CI [1.22, 1.72] Weight 2.24%; Inference - Large |
| Ramirez-Campillo et al. (2019) A (26) | M, n=8, Elite Soccer Players; Age 13.9±1.9 years | Plyometrics training 2d/wk, 8wks, 16 sessions, In-season | Mod-high intensity (1 set of 7-14 reps/set) vertical + horizontal plyometrics (drop jumps, standing long jumps, unilateral countermovement jumps, 180 degrees jumps and repeated countermovement jumps on a grass pitch surface | N/A  Single-beam timing gates (Brower® Timing System, Salt Lake City, Utha, USA). | 0-30m performance ↑ MD (s): 0.31; 95% CI [0.12, 0.5]; % Change 5.76%;  SMD: 0.45; 95% CI [0.16, 0.74] Weight 2.19%; Inference - Moderate |
| Ramirez-Campillo et al. (2019) B (26) | M, n=8, Elite Soccer Players; Age 13.1±1.7 years | Plyometrics training 2d/wk, 8wks, 16 sessions, In-season | Mod-high intensity (1 set of 7-14 reps/set) vertical + horizontal plyometrics (individualised box heights drop jumps, standing long jumps, unilateral countermovement jumps, 180 degrees jumps and repeated countermovement jumps) 2d/wk on grass, land-dirt, sand, wood, gym mat, and tartan track surfaces. The surface and order of surface were randomised each week. | See Ramirez-Campillo et al. (2019) A | 0-30m performance ↑ MD (s): 0.57; 95% CI [0.35, 0.79]; % Change 10.18%;  SMD: 0.73; 95% CI [0.41, 1.04] Weight 2.17%; Inference - Moderate |
| Randell et al. (2011) A (27) | M, n=7, Elite Rugby Players; Age 25.7±3.6 years | Strength and power training 3d/wk, 6wks, 18 sessions, Pre-season | Low-high load FB strength/ strength-speed exercises for 3-5 sets of 6-10 reps/set @RM load. Squat jumps where performed 1st on Wednesdays and Friday @40kg load (3 sets of 3 reps/set) with instantaneous performance feedback (peak velocity) provided after each repetition of squat jump. Strength exercises consisted of horizontal and vertical pushing exercises, squatting and deadlift patterns as well as injury prevention exercises. | Other conditioning sessions involved a energetic and skills focus  Wireless timing lights | 0-30m performance ↑ MD (s): 0.06; 95% CI [0.03, 0.09]; % Change 1.45%;  SMD: 0.55; 95% CI [0.23, 0.86] Weight 2.16%; Inference - Moderate |
| Randell et al. (2011) B (27) | M, n=6, Elite Rugby Players; Age 24.2±2.5 years | Strength and power training 3d/wk, 6wks, 18 sessions, Pre-season | Low-high load FB strength/ strength-speed exercises for 3-5 sets of 6-10 reps/set @RM load. Squat jumps where performed 1st on Wednesdays and Friday @40kg load (3 sets of 3 reps/set) with no instantaneous performance feedback provided. Strength exercises consisted of horizontal and vertical pushing exercises, squatting and deadlift patterns as well as injury prevention exercises. | See Randell et al. (2011) A | 0-30m performance = MD (s): -0.01; 95% CI [-0.08, 0.06]; % Change -0.23%;  SMD: -0.05; 95% CI [-0.37, 0.27] Weight 2.15%; Inference - Trivial |
| Rimmer and Sleivert (2000) A (28) | M, n=10, Sub-elite Rugby + Touch Rugby Players; Age 24.4±4 years | Plyometrics training 1-2d/wk, 8wks, 15 sessions, Phase not reported | High-intensity plyometric training high volume (2-5 sets of 5-12 foot contacts) of bilateral and unilateral tuck jumps, speed jumps, hops, bounds and stair bounds on a grass surface. | N/A  Digital timer (University of Otago, Dunedin, New Zealand) | Vmax ↑ MD (s): 0.02; 95% CI [0.01, 0.03]; % Change 1.69%;  SMD: 0.5; 95% CI [0.29, 0.71] Weight 9.28%; Inference - Moderate |
| Ronnestad et al. (2008) A (29) | M, n=6, Elite Soccer Players; Age 22±2.5 years | Strength training 2d/wk, 7wks, 14 sessions, Phase not reported | High loads (85-90% 1RM) and low volumes (3-5 sets and 4-6 reps/set) of half squats and hip flexions. | 6-8 soccer training sessions/wk  Photocells (JBL Systems, Oslo, Norway) | >30m performance ↑ MD (s): 0.26; 95% CI [0.23, 0.29]; % Change 1.3%;  SMD: 0.82; 95% CI [0.45, 1.19] Weight 8.18%; Inference – Large  Vmax ↑ MD (s): 0.01; 95% CI [0, 0.02]; % Change 0.85%;  SMD: 0.5; 95% CI [0.23, 0.77] Weight 9.09%; Inference - Moderate |
| Ronnestad et al. (2008) B (29) | M, n=8, Elite Soccer Players; Age 23±2 years | Strength and plyometrics training 2d/wk, 7wks, 14 sessions, Phase not reported | High loads (85-90% 1RM) and low volumes (3-5 sets and 4-6 repetitions/set) of half squats and hip flexions + high-intensity low volume (2-4 sets and 5-10 reps /set) of Plyometrics (bounding hurdle jumps and single leg hops) | See Ronnenstad et al. (2008) A | >30m performance ↑ MD (s): 0.22; 95% CI [0.15, 0.29]; % Change 1.12%;  SMD: 0.86; 95% CI [0.54, 1.19] Weight 8.27%; Inference – Large  Vmax ↑ MD (s): 0.01; 95% CI [0, 0.02]; % Change 0.85%;  SMD: 0.29; 95% CI [0.07, 0.51] Weight 9.23%; Inference - Small |
| Ronnenstad et al. (2011) A (30) | M, n=7, Elite Soccer Players; Age 22±2 years | Strength training 2d/wk in pre-season + 1d/wk In-season, 22wks, 32 sessions, Pre-season and in-season | Moderate-high loads (70-90%1RM) and low volumes (3 sets and 4-10 repes/set) of half squat. | Pre-season 1 game/wk, in-season 1-2 games/wk + 5-7 training sessions/wk  Photo cells (Speed trap 2, Brower Timing Systems, Utah, USA) | >30m performance = MD (s): -0.3; 95% CI [-0.38, -0.22]; % Change -0.19%;  SMD: -0.06; 95% CI [-0.36, 0.24] Weight 8.32%; Inference - Trivial |
| Ronnenstad et al. (2011) B (30) | M, n=7, Elite Soccer Players; Age 26±2 years | Strength training 2 per week in pre-season + 0.5 times per week In-season d/wk, 22wks, 26 sessions, Pre-season and in-season | Moderate-high loads (70-90%1RM) and low volumes (3 sets and 4-10 reps/set) of half squat. | Pre-season 1 game/wk, in-season 1-2 games/wk + 5-7 training sessions/wk Photo cells (Speed trap 2, Brower Timing Systems, Utah, USA) | >30m performance ↓ MD (s): 0.11; 95% CI [0.04, 0.18]; % Change -1.13%;  SMD: -0.35; 95% CI [-0.66, -0.05] Weight 8.31%; Inference - Small |
| Scott et al. (2017) A (31) | M, n=10, Elite Ausi Football Players; Age 19.8±1.5 years | Strength training 2-3d/wk, 5wks, 14 sessions, Pre-season | Moderate-heavy loads (75-85% 1RM) and high volumes (3-4 sets of 6-10 repetitions/set) of UB and LB strength training + low volume (3-4 sets and 6 reps /set) of power training (Jump shrug, broad jumps) + supplementary very low loads (20-30% 1RM) high volume (4 sets of 15-30 reps/set) blood flow restriction training (squats) | Normal resistance training programs, as prescribed by the coaching staff. Football training was typified by tactical, technical, and match simulation drills involving running, sprinting, change of direction, jumping, collisions, and kicking.  Wireless electronic timing gate system (SpeedLight; Swift Performance, Brisbane, Australia) | >30m performance ↓ MD (s): 0.04; 95% CI [-0.01, 0.09]; % Change -0.74%;  SMD: -0.26; 95% CI [-0.51, -0.01] Weight 8.4%; Inference – Small  Vmax = MD (s): 0.02; 95% CI [0, 0.04]; % Change 0.85%;  SMD: 0.16; 95% CI [-0.04, 0.36] Weight 9.3%; Inference - Trivial |
| Scott et al. (2017) B (31) | M, n=8, Elite Ausi Football Players; Age 19.8±1.5 years | Strength training 2-3d/wk, 5wks, 14 sessions, Pre-season | Moderate-heavy loads (75-85% 1RM) and moderate volumes (3-4 sets of 6-10 repetitions/set) of UB and LB strength training + low volume (3-4 sets of 6 reps /set) of power training (Jump shrug, broad jumps) + supplementary very light loads (20-30% 1RM) high volume (4 sets of 15-30 reps/set) non blood flow restriction training (squats) | See Scott et al. (2017) A | >30m performance = MD (s): 0.03; 95% CI [-0.02, 0.08]; % Change 0%;  SMD: 0; 95% CI [-0.28, 0.28] Weight 8.36%; Inference – Trivial  Vmax = MD (s): 0; 95% CI [-0.02, 0.02]; % Change 0%;  SMD: 0; 95% CI [-0.22, 0.22] Weight 9.25%; Inference - Trivial |
| Shalfawi et al. (2012) B (32) | M, n=7, Elite Soccer Players; Age 16.3±0.5 years | Strength training 2d/wk, 8wks, 16 sessions, Phase not reported | Resistance training nordic curl, balance training (ankle strength on balance board), sit-ups, the plank, push-ups and the alternating back and arm raise 2 times/wk during soccer training (volume/ intensity not provided). | Soccer training 4 sessions/wk  Newtest Powertimer 300s infrared photocells | >30m performance ↑ MD (s): 0.07; 95% CI [0.03, 0.11]; % Change 1.97%;  SMD: 0.45; 95% CI [0.14, 0.76] Weight 8.3%; Inference – Moderate  Vmax = MD (s): 0; 95% CI [-0.03, 0.03]; % Change 0%;  SMD: 0; 95% CI [-0.23, 0.23] Weight 9.2%; Inference - Trivial |
| So¨hnlein et al. (2014) A (33) | M, n=12, Elite Soccer Players; Age 13±0.9 years | Plyometrics training 2d/wk, 16wks, 32 sessions, In-season | Low-high intensity and moderate-high volume (2-5 sets of 6-16 reps/set) plyometrics training. Day 1 vertical horizontal focused exercises (2-footed ankle hop forward, hurdle jumps, single leg hop forward, and squat jump). Day 2 Lateral focused exercises (lateral bound stabilisation, lateral hurdle jumps, double leg zigzag, and single leg hop lateral). 2 (of 8) slow SSC-type, 6 (of 8) fast SSC-type plyometric exercises | 4-5 soccer training session/wk + 1 game/wk  Infrared photoelectric gate (Brower Timing Systems, Draper, UT, USA) | 0-30m performance ↑ MD (s): 0.11; 95% CI [0.07, 0.15]; % Change 2.58%;  SMD: 0.56; 95% CI [0.31, 0.8] Weight 2.25%; Inference - Moderate |
| Tous-Fajardo et al. (2016) A (34) | M, n=12, Soccer Players; Age 17±0.5 years | Strength training 1d/wk, 11wks, 11 sessions, In-season | Exercises performed in a circuit fashion, of moderate volume (2 sets of 6-8 reps/sets) resistance training exercises consisted of reverse wood chops, backward lunges, unilateral hamstrings “kicks” using an isoinertial portable conical pulley, lateral squats employing the Yo-yo squat and unilateral squats on a custom-made vibration platform + 2 sets of 6-8 reps of nordic curls | 3-4 soccer practices (~6 h), 1 session of strength/power exercises, and 1 competitive match (weekend). Skill training - warmup, technical actions, small-sided games, and tactical activities  Photoelectric cells (Musclelab, Ergotest Technology, Langesund, Norway) | 0-30m performance = MD (s): -0.02; 95% CI [-0.11, 0.07]; % Change -0.44%;  SMD: -0.05; 95% CI [-0.27, 0.18] Weight 2.26%; Inference - Trivial |
| Winwood et al. (2015) B (35) | M, n=15, Mixed (Elite and Sub-elite) Rugby Players; Age 22.5±3.4 years | Strength training 2d/wk, 7wks, 14 sessions, Off-season | Moderate-heavy loads (70-85% 1RM) moderate volume 2-3 sets of 5-8 reps/ set) of clean and jerk, deadlift, military press, back squat and one arm row | 3 conditioning sessions, 3 technical sessions/wk  Electronic timing gates (Brower TC-System; Brower Timing Systems, Draper, UT, USA) | 0-30m performance = MD (s): 0.01; 95% CI [-0.03, 0.05]; % Change 0.23%;  SMD: 0.05; 95% CI [-0.15, 0.25] Weight 2.29%; Inference - Trivial |
| Wong et al. (2010) A (36) | M, n=20, Elite Soccer Players; Age 24.6±1.5 years | Strength and power training 2d/wk, 8wks, 16 sessions, Pre-season | Heavy loads (85%) and moderate volume (4 sets of 6 reps/set) of LB power + FB strength training (high pull, jump squat, half back squat, bench press, chin ups) | 6-8 soccer training sessions/wk, each lasting for 90 minutes + High intensity interval training @120% MAS 15:15 work/rest 2/wk  Infrared photoelectronic cells (Speedtrap II Wireless Timing System, Brower Timing System, Australia) | 0-30m performance ↑ MD (s): 0.12; 95% CI [0.11, 0.13]; % Change 2.8%;  SMD: 4; 95% CI [3.47, 4.53] Weight 1.86%; Inference - Large |

M = male, F = female, UB = upper body, LB = lower body, FB = full body, 1RM = one-repetition maximum BW = bodyweight, BB = barbell, KB = kettlebell, DB = dumbbell, AEL = accentuated eccentric loading, SL = single leg, COD = change of direction, CMJ = countermovement jump, N/A = data not available, SMD = standardised mean difference, CI = confidence interval, MD = mean difference, % Change = percentage change, d = day, wk(s) = week(s), hr(s) = hour(s), ↑ = significant increase in sprint performance (p = < 0.05) , = = no significant change in sprint performance (p = > 0.05), ↓ = significant decrease in sprint performance (p = < 0.05), the resistance and plyometric training intensity and volume descriptors are based descriptions from the study or previous guidelines (75-78).

# Declarations

**Ethics**

Approval was obtained from the ethics committee of Leeds Beckett University. The procedures used in this study comply with the ethical standards of the Declaration of Helsinki.

**Consent for publication**

Not applicable

**Availability of data and materials**

The datasets generated during and/or analysed during the current study are available from the corresponding author on reasonable request.

**Funding**
No sources of funding were used to assist in the preparation of this article.

**Conflicts of interest**

Ben Nicholson, Alex Dinsdale, Ben Jones and Kevin Till declare no potential conflicts of interest concerning the research, content, authorship, and/or publication of this review.

**Authors' contributions**

All the authors contributed to the manuscript, including the conception and design of the study, analysis and interpretation of the data, drafting and critically revising the manuscript, and approval for publication. All authors read and approved the final manuscript.

# References

1. Alptekin A, Kılıç Ö, Maviş M. The effect of an 8-week plyometric training program on sprint and jumping performance. Serbian J Sport Sci. 2013;7(2):45-50.

2. Borges JH, Conceição MS, Vechin FC, Pascoal EHF, Silva RP, Borin JP. The effects of resisted sprint vs. plyometric training on sprint performance and repeated sprint ability during the final weeks of the youth soccer season. Sci Sports. 2016;31(4):e101-e5.

3. Bouguezzi R, Chaabene H, Negra Y, Ramirez-Campillo R, Jlalia Z, Mkaouer B, et al. Effects of different plyometric training frequency on measures of athletic performance in prepuberal male soccer players. J Strength Cond Res. 2018.

4. Chelly MS, Ghenem MA, Abid K, Hermassi S, Tabka Z, Shephard RJ. Effects of in-season short-term plyometric training program on leg power, jump-and sprint performance of soccer players. J Strength Cond Res. 2010;24(10):2670-6.

5. Christou M, Smilios I, Sotiropoulos K, Volaklis K, Pilianidis T, Tokmakidis SP. Effects of resistance training on the physical capacities of adolescent soccer players. J Strength Cond Res. 2006;20(4):783-91.

6. Coratella G, Beato M, Milanese C, Longo S, Limonta E, Rampichini S, et al. Specific adaptations in performance and muscle architecture after weighted jump-squat vs. body mass squat jump training in recreational soccer players. J Strength Cond Res. 2019;32(4):921-9.

7. de Hoyo M, Gonzalo-Skok O, Sañudo B, Carrascal C, Plaza-Armas JR, Camacho-Candil F, et al. Comparative effects of in-season full-back squat, resisted sprint training, and plyometric training on explosive performance in U-19 elite soccer players. J Strength Cond Res. 2016;30(2):368-77.

8. Douglas J, Pearson S, Ross A, McGuigan M. Effects of accentuated eccentric loading on muscle properties, strength, power, and speed in resistance-trained rugby players. J Strength Cond Res. 2018;32(10):2750-61.

9. Escobar-Álvarez J, Fuentes-García J, Pérez-Sousa M, Calleja-González J. Effect of very heavy sled training in speed performance (30m) in female rugby union players. ICTS VI International Conference in Team Sports; España. 2018. p. 1.

10. Gabbett TJ, Johns J, Riemann M. Performance changes following training in junior rugby league players. J Strength Cond Res. 2008;22(3):910-7.

11. García-Pinillos F, Martínez-Amat A, Hita-Contreras F, Martínez-López EJ, Latorre-Román PA. Effects of a contrast training program without external load on vertical jump, kicking speed, sprint, and agility of young soccer players. J Strength Cond Res. 2014;28(9):2452-60.

12. Hammami M, Negra Y, Aouadi R, Shephard RJ, Chelly MS. Effects of an in-season plyometric training program on repeated change of direction and sprint performance in the junior soccer player. J Strength Cond Res. 2016;30(12):3312-20.

13. Hammami M, Gaamouri N, Shephard RJ, Chelly MS. Effects of contrast strength vs. plyometric training on lower limb explosive performance, ability to change direction and neuromuscular adaptation in soccer players. J Strength Cond Res. 2018;33(8):2094-103.

14. Karsten B, Larumbe-Zabala E, Kandemir G, Hazir T, Klose A, Naclerio F. The effects of a 6-week strength training on critical velocity, anaerobic running distance, 30-m sprint and Yo-Yo intermittent running test performances in male soccer players. PloS one. 2016;11(3):e0151448.

15. Krommes K, Petersen J, Nielsen MB, Aagaard P, Hölmich P, Thorborg K. Sprint and jump performance in elite male soccer players following a 10-week Nordic Hamstring exercise protocol: a randomised pilot study. BMC Res Notes. 2017;10(1):669-.

16. Loturco I, Ugrinowitsch C, Tricoli V, Pivetti B, Roschel H. Different loading schemes in power training during the preseason promote similar performance improvements in brazilian elite soccer players. J Strength Cond Res. 2013;27(7):1791-7.

17. Loturco I, Pereira LA, Kobal R, Maldonado T, Piazzi AF, Bottino A, et al. Improving sprint performance in soccer: effectiveness of jump squat and olympic push press exercises. Plos One. 2016;11(4):e0153958-e.

18. Loturco I, Kobal R, Kitamura K, Cal Abad CC, Faust B, Almeida L, et al. Mixed training methods: effects of combining resisted sprints or plyometrics with optimum power loads on sprint and agility performance in professional soccer players. Front Physiol. 2017;8:1034.

19. Manouras N, Papanikolaou Z, Karatrantou K, Kouvarakis P, Gerodimos V. The efficacy of vertical vs. horizontal plyometric training on speed, jumping performance and agility in soccer players. Int J Sports Sci Coach. 2016;11(5):702-9.

20. McMaster D, Gill N, McGuigan M, Cronin J. Effects of complex strength and ballistic training on maximum strength, sprint ability and force-velocity-power profiles of semi-professional rugby union players. J Aust Strength Cond. 2014;22(1):17-30.

21. Negra Y, Chaabene H, Hammami M, Hachana Y, Granacher URS. Effects of high-velocity resistance training on athletic performance in prepuberal male soccer athletes. J Strength Cond Res. 2016;30(12):3290-7.

22. Orange ST, Metcalfe JW, Robinson A, Applegarth MJ, Liefeith A. Effects of in-season velocity-versus percentage-based training in academy rugby league players. Int J Sports Physiol Perform. 2019;1(aop):1-8.

23. Ramírez-Campillo R, Gallardo F, Henriquez-Olguín C, Meylan CM, Martínez C, Álvarez C, et al. Effect of vertical, horizontal, and combined plyometric training on explosive, balance, and endurance performance of young soccer players. J Strength Cond Res. 2015;29(7):1784-95.

24. Ramírez-Campillo R, Burgos CH, Henríquez-Olguín C, Andrade DC, Martínez C, Álvarez C, et al. Effect of unilateral, bilateral, and combined plyometric training on explosive and endurance performance of young soccer players. J Strength Cond Res. 2015;29(5):1317-28.

25. Ramírez-Campillo R, Vergara-Pedreros M, Henríquez-Olguín C, Martínez-Salazar C, Alvarez C, Nakamura FY, et al. Effects of plyometric training on maximal-intensity exercise and endurance in male and female soccer players. J Sport Sci. 2016;34(8):687-93.

26. Ramirez-Campillo R, Álvarez C, García-Pinillos F, García-Ramos A, Loturco I, Chaabene H, et al. Effects of combined surfaces vs. single-surface plyometric training on soccer players' physical fitness. J Strength Cond Res. 2019;Publish Ahead of Print.

27. Randell AD, Cronin JB, Keogh JWL, Gill ND, Pedersen MC. Effect of instantaneous performance feedback during 6 weeks of velocity-based resistance training on sport-specific performance tests. J Strength Cond Res. 2011;25(1):87-93.

28. Rimmer E, Sleivert G. Effects of a plyometrics intervention program on sprint performance. J Strength Cond Res. 2000;14(3):295-301.

29. Ronnestad BR, Kvamme NH, Sunde A, Raastad T. Short-term effects of strength and plyometric training on sprint and jump performance in professional soccer players. J Strength Cond Res. 2008;22(3):773-80.

30. RØNnestad BR, Nymark BS, Raastad T. Effects of in-season strength maintainance training frequency in professional scocer players. J Strength Cond Res. 2011;25(10):2653-60.

31. Scott BR, Peiffer JJ, Goods PSR. The effects of supplementary low-load blood flow restriction training on morphological and performance-based adaptations in team sport athletes. J Strength Cond Res. 2017;31(8):2147-54.

32. Shalfawi SA, Ingebrigtsen J, Dillern T, Tønnessen E, Delp TK, Enoksen E. The effect of 40 m repeated sprint training on physical performance in young elite male soccer players. Serbian J Sport Sci. 2012;6(3).

33. SÖhnlein Q, MÜller E, StÖggl TL. The effect of 16-week plyometric training on explosive actions in early to mid-puberty elite soccer players. J Strength Cond Res. 2014;28(8):2105-14.

34. Tous-Fajardo J, Gonzalo-Skok O, Arjol-Serrano JL, Tesch P. Enhancing change-of-direction speed in soccer players by functional inertial eccentric overload and vibration training. Int J Sports Physiol Perform. 2016;11(1):66-73.

35. Winwood PW, Cronin JB, Posthumus LR, Finlayson SJ, Gill ND, Keogh JW. Strongman vs. traditional resistance training effects on muscular function and performance. J Strength Cond Res. 2015;29(2):429-39.

36. Wong P-l, Chaouachi A, Chamari K, Dellal A, Wisloff U. Effect of preseason concurrent muscular strength and high-intensity interval training in professional soccer players. J Strength Cond Res. 2010;24(3):653-60.
