# Supplementary material for: The Training of Medium- to Long-Distance Sprint Performance in Football Code Athletes: A Systematic Review and Meta-analysis
Source: Sports Med. 2021 Sep 9;52(2):257–86. doi: 10.1007/s40279-021-01552-4 (PMC8803780; doi:10.1007/s40279-021-01552-4)
Supplement: Supplementary file 2 — Supplementary file2 (DOCX 58 kb) [file 40279_2021_1552_MOESM2_ESM.docx]

**Electronic Supplementary Material Table S2**

Article title - The Training of Medium-Long-Sprint Performance in Football Code Athletes: A Systematic Review and Meta-Analysis

Journal name – Sports Medicine

Author names - Ben Nicholson, Alex Dinsdale, Ben Jones, and Kevin Till.

Affiliations - Leeds Beckett University, Carnegie Applied Rugby Research (CARR) centre, Carnegie School of Sport, Leeds, United Kingdom. Yorkshire Carnegie Rugby Union club, Leeds, United Kingdom. Leeds Rhinos Rugby League club, Leeds, United Kingdom. England Performance Unit, The Rugby Football League, Leeds, United Kingdom. School of Science and Technology, University of New England, Armidale, NSW, Australia. Division of Exercise Science and Sports Medicine, Department of Human Biology, Faculty of Health Sciences, the University of Cape Town and the Sports Science Institute of South Africa, Cape Town, South Africa.

corresponding author e-mail address – b.t.nicholson@leedsbeckett.ac.uk

**Table S2**

**Characteristics of the combined methods sprint training groups (primary and or secondary methods combined with tertiary methods) included in the review**

| **Study (year)** | **Subjects** | **Training type and organisation** | **Training methods and testing equipment** | **Other training** | **Mean difference, 95% CI, percentage change, Std. Mean Difference IV, Random, 95% CI, weight, Qualitative inference** |
| --- | --- | --- | --- | --- | --- |
| Barr et al. (2015) A (1) | M, n=8, Elite Rugby Sevens Players; Age 22.4±2.7 years | Strength, power, resisted and unresisted sprint training 3-4d/wk, 3wks, 9 sessions, Pre-season | Speed training (~200 m of 10-25m sprints) and lightly weighted sled-resisted sprints up to 10m. Strength training 5-6 sets of LB strength-power training (2-6 reps/set) snatch or clean and jerk variations squats or jump squats and UB strength exercises (6-8 reps/set) UB pressing and pulling exercises + training for the abdominal muscles and some individualised injury prevention exercises. Simulated hypergravity using a weighted vest (12%BW) at all times when standing apart from training. | Rugby practice 2-4 times/wk focusing on technical passing, catching and kicking drills, as well as different conditioning games that varied in numbers per team (4–7), contact (touch rugby or full tackle/ rucks), and space (full-field or half-field)  Electronic timing system (Brower, Draper, UT, USA) | >30m performance = MD (s): 0; 95% CI [-0.05, 0.05]; % Change 0%;  SMD: 0; 95% CI [-0.28, 0.28] Weight 5.54%; Inference – Trivial  Vmax = MD (s): 0; 95% CI [0, 0]; % Change 0%;  SMD: 0; 95% CI [-0.22, 0.22] Weight 11.15%; Inference - Trivial |
| Barr et al. (2015) B (1) | M, n=7, Elite Rugby Sevens Players; Age 22±2.1 years | Strength, plyometrics, resisted and unresisted sprint training 3-4d/wk, 3wks, 9 sessions, Pre-season | See Barr et al. (2015) A, matched training with no hypergravity weighted vest | See Barr et al. (2015) A | >30m performance = MD (s): 0.01; 95% CI [-0.03, 0.05]; % Change 0.19%;  SMD: 0.08; 95% CI [-0.21, 0.38] Weight 5.44%; Inference – Trivial  Vmax = MD (s): 0; 95% CI [-0.01, 0.01]; % Change 0%;  SMD: 0; 95% CI [-0.23, 0.23] Weight 11.04%; Inference - Trivial |
| Beato et al. (2018) A (2) | M, n=11, Elite Soccer Players; Age 17±0.8 years | Plyometrics and unresisted sprint training 2d/wk, 6wks, 12 sessions, In-season | 3-4 sets of 3 short shuttle runs (sprints - distance not specified) with 4 changes of direction + moderate-high intensity moderate volume (5 sets of 4 reps/set) plyometrics (drop jumps from 60 cm followed by a jump over an obstacle 15 cm height + jumps over obstacles of 15 cm height). | 4 training sessions/wk as team practices and an official match every Saturday  Infrared timing gates (Microgate, Bolzano, Italy) | 0-30m performance ↑ MD (s): 0.05; 95% CI [0.01, 0.09]; % Change 1.18%;  SMD: 0.32; 95% CI [0.07, 0.56] Weight 4.43%; Inference – Small  >30m performance ↑ MD (s): 0.08; 95% CI [0.02, 0.14]; % Change 1.48%;  SMD: 0.31; 95% CI [0.07, 0.55] Weight 5.71%; Inference - Small |
| Bianchi et al. (2019) A (3) | M, n=10, Elite Soccer Players; Age 17±0.8 years | Strength, plyometrics and unresisted sprint training 1d/wk, 8wks, 8 sessions, In-season | 4 Sets of 5-6 foot contacts/set of plyometric training using drop jumps (60cm) and horizontal jumps both followed by jumps over 2 obstacles (15cm) + strength and speed training (long and short) not specified | 4 training sessions/wk as team practices and an official match every Saturday + 3 sets of 3 short shuttle runs with 4 COD each, for an amount of 36 COD  Infrared timing gates (Microgate, Bolzano, Italy) | 0-30m performance ↑ MD (s): 0.06; 95% CI [0.02, 0.1]; % Change 1.43%;  SMD: 0.4; 95% CI [0.14, 0.66] Weight 4.4%; Inference – Moderate  >30m performance ↑ MD (s): 0.21; 95% CI [0.14, 0.28]; % Change 3.98%;  SMD: 0.79; 95% CI [0.51, 1.08] Weight 5.51%; Inference - Moderate |
| Bianchi et al. (2019) B (3) | M, n=11, Elite Soccer Players; Age 17±0.8 years | Strength, plyometrics and unresisted sprint training 2d/wk, 8wks, 16 sessions, In-season | 4 Sets of 5-6 foot contacts/set of plyometric training using drop jumps (60cm) and horizontal jumps both followed by jumps over 2 obstacles (15cm) + strength and speed training (long and short) not specified | See Bianchi et al. (2019) A | 0-30m performance ↑ MD (s): 0.1; 95% CI [0.06, 0.14]; % Change 2.35%;  SMD: 0.64; 95% CI [0.38, 0.9] Weight 4.39%; Inference – Moderate  >30m performance ↑ MD (s): 0.06; 95% CI [0.02, 0.1]; % Change 1.1%;  SMD: 0.34; 95% CI [0.1, 0.58] Weight 5.7%; Inference - Small |
| Cook et al. (2013) A (4) | M, n=20, Elite Rugby union Players; Age 19.7±0.7 years | Strength, power and unresisted sprint training 2d/wk, 3wks, 6 sessions, Pre-season | LB strength and power training and UB strength training performed as separate sessions. 4 sets of 5 reps/set high load (80% 1RM) strength training each set consisting of momentary muscular failure. LB = squats, mid-thigh pulls and Romanian deadlifts + CMJs (7 sets of 1 reps per /set). UB = bench press, weighted pull-ups, and single-arm dumbbell bent-over rows. + 1 set of 5 reps of medium distance sprints (40m) | 2 extra sessions/wk 1 skill session incorporating a game of touch rugby, and 1 endurance session  Electronic timing light gates (Brower Timing System, Salt Lake City, UT, USA) | >30m performance = MD (s): 0.01; 95% CI [-0.04, 0.06]; % Change 0.33%;  SMD: 0.03; 95% CI [-0.14, 0.21] Weight 5.98%; Inference - Trivial |
| Cook et al. (2013) B (4) | M, n=20, Elite Rugby union Players; Age 19.7±0.7 years | Strength, power and unresisted sprint training 2d/wk, 3wks, 6 sessions, Pre-season | LB strength and power training and UB strength training performed as separate sessions. 4 sets of 5 reps/set of Supra maximal loads (120% 1RM) eccentric strength training. LB = squats, mid-thigh pulls and Romanian deadlifts + CMJs (7 sets of 1 reps per /set). UB = bench press, weighted pull-ups, and single-arm dumbbell bent-over rows. + 1 set of 5 reps of medium distance sprints (40m) | See Cook et al. (2013) A | >30m performance = MD (s): 0; 95% CI [-0.05, 0.05]; % Change -0.01%;  SMD: 0; 95% CI [-0.18, 0.18] Weight 5.98%; Inference - Trivial |
| Cook et al. (2013) C (4) | M, n=20, Elite Rugby union Players; Age 19.7±0.7 years | Strength, power and assisted sprint training 2d/wk, 3wks, 6 sessions, Pre-season | LB strength and power training and UB strength training performed as separate sessions. 4 sets of 5 reps/set high load (80% 1RM) strength training each set consisting of momentary muscular failure. LB = squats, mid-thigh pulls and Romanian deadlifts + CMJs (7 sets of 1 reps per /set). UB = bench press, weighted pull-ups, and single-arm dumbbell bent-over rows. + 1 set of 5 reps of medium distance sprints (40m) with 25m/rep over speed (2% decline) | See Cook et al. (2013) A | >30m performance = MD (s): 0.04; 95% CI [-0.01, 0.09]; % Change 0.73%;  SMD: 0.14; 95% CI [-0.04, 0.32] Weight 5.98%; Inference - Trivial |
| Cook et al. (2013) D (4) | M, n=20, Elite Rugby union Players; Age 19.7±0.7 years | Strength, power and assisted sprint training 2d/wk, 3wks, 6 sessions, Pre-season | LB strength and power training and UB strength training performed as separate sessions. 4 sets of 5 reps/set of Supra maximal loads (120% 1RM) eccentric strength training. LB = squats, mid-thigh pulls and Romanian deadlifts + CMJs (7 sets of 1 reps per /set). UB = bench press, weighted pull-ups, and single-arm dumbbell bent-over rows. + 1 set of 5 reps of medium distance sprints (40m) with 25m/rep over speed (2% decline) | See Cook et al. (2013) A | >30m performance = MD (s): 0.03; 95% CI [-0.02, 0.08]; % Change 0.59%;  SMD: 0.1; 95% CI [-0.07, 0.28] Weight 5.98%; Inference - Trivial |
| Coutts et al. (2007) A (5) | M, n=7, Elite Rugby league Players; Age 25.7±2.6 years | Strength, power and unresisted sprint training 2-3d/wk, 6wks, 18 sessions, Pre-season | High load (78.5-86%1RM) strength and power training (3-4 sets of 5-8 reps/set). LB: prone hamstring flicks, box jumps (40 cm), hang clean, back squat, deadlift, BB step-ups (40 cm) and hamiglut raise. UB: internal/external shoulder, bench throw, push press, underhand weighted chin-ups, DB incline bench press, front military press, abdominal circuit + speed/agility training (not specified) | 5-7 sessions per week of field-based specific rugby league training, aerobic endurance development, skill and speed/agility training  Electronic timing gates (Swift, Lismore, Australia) | >30m performance = MD (s): -0.02; 95% CI [-0.08, 0.04]; % Change -0.37%;  SMD: -0.11; 95% CI [-0.4, 0.19] Weight 5.44%; Inference - Trivial |
| De Hoyo et al. (2016) C (6) | M, n=9, Elite Soccer Players; Age 18±1 years | Plyometrics and unresisted sprint training 2d/wk, 8wks, 16 sessions, In-season | Low-high intensity plyometrics 1-3 sets of 8-10 reps/set combined with short sprints (10-15m sprints). The exercises consisted of unilateral crossing jumps + 15-m sprint, lunges + 3m zigzag + 10-m sprint, unilateral alternative jumps + 15-m sprint, unilateral lateral jumps (40 cm hurdle) + 5-m zigzag + 10-m sprint, (e) speed ladder exercises, headers + 5-m sprint + deceleration + 2-m back running + 10-m sprint, double lateral jumps (20 cm hurdle) + zigzag + 10-m sprint, and unilateral lateral jumps + shooting without controlling the ball on an artificial grass surface. | 10 hrs of combined soccer (4-5 sessions) and conditioning (1 session) training, and 1 competitive match/wk.  Dual-beam electronic timing gate OptoJump System (Polifemo Radio Light, Microgate, Bolzano, Italy) | 0-30m performance = MD (s): 0.04; 95% CI [-0.01, 0.09]; % Change 0.97%;  SMD: 0.2; 95% CI [-0.07, 0.46] Weight 4.38%; Inference – Small  >30m performance ↑ MD (s): 0.09; 95% CI [0.02, 0.16]; % Change 1.39%;  SMD: 0.35; 95% CI [0.08, 0.62] Weight 5.58%; Inference - Small |
| Enoksen et al. (2013) A (7) | M, n=9, Elite Soccer Players; Age 19±3.5 years | Strength, power and unresisted sprint training 2d/wk, 10wks, 20 sessions, Pre-season | Supervised hypertrophy training (6wk) moderate-high load (70-85%1RM) high volume (3 sets of 8-12 reps/set) followed by strength/ power training (4wk) and 2 sets of 40m sprints | 1.5 hrs soccer training during the intervention  Start mat and photocells | >30m performance = MD (s): 0.06; 95% CI [0, 0.12]; % Change 1.15%;  SMD: 0.25; 95% CI [-0.01, 0.52] Weight 5.6%; Inference - Small |
| Enoksen et al. (2013) B (7) | M, n=8, Elite Soccer Players; Age 19±3.5 years | Strength, power and unresisted sprint training 2d/wk, 10wks, 20 sessions, Pre-season | Unsupervised hypertrophy training (6wk) moderate-high load (70-85%1RM) high volume (3 sets of 8-12 reps/set) followed by strength/ power training (4wk) and 2 sets of 40m sprints | See Enoksen et al. (2013) A | >30m performance = MD (s): -0.02; 95% CI [-0.06, 0.02]; % Change -0.39%;  SMD: -0.15; 95% CI [-0.43, 0.12] Weight 5.53%; Inference - Trivial |
| Faude et al. (2013) A (8) | M, n=8, Sub-elite Soccer Players; Age 23.1±2.7 years | Strength, power and unresisted sprint training 2d/wk, 7wks, 14 sessions, In-season | Day1: High load (90%1RM) unilateral half squats (4 sets of 4 reps/set) combined with single leg hurdle jumps (4 sets of 5 reps/set). Day 2: exercises performed as a tri-set. 2-3 sets of 4-5 reps/set moderate load (50–60% 1RM) LB power exercises (half squats, calf raises, lateral half squats, step-ups) combined with plyometric exercises (drop jumps, high straight jumps, lateral jumps, bounding) and 2-3 sets of headers (3 reps/set) or short sprints 5m sprints or zigzag sprints (1 rep/set) | 4 soccer training sessions/wk and 1 competitive match at the weekend.  Single beam electronic timing gates (Racetime 2, Microgate, Bolzano, Italy) | 0-30m performance = MD (s): -0.01; 95% CI [-0.06, 0.04]; % Change -0.23%;  SMD: -0.05; 95% CI [-0.33, 0.22] Weight 4.36%; Inference - Trivial |
| Gil et al. (2018) A (9) | M, n=9, Elite Soccer Players; Age 22.8±4.3 years | Power and unresisted sprint training 2d/wk, 6wks, 12 sessions, Pre-season | Moderate load (60%1RM) low volume 4-6 sets of 6 reps/set of squat jumps + 2-4 sets of 4 reps/set of unresisted short sprints (7m) and 2-4 sets of 4 reps/set of change of direction speed exercises consisting of short sprint shuttles and short sprints followed by a 45 degrees COD. | 3-4d/wk technical/tactical  Photocells (Smartspeed System, Fusion Sport, Australia | 0-30m performance ↑ MD (s): 0.07; 95% CI [0.02, 0.12]; % Change 1.97%;  SMD: 0.36; 95% CI [0.09, 0.63] Weight 4.37%; Inference - Small |
| Gil et al. (2018) B (9) | M, n=9, Elite Soccer Players; Age 22±2.2 years | Power and resisted sprint training 2d/wk, 6wks, 12 sessions, Pre-season | Moderate load (60%1RM) low volume 4-6 sets of 6 reps/set of squat jumps + 2-4 sets of 4 reps/set of resisted (load @ -10% Vmax) short sprints (7m) and 2-4 sets of 4 reps/set of change of direction speed exercises consisting of short sprint shuttles and short sprints followed by a 45 degrees COD. | See Gil et al. (2018) A | 0-30m performance ↑ MD (s): 0.13; 95% CI [0.1, 0.16]; % Change 3.64%;  SMD: 1.27; 95% CI [0.92, 1.62] Weight 4.19%; Inference - Large |
| Hammami et al. (2016) 2A (10) | M, n=12, Elite Soccer Players; Age 12.5±0.3 years | Strength and plyometrics training 2d/wk, 8wks, 16 sessions, In-season | 4wks plyometrics training followed by a 4wks balance training. Plyometric drills included CMJs, drop jumps +1 step, horizontal line jump, lateral hops, ankle jumps, single leg cone hops, hurdle jumps, single leg maximal rebound hops +5m acceleration 1-3 sets of 8-15 foot contacts/set. Balance training consisted of 1-3 sets of 30-45s/set kneeling swiss ball balancing, 8-15 reps/set of squats, single leg straight leg bridges and lunges performed on an unstable surface e.g., foam surfaces, bosu balls or inflated disks. | None were involved in any after-school activities or any formalised strength and conditioning training programs.  Photocell gates (Brower Timing Systems, Salt Lake City, Utah, USA) | 0-30m performance ↑ MD (s): 0.1; 95% CI [0.05, 0.15]; % Change 2%;  SMD: 0.5; 95% CI [0.26, 0.74] Weight 4.43%; Inference – Moderate  Vmax ↓ MD (s): -0.1; 95% CI [-0.17, -0.03]; % Change -3.33%;  SMD: -0.25; 95% CI [-0.43, -0.07] Weight 11.38%; Inference - Small |
| Hammami et al. (2016) 2B (10) | M, n=12, Elite Soccer Players; Age 12.7±0.3 years | Strength and plyometrics training 2d/wk, 8wks, 16 sessions, In-season | 4wks balance training followed by a 4wks plyometrics training. Balance training consisted of 1-3 sets of 30-45s/set kneeling swiss ball balancing, 8-15 reps/set of squats, single leg straight leg bridges and lunges performed on an unstable surface e.g., foam surfaces, bosu balls or inflated disks. Plyometric drills included CMJs, drop jumps +1 step, horizontal line jump, lateral hops, ankle jumps, single leg cone hops, hurdle jumps, single leg maximal rebound hops +5m acceleration 1-3 sets of 8-15 foot contacts/set | See Hammami et al. (2016) 2A | 0-30m performance ↑ MD (s): 0.1; 95% CI [0.02, 0.18]; % Change 2%;  SMD: 0.29; 95% CI [0.05, 0.52] Weight 4.45%; Inference – Small  Vmax ↓ MD (s): -0.1; 95% CI [-0.17, -0.03]; % Change -3.23%;  SMD: -0.25; 95% CI [-0.43, -0.07] Weight 11.38%; Inference - Small |
| Hammami et al. (2018) A (11) | M, n=14, Elite Soccer Players; Age 16.1±0.5 years | Strength, plyometrics and unresisted sprint training 2d/wk, 8wks, 16 sessions, In-season | Moderate-high load (70-90% 1RM) moderate volume (3-5 sets of 3-8 reps/set) strength training (half squat) + low volume (3-5 sets of 1-3 foot contacts) low intensity plyometrics training (CMJ) + short sprints (15m) 3-5 sets of 1 rep/set (wk5-8 only) | Soccer training 4-5 times/wk and 1 official game/wk  Paired photocell timers (Microgate, Bolzano, Italy) | >30m performance ↑ MD (s): 0.5; 95% CI [0.45, 0.55]; % Change 9.21%;  SMD: 2.17; 95% CI [1.79, 2.56] Weight 4.98%; Inference - Large |
| Harris et al. (2008) A (12) | M, n=7, Elite Rugby league Players; Age 21.8±4 years | Strength and unresisted sprint training 1-2d/wk, 7wks, 13 sessions, Pre-season | High loads hack squat machine squat jumps at 80% 1RM for 5 sets of 5 reps/set + other lower-body exercises at various loads, and UB training and sprint drills 2 times/wk (not specified) | N/A  Kinematic Measurement System (KMS, Optimal Kinetics, Ind) | 0-30m performance ↑ MD (s): 0.07; 95% CI [0.03, 0.11]; % Change 1.7%;  SMD: 0.58; 95% CI [0.26, 0.9] Weight 4.26%; Inference - Moderate |
| Harris et al. (2008) B (12) | M, n=8, Elite Rugby league Players; Age 21.8±4 years | Power and unresisted sprint training 1-2d/wk, 7wks, 13 sessions, Pre-season | Individually determined Pmax loads (20.0-43.5% 1RM) machine squat jumps for 6 sets of 10-12 reps/set + other lower-body exercises at various loads, and upper-body training and sprint drills 2 times/wk (not specified) | See Harris et al. (2008) A | 0-30m performance = MD (s): 0.05; 95% CI [0, 0.1]; % Change 1.2%;  SMD: 0.27; 95% CI [-0.02, 0.55] Weight 4.35%; Inference - Small |
| Lahti et al. (2019) A (13) | M, n=10, Elite Soccer Players; Age 20.75±3.15 years | Strength, resisted and unresisted sprint training 1-2d/wk, 9wks, 14 sessions, Pre-season | 2-6 sprints/set, 2-5 reps of short distance (15m) resisted sprints (-60% Vmax) and 1-2 short unresisted sprints (20m) and strength training 2d/wk (not specified) | 6-10 sessions/wk + on average 1 official game  Radar device (Stalker ATS Pro II, Applied Concepts, TX, USA) | 0-30m performance ↑ MD (s): 0.09; 95% CI [0.05, 0.13]; % Change 1.97%;  SMD: 0.52; 95% CI [0.26, 0.79] Weight 4.38%; Inference - Moderate |
| Lahti et al. (2019) B (13) | M, n=9, Elite Soccer Players; Age 25.22±6.86 years | Strength, resisted and unresisted sprint training 1-2d/wk, 9wks, 14 sessions, Pre-season | 2-6 sprints/set, 2-5 reps of short distance (20m) resisted sprints (-50% Vmax) and 1-2 short unresisted sprints (20m) and strength training 2d/wk (not specified) | See Lahti et al. (2019) A | 0-30m performance ↑ MD (s): 0.13; 95% CI [0.1, 0.16]; % Change 2.9%;  SMD: 1.08; 95% CI [0.75, 1.41] Weight 4.24%; Inference - Large |
| Lopez-Segovia et al. (2010) A (14) | M, n=19, Elite Soccer Players; Age 18.43±0.6 years | Power, plyometrics and resisted sprint training 1-2d/wk, 15wks, 22 sessions, In-season | Velocity-based strength training (0.8-1.2 m/s full squat, +10-20% of full squat load for the half squat @ 1 m/s) 2-8 sets of 4 reps/set (full squat and half squat) + power and plyometric training 1-4 sets of 4-5 reps/set (loaded CMJ corresponding to a 20cm jump (40-70% load), CMJ, step phase triple jumps, displacements with loads + 4-5 sets of 1 rep/set of resisted 20-25m sled towing (10kg) and 3-6 sets of 1 rep/set of short sprints (20m) | 4 soccer field sessions/wk  Photoelectric cell barriers | 0-30m performance ↓ MD (s): -0.1; 95% CI [-0.14, -0.06]; % Change -2.3%;  SMD: -0.51; 95% CI [-0.7, -0.32] Weight 4.51%; Inference – Moderate  Vmax ↓ MD (s): -0.03; 95% CI [-0.04, -0.02]; % Change -2.54%;  SMD: -0.6; 95% CI [-0.75, -0.45] Weight 11.53%; Inference - Moderate |
| Loturco et al. (2017) A (15) | M, n=7, Elite Soccer Players; Age 21.7±2.4 years | Power and resisted sprint training 2-3d/wk, 5wks, 12 sessions, Pre-season | 6 sets of 4-8 reps/set of jump squats performed @ a load corresponding to the mass at which optimal power is produced (1-1.1* optimal power load) + 1 set of 8-6 reps/set of short-medium distance resisted sprints (20-30m @ 20-5%BM) | Pre-season soccer training 6/week + 2 pre-season friendly games  Photocells (Smart Speed, Fusion Equipment, AUS) | 0-30m performance ↑ MD (s): 0.07; 95% CI [0.05, 0.09]; % Change 1.7%;  SMD: 0.9; 95% CI [0.55, 1.26] Weight 4.19%; Inference - Large |
| Michailidis et al. (2019) A (16) | M, n=17, Sub-elite Soccer Players; Age 11.8±0.8 years | 4 sets of 5-10 foot contacts/set of unilateral and bilateral vertical, lateral and horizontal jumps + speed and sprint workload (not specified) | Soccer training 3d/wk + 1 match/wk training session included technical skills, tactics and small-sided games Infrared photoelectric gates (Microgate, Bolzano, Italy) | Soccer training 3d/wk + 1 match/wk training session included technical skills, tactics and small-sided games | 0-30m performance ↓ MD (s): -0.2; 95% CI [-0.32, -0.08]; % Change -3.45%;  SMD: -0.33; 95% CI [-0.52, -0.13] Weight 4.51%; Inference - Small |
| Ozbar (2015) A (17) | F, n=10 Elite Soccer Players; Age 19.4±1.6 years | Plyometrics and unresisted sprint training 2d/wk, 10wks, 20 sessions, Phase not reported | Low-high intensity plyometrics training 3-5 sets of 5-8 foot contacts/set (horizontal, lateral, diagonal hurdle jumps, cone hops, lateral cone hops/jumps, standing long jumps, skipping, single leg jumps, split squat jumps, vertical, lateral and horizontal jumps, jumps with 180 degree turns  combined with short distance sprints 1 set of 3-5 reps/set (5m) from various start positions/ actions | Technical and tactical training (4d/wk) and preparation matches 2x/wk  Infrared photoelectric cells (Newtest 2000 Sprint Timing System, Oulu, Finland) | 0-30m performance ↑ MD (s): 0.5; 95% CI [0.36, 0.64]; % Change 10.42%;  SMD: 0.87; 95% CI [0.58, 1.16] Weight 4.33%; Inference - Large |
| Rey et al. (2017) A (18) | M, n=10, Sub-elite Soccer Players; Age 23.6±2.7 years | Resisted sprint training 2d/wk, 6wks, 12 sessions, In-season | Sprinting wearing a weighted vest @18.9% ± 2.1% of BM (1-4 sets of 3-7 sprints/set) of short sprints (20m). | 4x/wk soccer sessions containing low-intensity aerobic training, agility, and soccer-specific drills + 1 game/wk  Dual infrared reflex photoelectric cell system (DSD Laser System; Leo´ n, Spain) | 0-30m performance ↑ MD (s): 0.26; 95% CI [0.23, 0.29]; % Change 6.37%;  SMD: 2.31; 95% CI [1.84, 2.79] Weight 3.85%; Inference - Large |
| Rey et al. (2017) B (18) | M, n=9, Sub-elite Soccer Players; Age 23.7±2.1 years | Unresisted sprint training 2d/wk, 6wks, 12 sessions, In-season | Unresisted sprinting (1-4 sets of 3-7 sprints/set) of short sprints (20m). | See Rey et al. (2017) A | 0-30m performance ↑ MD (s): 0.22; 95% CI [0.15, 0.29]; % Change 5.43%;  SMD: 0.81; 95% CI [0.51, 1.11] Weight 4.3%; Inference - Large |
| Ross et al. (2015) A (19) | M, n=5, Elite Rugby Sevens Players; Age 20±1 years | Strength, power and resisted sprint training 2d/wk, 4wks, 8 sessions, In-season | High-force group session 1: Short resisted sprints (2-3 sets of 4-5 reps/set) 10 m sled sprint (30 kg) and heavy sled push (10 m)(3 sets of 3-4 reps/set) + ballistic med ball throws (3 sets of 3-4 reps/set) reverse overhead medicine ball throw @12kg). Session 2: Moderate-heavy load low volume strength speed/strength exercises (4 sets of 3/4 reps/set Countermovement squat jump @55-65%1RM, band resisted horizontal jump, ½ Squat@75-90%1RM) | In addition to their respective training program, all players also performed two UB strength sessions (65-85% 1RM, 3-5 exercises, 4 sets of 4-6 reps/set), two 60-min rugby skills sessions, and one 45-minute, field-based aerobic conditioning session.  Timing lights (Smart Speed, Swift Performance Equipment, AUS) | >30m performance = MD (s): 0.02; 95% CI [-0.06, 0.1]; % Change 0.39%;  SMD: 0.09; 95% CI [-0.26, 0.44] Weight 5.16%; Inference - Trivial  Vmax = MD (m·s^-1^): 0.04; 95% CI [-0.14, 0.22]; % Change 0.45%;  SMD: 0.06; 95% CI [-0.21, 0.34] Weight 10.72%; Inference - Trivial |
| Ross et al. (2015) B (19) | M, n=5, Elite Rugby Sevens Players; Age 21±2 years | Power, plyometrics and resisted sprint training 2d/wk, 4wks, 8 sessions, In-season | High-velocity group session 1: Medium length 10m flying sprint (20 m build up) (2-3 sets of 4-5 reps/set) 3-hurdle bound (60-75 cm) (3 sets of 3-4 reps/set) + ballistic medball throws (3 sets of 3-4 reps/set) reverse overhead medicine ball throw @3kg). Session 2: Moderate-light to moderate load low volume strength/speed exercises (4 sets of 3/4 reps/set Countermovement squat jump @20-30%1RM, horizontal drop jump, speed squat @50-60%1RM) | See Ross et al. (2015) A | >30m performance ↑ MD (s): 0.11; 95% CI [0.03, 0.19]; % Change 2.19%;  SMD: 0.51; 95% CI [0.14, 0.88] Weight 5.04%; Inference - Moderate  Vmax ↑ MD (m·s^-1^): 0.25; 95% CI [0.04, 0.46]; % Change 2.74%;  SMD: 0.33; 95% CI [0.04, 0.61] Weight 10.66%; Inference - Small |
| Shalfawi et al. (2012) A (20) | M, n=8, Elite Soccer Players; Age 16.3±0.5 years | Strength and unresisted sprint training 2d/wk, 8wks, 16 sessions, Phase not reported | Unresisted maximal sprinting (4 sets of 5 sprints/set) of medium sprints (40m). Sprint training additional to typical soccer training. + Resistance training Nordic curls, balance training (ankle strength on balance board), sit-ups, the plank, push-ups and the alternating back and arm raise 2 x/wk during soccer training (volume/ intensity not provided). | Soccer training 4 times/wk  Newtest Powertimer 300s infrared photocells | >30m performance ↑ MD (s): 0.33; 95% CI [0.24, 0.42]; % Change 6.01%;  SMD: 1.05; 95% CI [0.7, 1.39] Weight 5.19%; Inference – Large  Vmax ↑ MD (s): 0.14; 95% CI [0.1, 0.18]; % Change 5.86%;  SMD: 0.86; 95% CI [0.61, 1.12] Weight 10.88%; Inference - Large |
| Tønnessen et al. (2011) A (21) | M, n=10, Elite Soccer Players; Age 16.4±0.9 years | Strength and unresisted sprint training 1d/wk, 10wks, 10 sessions, Pre-season | Unresisted sprint training 2-4 sets of 4-5 reps of moderate distance sprints (40m) @ 95-100% max effort + Nordics curls 2-4 sets of 10 reps/set | Soccer training 3-4 times/wk. Consisting of defence tactical drills, attack tactical drills and small and large sided games.  Photo cells | >30m performance ↑ MD (s): 0.06; 95% CI [0.01, 0.11]; % Change 1.17%;  SMD: 0.29; 95% CI [0.03, 0.54] Weight 5.66%; Inference – Small  Vmax ↑ MD (s): 0.05; 95% CI [0.02, 0.08]; % Change 2.15%;  SMD: 0.37; 95% CI [0.17, 0.57] Weight 11.25%; Inference - Small |
| Tous-Fajardo et al. (2016) B (22) | M, n=12, Soccer Players; Age 17±0.5 years | Strength, plyometrics and unresisted sprint training 1d/wk, 11wks, 11 sessions, In-season | Exercises performed in a contrast tri-set format (strength exercises, plyometrics, sprinting/header simulations). Low-moderate load (50-100% body mass) moderate volume (2 sets of 6-10 reps/set) resistance training exercises (lunges, half squats, calf raises). Low-moderate intensity moderate volume plyometrics training (skipping, CMJs, reactive calf jumps, jumps to header the ball) 2 sets of 6-10 reps/set and short distance sprints (10m) 2 sets of 2 reps/set. | 3-4 soccer practices (~6 h), 1x session of strength/power exercises, and 1x competitive match (weekend). Skill training - warmup, technical actions, small-sided games, and tactical activities  Photoelectric cells (Musclelab, Ergotest Technology, Langesund, Norway) | 0-30m performance ↓ MD (s): -0.3; 95% CI [-0.38, -0.22]; % Change -6.16%;  SMD: -0.86; 95% CI [-1.12, -0.59] Weight 4.38%; Inference - Large |
| West et al. (2013) A (23) | M, n=10, Elite Rugby union Players; Age 26.8±3 years | Strength, resisted and unresisted sprint training 2d/wk, 6wks, 12 sessions, Pre-season | Maximal sprinting (2 sets of 6 sprints/set) + sled towing sprints (2 sets of 6 sprints/set) of short sprints (20m). Resistance load (12.6%Bw) on a rubber crumb surface + 3 resistance training sessions 1 UB, 1 LB, and 1 FB (not specified) | 3 conditioning sessions, 3 technical sessions/wk  Electronic timing gates (Brower TC-System; Brower Timing Systems, Draper, UT, USA) | 0-30m performance ↑ MD (s): 0.11; 95% CI [0.04, 0.18]; % Change 2.65%;  SMD: 0.39; 95% CI [0.14, 0.65] Weight 4.4%; Inference - Small |
| West et al. (2013) B (23) | M, n=10, Elite Rugby union Players; Age 25.1±3.2 years | Strength and unresisted sprint training 2d/wk, 6wks, 12 sessions, Pre-season | Maximal sprinting (4 sets of 6 sprints/set) of short sprints (20m) on a rubber crumb surface + 3 resistance training sessions 1 UB, 1 LB, and 1 FB (not specified). | See West et al. (2013) A | 0-30m performance = MD (s): 0.04; 95% CI [-0.01, 0.09]; % Change 0.96%;  SMD: 0.21; 95% CI [-0.04, 0.47] Weight 4.41%; Inference - Small |
| Winwood et al. (2015) A (24) | M, n=15, Mixed (Elite and Sub-elite) Rugby Players; Age 23.4±5.6 years | Strength training 2d/wk, 7wks, 14 sessions, Off-season | Moderate-heavy loads (70-85% 1RM) moderate volume 2-3 sets of 5-8 reps/ set) of log lift, 28m farmers carry, axle press heavy sled pull 25m and arm over arm prowler pull | 2 sessions of prehabilition exercises/wk and 2 cardiovascular training sessions focused on improving aerobic capacity  Wireless dual beam timing lights (Swift Performance Equipment). | 0-30m performance = MD (s): 0.03; 95% CI [-0.02, 0.08]; % Change 0.69%;  SMD: 0.13; 95% CI [-0.07, 0.34] Weight 4.5%; Inference - Trivial |

M = male, F = female, UB = upper body, LB = lower body, FB = full body, 1RM = one-repetition maximum BW = bodyweight, BB = barbell, KB = kettlebell, DB = dumbbell, AEL = accentuated eccentric loading, SL = single leg, PHV = peak height velocity, COD = change of direction, CMJ = countermovement jump, SMD = standardised mean difference, CI = confidence interval, MD = mean difference, % Change = percentage change, d = day, wk(s) = week(s), hr(s) = hour(s), ↑ = significant increase in sprint performance (p = < 0.05) , = = no significant change in sprint performance (p = > 0.05), ↓ = significant decrease in sprint performance (p = < 0.05), short sprints = 0-≤20m, medium sprints = 0-≤40m, long sprints 0->40m, the resistance and plyometric training intensity and volume descriptors are based descriptions from the study or previous guidelines (75-78)

# Declarations

**Ethics**

Approval was obtained from the ethics committee of Leeds Beckett University. The procedures used in this study comply with the ethical standards of the Declaration of Helsinki.

**Consent for publication**

Not applicable

**Availability of data and materials**

The datasets generated during and/or analysed during the current study are available from the corresponding author on reasonable request.

**Funding**
No sources of funding were used to assist in the preparation of this article.

**Conflicts of interest**

Ben Nicholson, Alex Dinsdale, Ben Jones and Kevin Till declare no potential conflicts of interest concerning the research, content, authorship, and/or publication of this review.

**Authors' contributions**

All the authors contributed to the manuscript, including the conception and design of the study, analysis and interpretation of the data, drafting and critically revising the manuscript, and approval for publication. All authors read and approved the final manuscript.

# References

1. Barr MJ, Gabbett TJ, Newton RU, Sheppard JM. Effect of 8 days of a hypergravity condition on the sprinting speed and lower-body power of elite rugby players. J Strength Cond Res. 2015;29(3):722-9.

2. Beato M, Bianchi M, Coratella G, Merlini M, Drust B. Effects of plyometric and directional training on speed and jump performance in elite youth soccer players. J Strength Cond Res. 2018;32(2):289-96.

3. Bianchi M, Coratella G, Dello IA, Beato M. Comparative effects of single vs. double weekly plyometric training sessions on jump, sprint and change of directions abilities of elite youth football players. J Sports Med Phys Fit. 2019;59(6):910.

4. Cook CJ, Beaven CM, Kilduff LP. Three weeks of eccentric training combined with overspeed exercises enhances power and running speed performance gains in trained athletes. J Strength Cond Res. 2013;27(5):1280-6.

5. Coutts A, Reaburn P, Piva TJ, Murphy A. Changes in selected biochemical, muscular strength, power, and endurance measures during deliberate overreaching and tapering in rugby league players. Int J Sports Med. 2007;28(2):116-24.

6. de Hoyo M, Gonzalo-Skok O, Sañudo B, Carrascal C, Plaza-Armas JR, Camacho-Candil F, et al. Comparative effects of in-season full-back squat, resisted sprint training, and plyometric training on explosive performance in U-19 elite soccer players. J Strength Cond Res. 2016;30(2):368-77.

7. Enoksen E, Staxrud M, Tønnessen E, Shalfawi S. The effect of supervised strength training on young elite male soccer players’ physical performance. Serbian J Sports Sci. 2013;7(4):173-9.

8. Faude O, Roth R, Di Giovine D, Zahner L, Donath L. Combined strength and power training in high-level amateur football during the competitive season: a randomised-controlled trial. J Sport Sci. 2013;31(13):1460-7.

9. Gil S, Barroso R, Crivoi do Carmo E, Loturco I, Kobal R, Tricoli V, et al. Effects of resisted sprint training on sprinting ability and change of direction speed in professional soccer players. J Sport Sci. 2018;36(17):1923-9.

10. Hammami R, Granacher URS, Makhlouf I, Behm DG, Chaouachi A. Sequencing effects of balance and plyometric training on physical performance in youth soccer athletes. J Strength Cond Res. 2016;30(12):3278-89.

11. Hammami M, Gaamouri N, Shephard RJ, Chelly MS. Effects of contrast strength vs. plyometric training on lower limb explosive performance, ability to change direction and neuromuscular adaptation in soccer players. J Strength Cond Res. 2018;33(8):2094-103.

12. Harris NK, Cronin JB, Hopkins WG, Hansen KT. Squat jump training at maximal power loads vs. heavy loads: effect on sprint ability. J Strength Cond Res. 2008;22(6):1742-9.

13. Lahti J, Huuhka T, Romero V, Bezodis I, Morin J, Hakkinen K. Changes in sprint performance and sagittal plane kinematics after heavy resisted sprint training in professional soccer players [Pre-print]. https://osf.io/preprints/sportrxiv/neh5m/2019 [updated 27/08/2020. 2019-05-09:[

14. López-Segovia M, Andrés JMP, González-Badillo JJ. Effect of 4 months of training on aerobic power, strength, and acceleration in two under-19 soccer teams. J Strength Cond Res. 2010;24(10):2705-14.

15. Loturco I, Kobal R, Kitamura K, Cal Abad CC, Faust B, Almeida L, et al. Mixed training methods: effects of combining resisted sprints or plyometrics with optimum power loads on sprint and agility performance in professional soccer players. Front Physiol. 2017;8:1034.

16. Michailidis Y, Tabouris A, Metaxas T. Effects of plyometric and directional training on physical fitness parameters in youth soccer players. Int J Sports Physiol Perform. 2019;14(3):392-8.

17. Ozbar N. Effects of plyometric training on explosive strength, speed and kicking speed in female soccer players. Anthropol. 2015;19(2):333-9.

18. Rey E, PadrÓN-Cabo A, FernÁNdez-Penedo D. Effects of sprint training with and without weighted vest on speed and repeated sprint ability in male soccer players. J Strength Cond Res. 2017;31(10):2659-66.

19. Ross A, Gill N, Cronin J, Cross M. The effects of two power training programmes on the sprint speed, mechanical sprint characteristics, and lower body power of rugby sevens players [Doctor of Philosophy (PhD)]. New Zealand: Auckland University of Technology; 2015.

20. Shalfawi SA, Ingebrigtsen J, Dillern T, Tønnessen E, Delp TK, Enoksen E. The effect of 40 m repeated sprint training on physical performance in young elite male soccer players. Serbian J Sport Sci. 2012;6(3).

21. Tønnessen E, Shalfawi SA, Haugen T, Enoksen E. The effect of 40-m repeated sprint training on maximum sprinting speed, repeated sprint speed endurance, vertical jump, and aerobic capacity in young elite male soccer players. J Strength Cond Res. 2011;25(9):2364-70.

22. Tous-Fajardo J, Gonzalo-Skok O, Arjol-Serrano JL, Tesch P. Enhancing change-of-direction speed in soccer players by functional inertial eccentric overload and vibration training. Int J Sports Physiol Perform. 2016;11(1):66-73.

23. West DJ, Cunningham DJ, Bracken RM, Bevan HR, Crewther BT, Cook CJ, et al. Effects of resisted sprint training on acceleration in professional rugby union players. J Strength Cond Res. 2013;27(4):1014-8.

24. Winwood PW, Cronin JB, Posthumus LR, Finlayson SJ, Gill ND, Keogh JW. Strongman vs. traditional resistance training effects on muscular function and performance. J Strength Cond Res. 2015;29(2):429-39.
