# Supplementary material for: The Training of Medium- to Long-Distance Sprint Performance in Football Code Athletes: A Systematic Review and Meta-analysis
Source: Sports Med. 2021 Sep 9;52(2):257–86. doi: 10.1007/s40279-021-01552-4 (PMC8803780; doi:10.1007/s40279-021-01552-4)
Supplement: Supplementary file 3 — Supplementary file3 (DOCX 34 kb) [file 40279_2021_1552_MOESM3_ESM.docx]

**Electronic Supplementary Material Table S3**

Article title - The Training of Medium-Long-Sprint Performance in Football Code Athletes: A Systematic Review and Meta-Analysis

Journal name – Sports Medicine

Author names - Ben Nicholson, Alex Dinsdale, Ben Jones, and Kevin Till.

Affiliations - Leeds Beckett University, Carnegie Applied Rugby Research (CARR) centre, Carnegie School of Sport, Leeds, United Kingdom. Yorkshire Carnegie Rugby Union club, Leeds, United Kingdom. Leeds Rhinos Rugby League club, Leeds, United Kingdom. England Performance Unit, The Rugby Football League, Leeds, United Kingdom. School of Science and Technology, University of New England, Armidale, NSW, Australia. Division of Exercise Science and Sports Medicine, Department of Human Biology, Faculty of Health Sciences, the University of Cape Town and the Sports Science Institute of South Africa, Cape Town, South Africa.

corresponding author e-mail address – b.t.nicholson@leedsbeckett.ac.uk

**Table S3**

**Characteristics of the specific sprint training groups (primary, secondary and combined primary and secondary methods) included in the review**

| **Study (year)** | **Subjects** | **Training type and organisation** | **Training methods** | **Other training and testing equipment** | **Mean difference, 95% CI, percentage change, Std. Mean Difference IV, Random, 95% CI, weight, Qualitative inference** |
| --- | --- | --- | --- | --- | --- |
| Borges et al. (2016) A (1) | M, n=9, Elite Soccer Players; Age 16±0.6 years | Resisted sprint training 1-2d/wk, 7wks, 12 sessions, In-season | 2-7 sets of short-medium distance resisted sled sprints (1 rep of at each distance/set 5m; 10m; 20m and 30 m @ 10-13% BW) | 90—150 minutes soccer training session every morning, 5 times/wk, and one official match every Saturday  Photocell system | 0-30m performance = MD (s): 0.05; 95% CI [0, 0.1]; % Change 1.17%;  SMD: 0.25; 95% CI [-0.02, 0.51] Weight 14.76%; Inference - Small |
| Bremec (2018) A (2) | M, n=8, Sub-elite Soccer Players; Age 15.6±0.4 years | Resisted sprint training 2d/wk, 8wks, 16 sessions, Pre-season and In-season | 1 set of 3-5 reps/set of short distance resisted sprints (20m) at loads corresponding to peak power output, resisted using a 1080Sprint | 4 soccer training sessions/wk, 2 longer (75-90 mins) and 2 shorter (45-60 mins) duration and 1 match/wk  1080Sprint (1080 Motion AB, Lidingö, Sweden) | 0-30m performance ↑ MD (s): 0.19; 95% CI [0.12, 0.26]; % Change 3.69%;  SMD: 0.79; 95% CI [0.47, 1.11] Weight 14.06%; Inference - Moderate  Vmax = MD (m·s^-1^): 0.02; 95% CI [-0.1, 0.14]; % Change 0.27%;  SMD: 0.04; 95% CI [-0.18, 0.26] Weight 32.75%; Inference - Trivial |
| Bremec (2018) B (2) | M, n=10, Sub-elite Soccer Players; Age 15.6±0.5 years | Unresisted sprint training 2d/wk, 8wks, 16 sessions, Pre-season and In-season | 1 set of 8 reps/set of short distance (20m) sprints | See Bremec (2018) A | 0-30m performance = MD (s): -0.03; 95% CI [-0.15, 0.09]; % Change -0.55%;  SMD: -0.06; 95% CI [-0.31, 0.19] Weight 16.78%; Inference - Trivial  Vmax = MD (m·s^-1^): 0.08; 95% CI [-0.09, 0.25]; % Change 1.05%;  SMD: 0.09; 95% CI [-0.1, 0.29] Weight 33.95%; Inference - Trivial |
| De Hoyo et al. (2016) B (3) | M, n=12, Elite Soccer Players; Age 17±1 years | Resisted sprint training 2d/wk, 8wks, 16 sessions, In-season | 3 sets of 6–10 reps/set of short loaded (12.6%) sprints (20m) on artificial grass. | 10 hrs of combined soccer (4– 5 sessions) and conditioning (1 session) training, and 1 competitive match/wk.  Dual-beam electronic timing gate OptoJump System (Polifemo Radio Light, Microgate, Bolzano, Italy) | 0-30m performance ↑ MD (s): 0.03; 95% CI [0, 0.06]; % Change 0.72%;  SMD: 0.24; 95% CI [0.01, 0.46] Weight 15.21%; Inference - Small  0->30m performance ↑ MD (s): 0.07; 95% CI [0.02, 0.12]; % Change 1.07%;  SMD: 0.32; 95% CI [0.09, 0.56] Weight 29.9%; Inference - Small |
| Derakhti et al. (2018) A (4) | M, n=8, Soccer Players; Age 15.6±0.4 years | Resisted sprint training 2d/wk, 4wks, 8 sessions, In-season | 1 set of 5 reps/set of short distance (20m) resisted sprints @ a load corresponding to a 50% reduction in Vmax (Pmax) on artificial “astro” turf. | On average four soccer specific sessions/Wk. Two longer sessions (75-90 min) and two were shorter (45-60 min). After the fifth session of the intervention, the participants’ regular game-season began and one competitive match (40+40min) /wk (usually Saturday or Sunday) was added to the total training volume.  1080Sprint (1080 Motion AB, Lidingö, Sweden) | 0-30m performance ↑ MD (s): 0.19; 95% CI [0.12, 0.26]; % Change 3.69%;  SMD: 0.79; 95% CI [0.47, 1.11] Weight 14.06%; Inference - Moderate |
| Derakhti et al. (2018) B (4) | M, n=10, Soccer Players; Age 15.6±0.5 years | Unresisted sprint training 2d/wk, 4wks, 8 sessions, In-season | 1 set of 8 reps/set of short distance sprints (20m) on artificial “astro” turf. | See Derakhti et al. (2018) A | 0-30m performance = MD (s): -0.03; 95% CI [-0.15, 0.09]; % Change -0.55%;  SMD: -0.06; 95% CI [-0.31, 0.19] Weight 16.78%; Inference - Trivial |
| Escobar-Álvarez et al. (2018) A (5) | F, n=8 Elite Rugby union Players; Age 26.8±5 years | Resisted sprint training 2d/wk, 8wks, 16 sessions, Phase not reported | Resisted sprinting @80% BW (2 sets of 5 sprints/set) of moderate distance sprints (30m). | NA  My Sprint mobile application | 0-30m performance ↑ MD (s): 0.7; 95% CI [0.56, 0.84]; % Change 13.46%;  SMD: 1.37; 95% CI [0.98, 1.75] Weight 13.08%; Inference - Large |
| Escobar-Álvarez et al. (2018) B (5) | F, n=8 Elite Rugby union Players; Age 23±3 years | Unresisted sprint training 2d/wk, 8wks, 16 sessions, Phase not reported | Maximal sprinting (2 sets of 5 sprints/set) of moderate distance sprints (30m). | See Escobar-Álvarez et al. (2018) A | 0-30m performance ↑ MD (s): 0.1; 95% CI [0.02, 0.18]; % Change 1.79%;  SMD: 0.33; 95% CI [0.05, 0.62] Weight 15.67%; Inference - Small |
| Majdell and Alexander (1991) A (6) | M, n=6, Elite American football Players; Age 23±2.73 years | Assisted sprint training 3d/wk, 6wks, 16 sessions, Off-season | Supramaximal running speeds using the sprint master towing device. 1-3 sets of 3 reps/sets of medium distance sprints 40m with a 10m run in | N/A  High speed camera | 0->30m performance = MD (s): 0.09; 95% CI [0, 0.18]; % Change 1.72%;  SMD: 0.31; 95% CI [-0.02, 0.64] Weight 15.02%; Inference - Small |
| Majdell and Alexander (1991) B (6) | M, n=6, Elite American football Players; Age 19±1.36 years | Resisted and assisted sprint training 3d/wk, 6wks, 18 sessions, Off-season | Subjects were towed using the sprint master towing device to achieve supramaximal running speeds whilst wearing a 10 pounds weights vest. 1-3 sets of 3 reps/sets of medium distance sprints 40m | See Majdell and Alexander (1991) A | 0->30m performance ↑ MD (s): 0.1; 95% CI [0.05, 0.15]; % Change 1.94%;  SMD: 0.58; 95% CI [0.24, 0.93] Weight 13.45%; Inference - Moderate |
| Majdell and Alexander (1991) C (6) | M, n=6, Elite American football Players; Age 21±3.52 years | Unresisted sprint training 3d/wk, 6wks, 18 sessions, Off-season | Unresisted sprint training 1-3 sets of 3 reps/sets of medium distance sprints 40m | See Majdell and Alexander (1991) A | 0->30m performance = MD (s): 0.04; 95% CI [-0.08, 0.16]; % Change 0.74%;  SMD: 0.1; 95% CI [-0.22, 0.42] Weight 37.38%; Inference - Trivial |
| Meckel et al. (2012) A (7) | M, n=11, Soccer Players; Age 14.5±0.6 years | Unresisted sprint training 3d/wk, 7wks, 21 sessions, Pre-season | 4-6 sets of 4 reps of long-distance sprints (50m) at 85% of maximum speed | Soccer training 4 times/wk  Photoelectric cell timing system (Alge-Timing Electronic, Vienna, Austria) | 0-30m performance ↑ MD (s): 0.12; 95% CI [0.07, 0.17]; % Change 2.53%;  SMD: 0.61; 95% CI [0.35, 0.87] Weight 16.5%; Inference - Moderate |
| Meckel et al. (2012) B (7) | M, n=13, Soccer Players; Age 14.5±0.6 years | Unresisted sprint training 3d/wk, 7wks, 21 sessions, Pre-season | 1 set of 4-6 reps of long-distance sprints (200m) | See Meckel et al. (2012) A | 0-30m performance ↑ MD (s): 0.08; 95% CI [0.03, 0.13]; % Change 1.69%;  SMD: 0.35; 95% CI [0.13, 0.58] Weight 17.5%; Inference - Small |
| Rimmer and Sleivert (2000) B (8) | M, n=9, Sub-elite Rugby + Touch Rugby Players; Age 24.4±4 years | Unresisted sprint training 1-2d/wk, 8wks, 15 sessions, Phase not reported | Unresisted sprinting for medium length sprints 2-5 sets of 2-8 reps/set (25-55 m) on a grass surface. | N/A  Digital timer (University of Otago, Dunedin, New Zealand) | Vmax ↓ MD (s): -0.01; 95% CI [-0.02, 0]; % Change -0.83%;  SMD: -0.21; 95% CI [-0.42, 0] Weight 32.1%; Inference - Small |
| Upton (2011) A (9) | F, n=8 Elite Soccer Players; Age 19.6±0.9 years | Assisted sprint training 3d/wk, 4wks, 12 sessions, Pre-season | Assisted maximal sprinting (1 set of 10 sprints/set) of short sprints (18.3m). Assistive load @14.7%BW (45-95 assistive force). Sprints were assisted by a bungy cord shoulder harness | 6 d/wk (~1.5hrs) ball handling skills, defensive drills, offensive drills, and small sided games.  Infrared beam timing system (Brower Timing Systems Speed Trap II, Salt Lake City, UT, USA) | 0-30m performance ↑ MD (m·s^-1^): 0.12; 95% CI [0.08, 0.16]; % Change 2.08%;  SMD: 0.81; 95% CI [0.49, 1.13] Weight 14.03%; Inference - Large  0->30m performance ↑ MD (m·s^-1^): 0.08; 95% CI [0.03, 0.13]; % Change 1.28%;  SMD: 0.48; 95% CI [0.19, 0.77] Weight 18.83%; Inference - Moderate  Vmax = MD (m·s^-1^): -0.02; 95% CI [-0.09, 0.05]; % Change -0.28%;  SMD: -0.07; 95% CI [-0.28, 0.15] Weight 32.72%; Inference - Trivial |
| Upton (2011) B (9) | F, n=9 Elite Soccer Players; Age 19.6±0.9 years | Resisted sprint training 3d/wk, 4wks, 12 sessions, Pre-season | Resisted maximal sprinting (1 set of 10 sprints/set) of short sprints (18.3m). Resistance load @10% dec in Vmax (12.6%Bw). Sprints were resisted by a "The trainer (Perform better) by a waist harness" | See Upton (2011) A | 0-30m performance = MD (m·s^-1^): 0.03; 95% CI [-0.02, 0.08]; % Change 0.52%;  SMD: 0.17; 95% CI [-0.1, 0.43] Weight 14.79%; Inference - Trivial  0->30m performance ↑ MD (m·s^-1^): 0.06; 95% CI [0, 0.12]; % Change 0.97%;  SMD: 0.27; 95% CI [0, 0.53] Weight 22.8%; Inference - Small  Vmax ↑ MD (m·s^-1^): 0.07; 95% CI [0, 0.14]; % Change 0.97%;  SMD: 0.22; 95% CI [0.01, 0.43] Weight 34.52%; Inference - Small |
| Upton (2011) C (9) | F, n=10 Elite Soccer Players; Age 19.6±0.9 years | Unresisted sprint training 3d/wk, 4wks, 12 sessions, Pre-season | Maximal sprinting (1 set of 10 sprints/set) of short sprints (18.3m). | See Upton (2011) A | 0-30m performance = MD (m·s^-1^): 0.02; 95% CI [-0.06, 0.1]; % Change 0.35%;  SMD: 0.07; 95% CI [-0.18, 0.31] Weight 16.78%; Inference - Trivial  0->30m performance = MD (m·s^-1^): 0.01; 95% CI [-0.07, 0.09]; % Change 0.16%;  SMD: 0.03; 95% CI [-0.22, 0.28] Weight 62.62%; Inference - Trivial  Vmax = MD (m·s^-1^): -0.04; 95% CI [-0.12, 0.04]; % Change -0.55%;  SMD: -0.09; 95% CI [-0.29, 0.1] Weight 33.95%; Inference - Trivial |

M = male, F = female, N/A = data not available, short sprints = 0-≤20m, medium sprints = 0-≤40m, long sprints 0->40m, SMD = standardised mean difference, CI = confidence interval, MD = mean difference, % Change = percentage change, d = day, wk(s) = week(s), hr(s) = hour(s), ↑ = significant increase in sprint performance (p = < 0.05) , = = no significant change in sprint performance (p = > 0.05), ↓ = significant decrease in sprint performance (p = < 0.05).

# Declarations

**Ethics**

Approval was obtained from the ethics committee of Leeds Beckett University. The procedures used in this study comply with the ethical standards of the Declaration of Helsinki.

**Consent for publication**

Not applicable

**Availability of data and materials**

The datasets generated during and/or analysed during the current study are available from the corresponding author on reasonable request.

**Funding**
No sources of funding were used to assist in the preparation of this article.

**Conflicts of interest**

Ben Nicholson, Alex Dinsdale, Ben Jones and Kevin Till declare no potential conflicts of interest concerning the research, content, authorship, and/or publication of this review.

**Authors' contributions**

All the authors contributed to the manuscript, including the conception and design of the study, analysis and interpretation of the data, drafting and critically revising the manuscript, and approval for publication. All authors read and approved the final manuscript.

# References

1. Borges JH, Conceição MS, Vechin FC, Pascoal EHF, Silva RP, Borin JP. The effects of resisted sprint vs. plyometric training on sprint performance and repeated sprint ability during the final weeks of the youth soccer season. Sci Sports. 2016;31(4):e101-e5.

2. Bremec D. Very heavy resisted sprinting: a better way to improve acceleration?: effects of a 4-week very heavy resisted sprinting intervention on acceleration, sprint and jump performance in youth soccer players [Masters Thesis]: Swedish School of Sport and Health Sciences; 2018.

3. de Hoyo M, Gonzalo-Skok O, Sañudo B, Carrascal C, Plaza-Armas JR, Camacho-Candil F, et al. Comparative effects of in-season full-back squat, resisted sprint training, and plyometric training on explosive performance in U-19 elite soccer players. J Strength Cond Res. 2016;30(2):368-77.

4. Derakhti M. Very heavy resisted sprint training for adolescent football players: a training intervention on acceleration, sprint and jump performance in late pubertal adolescent athletes [Masters of sports science]: Swedish School of sport and health science; 2018.

5. Escobar-Álvarez J, Fuentes-García J, Pérez-Sousa M, Calleja-González J. Effect of very heavy sled training in speed performance (30m) in female rugby union players. ICTS VI International Conference in Team Sports; España. 2018. p. 1.

6. Majdell R, Alexander M. The effect of overspeed training on kinematic variables in sprinting. J Hum Mov Stud. 1991;21(1):19-39.

7. Meckel Y, Gefen Y, Nemet D, Eliakim A. Influence of short vs. long repetition sprint training on selected fitness components in young soccer players. J Strength Cond Res. 2012;26(7):1845-51.

8. Rimmer E, Sleivert G. Effects of a plyometrics intervention program on sprint performance. J Strength Cond Res. 2000;14(3):295-301.

9. Upton DE. The effect of assisted and resisted sprint training on acceleration and velocity in Division IA female soccer athletes. J Strength Cond Res. 2011;25(10):2645-52.
